# The Evaluation of Pharmacodynamics and Pharmacokinetics of Anti-thrombin DNA Aptamer RA-36

**DOI:** 10.3389/fphar.2017.00922

**Published:** 2017-12-14

**Authors:** Elena Zavyalova, Nadezhda Samoylenkova, Alexander Revishchin, Askar Turashev, Ilya Gordeychuk, Andrey Golovin, Alexey Kopylov, Galina Pavlova

**Affiliations:** ^1^Department of Chemistry, Lomonosov Moscow State University, Moscow, Russia; ^2^Apto-Pharm Ltd., Moscow, Russia; ^3^Institute of Gene Biology, Russian Academy of Sciences, Moscow, Russia; ^4^Chumakov Federal Scientific Center for Research and Development of Immune-and- Biological Products (RAS), Moscow, Russia; ^5^I.M. Sechenov First Moscow State Medical University, Moscow, Russia; ^6^Department of Bioengineering and Bioinformatics, Lomonosov Moscow State University, Moscow, Russia

**Keywords:** anticoagulant, blood coagulation, DNA aptamer, bivalirudin, inhibitor, preclinical trials, thrombin, pharmacokinetics

## Abstract

Anticoagulants are a vital class of drugs, which are applied for short-term surgical procedures, and for long-term treatments for thrombosis prevention in high risk groups. Several anticoagulant drugs are commercially available, but all have intrinsic disadvantages, e.g., bleeding risks, as well as specific ones, e.g., immune response to peptide/protein drugs. Therefore, the search for novel, efficient and safe anticoagulants is essential. Nucleic acid aptamers are an emerging class of contemporary pharmaceuticals which are fully biocompatible and biodegradable; they have low toxicity, and are as efficient as many protein-based drugs. The anti-thrombin DNA aptamer RA-36 has been created using a combination of rational design and molecular dynamics, showing several extra-features over existing aptamers. Aptamer RA-36 has a bimodular structure; the first G-quadruplex binds and inhibits thrombin, whereas the second G-quadruplex varies the properties of the first. This bimodular structure provides a favorable dose-effect dependence allowing the risk of bleeding to be potentially decreased. Here, the results of efficiency trials of the aptamer are presented. The aptamer RA-36 has a distinctive species specificity; therefore, the careful selection of experimental animals was required. The anticoagulant activity was characterized in rats and monkeys *in vivo*. Antithrombotic activity was evaluated in the live murine model of the induced thrombosis. Pharmacokinetics was estimated by tracking radionuclide labeled aptamer in rats. The aptamer was thoroughly characterized using bivalirudin as a reference drug. Despite the different profiles of anticoagulant activity, these two compounds could refer to each other, and the corresponding doses could be estimated. Bivalirudin turned out to have 10-fold higher anticoagulant and antithrombotic activity. The difference in activity is easy to explain due to the pharmacokinetic profiles of the substances: the aptamer RA-36 has 20-fold faster elimination from blood with a half-life of 1 min. The entire dataset revealed that the non-modified DNA aptamer could be an alternative to the currently used bivalent peptide inhibitor; the dosage profile could be improved by manipulating aptamer pharmacokinetics. The study has revealed aptamer RA-36 to be one of the most promising candidates for further development as a new generation of anticoagulants.

## Introduction

Hemostasis requires a dozen tightly interconnected processes. In the case of a vessel injury, a narrow fiber net with incorporated platelets must be quickly formed to prevent blood loss. Impairment of the processes of coagulation and platelet aggregation leads to excessive thrombus formation, which is dangerous for the body and requires therapy. Another requirement for antithrombotic therapy is during surgical procedures and the post-operative period. Anticoagulants, in particular thrombin inhibitors, are one of the main classes of therapeutic tools for hemostasis correction (Monroe and Hoffman, 2006; Harter et al., 2015).

A repertoire of compounds of different nature has been developed to inhibit thrombin, including substances with low molecular weight (argatroban, dabigatran), oligosaccharides (heparin family), and proteins (hirudins, bivalirudin). Despite the apparent diversity of marketed drugs, the search for an ideal anticoagulant is still a very hot issue. The main complications are the following: non-controllable action causes a high frequency of bleeds, and toxicity/side-effects/immunogenicity that are largely dependent on the chemical nature of the drug (Corral-Rodrıguez et al., 2010; Denas and Pengo, 2013; Pudusseri et al., 2013; Treschan et al., 2014). The first complication can be overcome with the development of antidotes (Fernández and Vargas-Castrillón, 2014; Tummala et al., 2016). To surmount the protein complications, new classes of substances are required, e.g., with different backbone like nucleic acid-based compounds (Woodruff and Sullenger, 2015; Zavyalova et al., 2016a).

Nucleic acid aptamers are oligonucleotides, a special form of which provides a high affinity and specificity of target binding. The possibility of the development of aptamers to any target has stimulated attention, focusing on aptamers as affordable molecular recognition elements to solve multiple tasks of fundamental and applied sciences. Nucleic acids, as the basis of the aptamers, provide biocompatibility and biodegradability; therefore, aptamers are considered a promising class of pharmaceuticals. Compared with the close functional analog, antibodies, aptamers have some advantages stemming from their chemical nature. The most important property of aptamers is the existence of rational antidotes; simple complementary oligonucleotides readily transform an active aptamer conformation into the inactive double-stranded helix. The significance of antidote availability for anticoagulants is curiously highlighted by the recent development of nucleic acid aptamers to the peptide bivalirudin as an antidote (Martin et al., 2013). Non-modified aptamers, with just four native nucleotides, have extremely low toxicity and immunogenicity; they quickly degrade to natural non-toxic metabolites *in vivo*. All of these properties clearly show that aptamers are next generation therapeutics with an extremely high efficacy/risk ratio (Potti et al., 2004; Shigdar et al., 2010; Fernández and Vargas-Castrillón, 2014; Zavyalova et al., 2016a).

The first nucleic acid aptamer to thrombin was reported in Bock et al. (1992). A dozen DNA and RNA aptamers to thrombin have since been developed, being adopted to a large variety of applications in biochemistry, bioanalytics and medicine. Two DNA aptamers to thrombin, HD1 and NU172, successfully passed preclinical trials; they entered clinical trials as agents for the treatment of cardiovascular diseases, but no drug has been developed to date (Woodruff and Sullenger, 2015; Nimjee et al., 2016; Zavyalova et al., 2016a,b). The trials of aptamer HD1 were terminated due to suboptimal dosing profiles in humans. Optimized second-generation aptamers have been developed to gain higher efficiency (Schwienhorst, 2006; Lancellotti and De Cristofaro, 2009). NU172 was assumed to be administered by continuous infusion during cardiopulmonary bypass or other surgical procedures to maintain a proper state of anticoagulation. The aptamer had entered phase II of clinical trials; and it is currently suspended (Keefe et al., 2010; Denas and Pengo, 2013).

A special case, DNA aptamer RA-36, has a bimodular structure made from two G-quadruplexes: the first provides inhibitory activity toward thrombin, whereas the second modulates the properties of the first G-quadruplex. This unique construction imparts on the aptamer a special pharmacological profile compared to its counterparts, aptamers HD1 and NU172. Thus, the anticoagulant activity of RA-36 has a sigmoid dose-dependence that increases the efficacy/risk ratio; thus, bleeding risks could be decreased (Zavyalova et al., 2011, 2012, 2013).

Here, preclinical tests of the efficiency of aptamer RA-36 are reported. The peptide counterpart, bivalirudin, was chosen as a reference drug, because it is close due to the pharmacokinetics and pharmacodynamics, and is already used in the clinic. In addition, both compounds are direct thrombin inhibitors; they are administered intravenously, and have a short lifetime in blood (Zavyalova et al., 2016b). Anticoagulant efficiency was assessed *in vivo* after both single and repeated intravenous injections into rats and monkeys. Moreover, the antithrombotic efficiency of both compounds was visually estimated in a live murine model of induced thrombosis. Finally, pharmacokinetics of aptamer RA-36 was measured by tracking radionuclide labeled RA-36 in rats.

## Materials and Methods

### Materials

DNA aptamer RA-36, dGGTTGGTGTGGTTGGTGGTTGGTGTGGTTGG⋅2 KCl, was synthesized and purified up to pharmaceutical grade for intravenous injections at ‘APTO-PHARM,’ Ltd. Russian Federation. The drug formulation for intravenous injections contained 10 mg/ml of the active substance in 0.9% sodium chloride solution; to prepare that formulation, the aptamer stock solution was diluted with 0.9% sodium chloride immediately before the intravenous injection, when proper dosing is needed.

Lyophilized bivalirudin (Angiox^®^, lot# PL2097) was purchased from Medicines Company, United Kingdom. The powder was dissolved in 0.9% sodium chloride at a concentration of 5 mg/ml according to the manufacturer’s guidelines. The stock solution was diluted with 0.9% sodium chloride immediately before intravenous injection, when proper dosing is needed.

Standard human plasma, Test Thrombin Reagent, Thromborel^®^ S and Actin FS Activated PTT Reagent were purchased from Siemens, Germany. ^32^P-labeled ATP with beta radiation activity of 20 MBq was purchased from Perkin-Elmer, United States. T4 polynucleotide kinase (Sigma–Aldrich, United States).

### Assessment of Anticoagulant Activity of Aptamer RA-36 *in Vitro*

All procedures were conducted in accordance with the standards set forth in the EU Directive 2010/63/EU. All animal care and experimental procedures were approved by the Ethics Committee of Moscow State University (Permit Number 34-07).

Three male Sprague Dawley rats (2 month old animals with weight about 250 g) were supplied from the Nursery of the Russian Academy of Medical and Technological Sciences, Moscow, Russian Federation. Ten male C57Bl/6 mice (12 weeks old animals with weight about 30 g) were supplied from the Experimental Animals Unit of Blokhin Russian Cancer Research Center. Three male rabbits (6 month old animals with weight about 3 kg) and three male guinea pigs (2 month old animals with weight about 350 g) were supplied with Nursery of the Russian Academy of Medical and Technological Sciences, Moscow, Russian Federation. Two male *Macaca mulatta* (6–7 years old animals with weight about 6–8 kg) were provided by Scientific Research Institute of Medical Primatology, Sochi, Russian Federation. Two male *Callithrix jacchus* (3 years old animals with weight about 300 g) were provided by Chumakov Institute of Poliomyelitis and Viral Encephalitis, Moscow, Russia.

In the case of rats, mice and guinea pigs, animals were anaesthetized with chloral hydrate, and blood samples were acquired from jugular vein. In the case of rabbits, blood samples were acquired from the regional vein of an ear without narcotization of the animal. In the case of monkeys, blood samples were acquired from the femoral vein without narcotization of the animal.

Blood samples from mouse, rat, rabbit, guinea pig, *Macaca mulatta* and *Callithrix jacchus* were mixed with 3.8% sodium citrate solution (9:1 by volume), centrifuged for 10 min at 1.5 krpm, and the supernatant was then collected. Plasma samples from different animals within the species was mixed together. The prothrombin time test was used to characterize the anticoagulant activity of aptamer RA-36. The concentration of aptamer RA-36 in plasma samples was 5 μM.

### Maintenance of Experimental Animals

In total, 140 male Sprague Dawley rats were used to assess anticoagulant activity *in vivo*. Animal mass was about 200–250 g (2 month old rats). Young rats tolerated well both continuous anesthesia and repeated blood sampling in our preliminary experiments. Animals were supplied from the Nursery of the Russian Academy of Medical and Technological Sciences, Moscow, Russian Federation. They were maintained in a standard laboratory animal facility with free access to feed and water, a reverse 12 h: 12 h light: dark cycle, at 18–20°C and 55–65% humidity. The animals were acclimatized to these conditions for at least 2 weeks before the start of the experiments. To minimize suffering, rats were euthanized by one intravenous injection of 3 g/kg urethane after the experiments with blood sampling.

Next, 56 monkeys (28 male and 28 female *Callithrix jacchus*) were used to assess anticoagulant activity *in vivo*. Animal mass was in the range of 220–570 g, and age was in the range of 1–12 years. Animals were supplied from Experimental Clinic of Callitrichidae monkeys Department of Viral Hepatitis, Chumakov Institute of Poliomyelitis and Viral Encephalitis, Moscow, Russia. Monkeys were accommodated in latticed cages, equipped with cabins both for sleeping and for encouraging natural behavior. Animals had free access to feed and water, a reverse 12 h:12 h light: dark cycle, at 27–29°C and 70–75% humidity. The animals were acclimatized to these conditions for at least 40 days before the start of the experiments.

Twenty male Sprague Dawley rats were used in the pharmacokinetic study. Animal mass was 450–500 g (6 months old). Grown rats were chosen for pharmacokinetic experiments due to the higher weight of the animals, and, hence, increased dose of labeled aptamer per animal providing a possibility to study the distribution between tissues with higher accuracy. As the aptamer is to be administered intravenously in the pharmacologically active form with short-term action, the age of the adult animals is expected to have a minor effect on the pharmacokinetics and pharmacodynamics of the aptamer. The rats were acquired from Nursery of Laboratory Animals ‘Pushchino,’ Moscow, Russian Federation. They were maintained in a standard laboratory animal facility with free access to feed and water, a reverse 12 h:12 h light: dark cycle, at 22–26°C and 35–75% humidity. The animals were acclimatized to these conditions for at least a week before the start of the experiments. To minimize suffering, rats were euthanized by one intravenous injection of 3 g/kg urethane after the experiments with blood sampling. Rats for organ uptake were euthanized by cervical dislocation.

A total of 65 male C57Bl/6 mice (25–31 g, and 12 weeks old) used in the experiments were supplied from the Experimental Animals Unit of Blokhin Russian Cancer Research Center. They were maintained in a standard laboratory animal facility with free access to feed, water, a reverse 12 h: 12 h light: dark cycle and were acclimatized to these conditions for at least 2 weeks before the start of the experiments. To minimize suffering, after the experiments, mice were euthanized by the intramuscular injection of 150 mg/kg ketamine.

### Assessment of Anticoagulant Activity *in Vivo*

**FIGURE 1:**
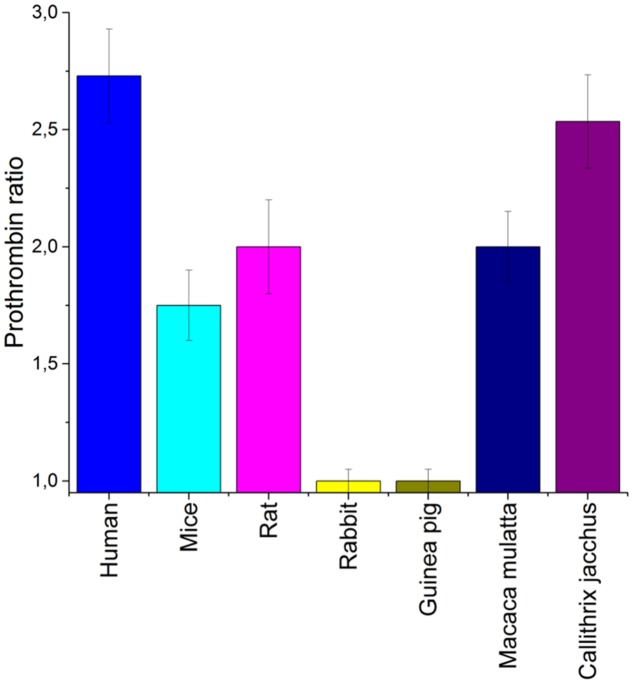
An estimation of a species specificity of aptamer RA-36. Anticoagulant effect was estimated for various blood plasma samples. Normalized values from PT test were used to compare different species. Concentration of aptamer RA-36 was 5 μM in all plasma samples. Standard deviations are shown.

A proposed therapeutic dose for aptamer RA-36 has been experimentally selected as 7.0 mg/kg = 0.70 μmole/kg, because it increased the coagulation time 2 to 3-fold in PT and APTT tests that is common for anticoagulants (Zavyalova et al., 2016a). The general tendency for the dose selection for different animals was taken from the *in vitro* tests on animal plasma (**Figure 1**); doses were increased in the raw: mice, rats, monkeys. The exact values for the doses were chosen tentatively.

#### Single Bolus Injections into Rats

Seventy rats were randomly divided into seven groups (10 rats per a group). Animals were anaesthetized with 400 mg/kg chloral hydrate. Jugular veins were denuded, and 200 μl of a sample was bolus injected with insulin syringe using a 29G needle. The sample content in groups was as follows: (1) 0.9% sodium chloride; (2) bivalirudin, 0.19 mg/kg; (3) bivalirudin, 0.38 mg/kg; (4) bivalirudin, 0.75 mg/kg; (5) aptamer RA-36, 7 mg/kg; (6) aptamer RA-36, 21 mg/kg; and (7) aptamer RA-36, 42 mg/kg. Then, 270 μl of blood was collected from jugular vein with insulin syringe with 29G needle. The syringe contained 30 μl of 3.8% sodium citrate solution. The time intervals of blood collection were identical for all groups: 5 min before and 2, 5, 10, 15, 30, and 60 min after the injection of the sample.

#### Single Bolus Injections into Monkeys

The 56 monkeys were randomly divided into seven groups (8 monkeys per group). Then, 200 μl of the sample was bolus injected with insulin syringe with 29G needle in femoral vein. The sample content in groups was the following: (8) 0.9% sodium chloride; (9) bivalirudin, 0.19 mg/kg; (10) bivalirudin, 0.38 mg/kg; (11) bivalirudin, 0.75 mg/kg; (12) aptamer RA-36, 7 mg/kg; (13) aptamer RA-36, 14 mg/kg; and (14) aptamer RA-36, 28 mg/kg. In total, 270 μl of blood was collected from femoral vein with an insulin syringe using a 29G needle. The syringe contained 30 μl of 3.8% sodium citrate solution. The time intervals of blood collection were identical for all groups: 5 min before and 2, 5, 10, 15, 30, and 60 min after the injection of the sample.

#### Recurring Bolus Injections into Rats

Seventy rats were randomly divided into seven groups (10 rats per group). Animals were anaesthetized with chloral hydrate in dose of 400 mg/kg. Jugular veins were denuded, and 200 μl of sample was bolus injected with insulin syringe with 29G needle. The sample content in groups was the following: (15) 0.9% sodium chloride; (16) bivalirudin, 0.19 mg/kg; (17) bivalirudin, 0.38 mg/kg; (18) bivalirudin, 0.75 mg/kg; (19) aptamer RA-36, 7 mg/kg; (20) aptamer RA-36, 21 mg/kg; and (21) aptamer RA-36, 42 mg/kg. The same doses were injected repeatedly in the following timelines after the first injection: groups 15, 19–21 – 15, 30, 45, and 60 min; groups 16–18 – 33 min. The intervals were chosen due to the pharmacokinetics of the substances. Here, 270 μl of blood was collected from jugular vein with insulin syringe using a 29G needle. The syringe contained 30 μl of 3.8% sodium citrate solution. The time intervals of blood collection were identical for all groups: 5 min before the first injection of the sample, and 7, 15, 30, 45, 65, and 90 min after.

#### Estimation of the Anticoagulant Effect

Samples of citrated blood were centrifuged for 15 min at 1400 rpm to separate plasma from blood cells. Coagulation of blood plasma was estimated with coagulometer CA-50 (Sysmex, Japan) using the reagent sets from Siemens, Germany: Test Thrombin Reagent for thrombin time (TT) determination, Thromborel^®^ S for prothrombin time (PT) determination, Actin FS Activated PTT Reagent for activated partial thromboplastin time (APTT) determination. Standard Human Plasma was used to test the functional activity of the reagents. Aptamer RA-36 and bivalirudin prolong coagulation time of blood plasma due to thrombin inhibiting. The data were treated with Origin 8.1 (OriginLab, United States). Descriptive statistics was performed, including mean values, geometrical mean values, standard deviations, coefficients of variation, medians, lower 90% CI of mean and upper 90% CI of mean.

### Murine Model of Thrombosis

The murine model of thrombosis has been described previously (Zavyalova et al., 2014). Briefly, mice were anaesthetized with a ketamine-xylazine mixture (80 + 20 mg/kg); lidocaine was used for a local anesthesia at the site of the surgery. A sample of 100 μl of 0.9% NaCl, or aptamer RA-36 (7, 35, and 70 mg/kg dose), or bivalirudin (0.19, 0.38, and 0.75 mg/kg dose) was bolus injected into the jugular vein. A thin steel needle was contacted with the denuded carotid artery; the second needle was introduced subcutaneously into the hip of the mouse. Both needles were used as the electrodes connected to battery of the constant current (voltage – 3 V, amperage – 200–250 mA) for 60–120 s after the sample injection. Thrombus growth at the contact area between the needle and artery was monitored using digital camera Nikon D5100 during 3–40 min after the sample injection. The video was analyzed using software Image Tool; and area of the thrombus was calculated. The data were treated with Origin 8.1 (OriginLab., United States). Descriptive statistics was performed, including mean values and standard deviations.

### Labeling Aptamer RA-36 for the Pharmacokinetic Study

Aptamer RA-36 was labeled with ^32^P radionuclide to track biodistribution and elimination of RA-36 *in vivo*. The label was transferred from ^32^P-labeled ATP to 5′-end of the aptamer using T4 polynucleotide kinase in buffer solution (40 mM Tris-HCl, pH 7.5, 10 mM MgCl_2_, 5 mM dithiothreitol). Labeled aptamer was purified from radioactive ATP using exchange columns Poly-Pak (Glen Research, United States). Labeled aptamer was mixed with unlabeled aptamer to achieve 2 × 10^6^ cpm in the injected dose.

### Pharmacokinetic Study Protocol

#### Experiments with Blood Sampling

Ten rats were implanted with catheters 2 days before pharmacokinetic experiments. Rats were anaesthetized with 30 mg/kg Zoletil and 3 mg/kg xylazine. The femoral vein was denuded and catheterized with 0.6 mm polyethylene catheter. The catheter was filled with heparin solution (100 U/ml). The incision was sewn, and prepared rats were held in the individual cages.

Two days after surgery, ^32^P-labeled aptamer RA-36 was bolus injected in the lateral vein of the tail with G29 needles in 7 mg/kg dose. 200 μl of blood was taken from the catheter 1, 3, 5, 7, 10, 15, 20, 30, and 40 min after aptamer injection. The samples were placed in plastic tubes and labeled.

#### Experiments with Tissue and Organ Uptake

Ten rats were euthanized 3 min after aptamer intravenous injection (three times of half-distribution of the substance from blood). Blood, brain, heart, aorta, lungs, trachea (with thyroid gland), spleen, thymus, adrenal glands, right and left kidneys, bladder, esophagus, stomach, large intestine, small intestine, liver, pancreas, muscle (the front side of the hip), right and left testis, right and left appendages of the testis, prostate, skin (from the withers), and epididymal fat were collected. Hollow organs were opened and cleared of their content. Blood was removed from highly perfused organs. The mass of all samples was determined with an accuracy of ±1 mg.

#### ^32^P-Radioactivity Measurements

Isolated tissues and organs were solubilized in 1 ml of Solvable (Perkin-Elmer, United States) for 3 h at 60°C and subsequent incubation at room temperature. The volume of the sample was adjusted to 10 ml with liquid for scintillation Emulsifier-Safe (Perkin-Elmer, United States).

^32^P-radionuclide content in the samples was determined with scintillation counter Tracore 2000 until the standard deviation reaches ≤ 0.25% from the mean value. Calibration samples were concurrently measured; these samples were obtained by mixing blood samples with known amount of ^32^P-labeled aptamer. Linear dependence of the signal from aptamer content was obtained for experimental sample assessment.

#### Pharmacokinetic Data Treatment

The data were treated with Origin 8.1 (OriginLab, United States). Pharmacokinetics was described with the following equation: C = A ⋅ e^-α⋅x^ + B ⋅ e^-b⋅x^. Experimental data approximation was performed using the method of least squares for the whole dataset with $\frac{1}{y^{2}}$ as the weighting factor. The following parameters were estimated: the area under the curve (extrapolated to infinity by the slope of the final portion of the curve), clearance, volume of distribution, average hold time, constant of elimination.

## Results

### A Search for an Adequate Animal Models

As aptamers to thrombin are known to have a high species specificity (Zavyalova et al., 2012), a preliminary estimation of anticoagulant efficiency in the blood plasma of different animals is required. Mice, rats, rabbits, guinea pigs, and monkeys are conventional experimental animals in which to test anticoagulants. **Figure 1** shows that both rabbit and guinea pig blood plasma coagulation was not affected by aptamer RA-36. The coagulation of the plasma of other animals was inhibited with a somewhat lower efficiency than human plasma. Therefore, mice, rats, and monkeys (*Callithrix jacchus*) were chosen for the further experiments.

### Single Bolus Injection of Aptamer RA-36 and Bivalirudin

Aptamer RA-36 and bivalirudin were bolus injected intravenously into rats and monkeys, and anticoagulant activities were estimated in plasma samples taken during the first hour after injection. Initially, doses for bolus injection, regarding anticoagulant activity, were as follows: the recommended therapeutic dose for bivalirudin is 0.75 mg/kg = 0.34 μmole/kg; a proposed therapeutic dose for aptamer RA-36 is 7.0 mg/kg = 0.70 μmole/kg, which increased the coagulation time 2 to 3-fold in PT and APTT tests. For the experiments in rats, the following doses were used along with the doses mentioned above: bivalirudin doses were decreased 2- and 4-fold compared to the recommended dose, and aptamer RA-36 doses were increased 3- and 6-fold compared to the proposed dose.

**FIGURE 2:**
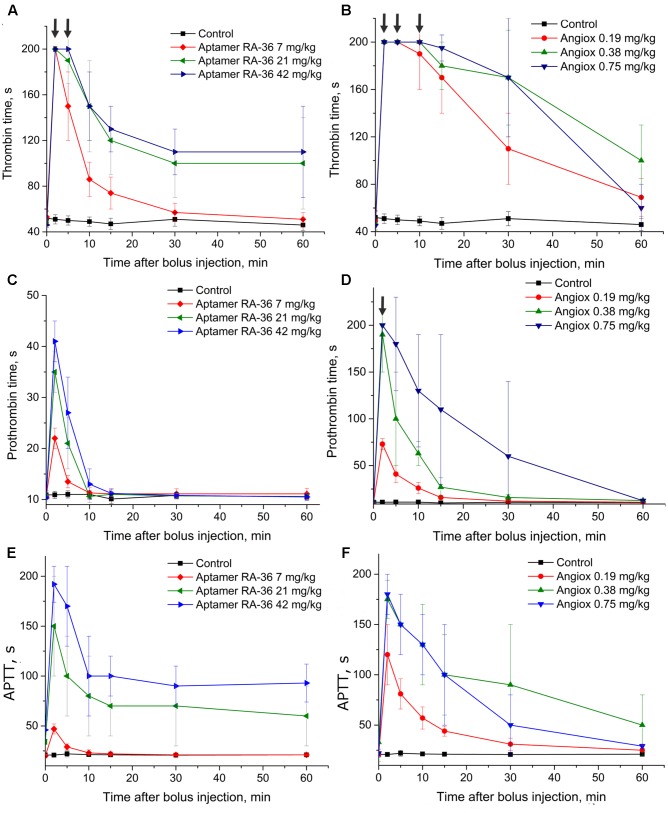
Anticoagulant activity in plasma samples taken from rats with single bolus injection of aptamer RA-36 (**A,C,E**; 7, 21, and 42 mg/kg doses; 10 animals per dose), as well as bivalirudin (**B,D,F**; 0.19, 0.38, and 0.75 mg/kg doses; 10 animals per dose). **(A,B)** TT test; **(C,D)** PT test; **(E,F)** APTT test. Data points with clotting time ≥ 200 s are marked with arrows; the points corresponds non-clotting during the measurement limit of coagulometer.

**Figure 2** shows that the APTT test resulted in a similar efficiency of 0.75 mg/kg for bivalirudin dose and 42.0 mg/kg for RA-36 dose. The PT test revealed a moderate activity of the aptamer which is quite different from the behavior of bivalirudin. Finally, the TT test turned out to be oversensitive in the first minutes for both anticoagulants; the samples were not clotted during 200 s, which is the measurement limit of the coagulometer.

**FIGURE 3:**
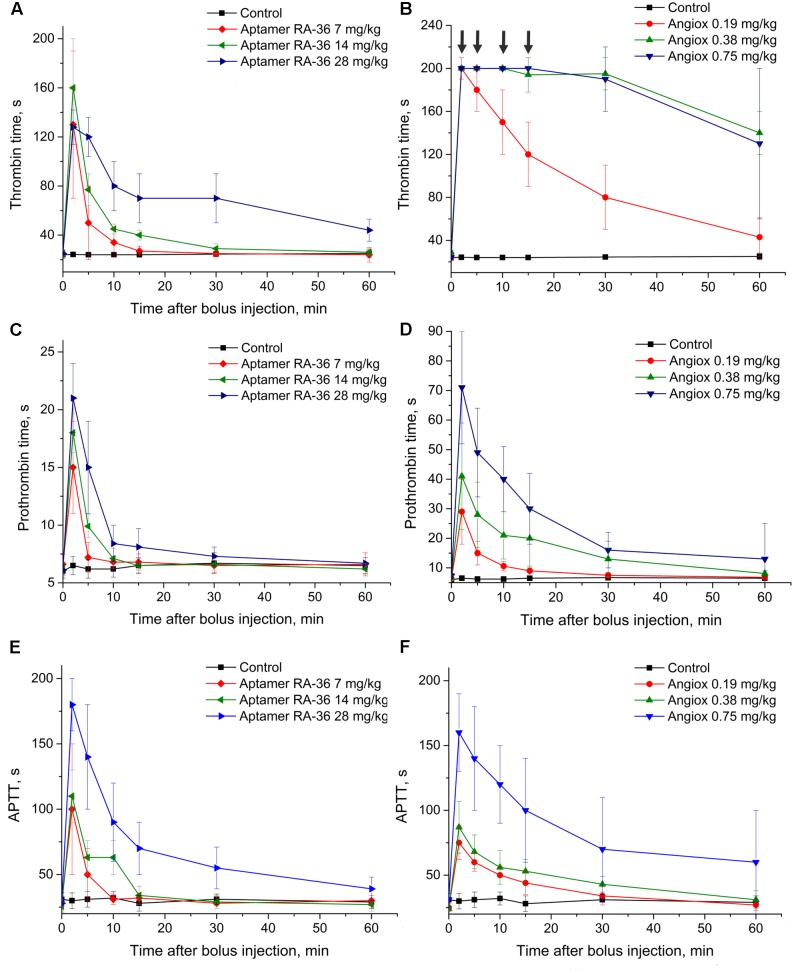
Anticoagulant activity in plasma samples taken from monkeys with single bolus injection of aptamer RA-36 (**A,C,E**; 7, 14, and 28 mg/kg doses; 8 animals per dose) as well as bivalirudin (**B,D,F**; 0.19, 0.38, and 0.75 mg/kg doses; 8 animals per dose). **(A,B)** TT test; **(C,D)** PT test; **(E,F)** APTT test. Data points with clotting time ≥ 200 s are marked with arrows; the points corresponds non-clotting during the measurement limit of coagulometer.

**Figure 3** illustrates similar data taken for monkeys. The doses of bivalirudin were the same, whereas RA-36 was injected in the proposed therapeutic dose for aptamer RA-36 7.0 mg/kg = 0.70 μmole/kg, 2-fold higher dose and 4-fold higher dose. These doses were chosen from the preliminary estimation of the anticoagulant activity of RA-36 in monkey plasma compared to human and rat plasma. In the APTT test 28 mg/kg dose of RA-36 showed approximately the same efficiency as a 0.75 mg/kg dose of bivalirudin.

### Pharmacokinetics for Aptamer RA-36

**FIGURE 4:**
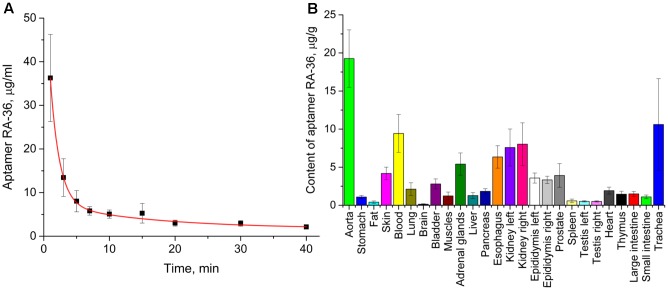
Pharmacokinetics of intravenously bolus injected aptamer RA-36 in rats; dose 7.0 mg/kg. **(A)** Elimination of the aptamer from the blood (eight rats in the group); **(B)** distribution of the aptamer among organs and tissues, 3 min after intravenous injection (10 rats in the group).

The pharmacokinetic study was conducted for the proposed therapeutic dose of aptamer RA-36 7.0 mg/kg. Aptamer was intravenously bolus injected into rats, and traced due to ^32^P-radionuclide label covalently attached to the end. RA-36 was rapidly eliminated from the bloodstream (**Figure 4A**). Integral pharmacokinetic parameters are listed in **Table 1**. Elimination of RA-36 from blood was successfully described with the two compartment model (**Table 2**). The half-life of the aptamer in blood was only 1 min, whereas it was 23 min in tissues.

**Table 1:** Integral pharmacokinetic parameters for elimination of aptamer RA-36 from blood.

Parameter	*AUC_0-40_, μg*⋅min/ml	*AUC_0-∞_, μg⋅*min/ml	*AUMC_0-∞_*, mg⋅min^2^/ml	*MRT*, min	*V_ss_*, ml	*Cl*, ml/min	*k_el_*, min^-1^
Mean value ± SD	240 ± 40	400 ± 50	13 ± 6	38 ± 3	680 ± 130	18 ± 2	0.027 ± 0.003

*SD, standard deviation; AUC, area under the curve; AUMC, total area under the first moment curve; MRT, mean residence time; V_ss_, apparent volume of distribution at steady-state; Cl, total clearance; k_el_, first-order rate constant for elimination.*

**Table 2:** Pharmacokinetic parameters of two compartment model of elimination of aptamer RA-36 from blood.

Parameter	*A*, μg/ml	*B*, μg/ml	*α*, min^-1^	*β*, min^-1^	*T_α_*, min	*T_β_*, min		
Mean value (lower and upper 95% CI of mean)	59 (37–81)	6.9 (5.5–8.2)	0.70 (0.50–0.90)	0.030 (0.022–0.038)	1.0 (0.8–1.4)	23 (18–32)		

**Parameter**	***k_12_*, min^-1^**	***k_21_*, min^-1^**	***k_el_*, min^-1^**	***V_1_*, ml**	***V_2_*, ml**	***V_ss_*, ml**	***AUC_0-∞_*, μg⋅min/ml**	***Cl*, ml/min**

Estimated value	0.42	0.10	0.21	110	750	550	320	22

*CI, confidence interval; V_i_, apparent volumes of distribution in central compartment (i = 1), peripheral compartment (i = 2), and at steady-state (i = ss); k_i_, first-order rate constant for distribution (i = 12), redistribution (i = 21), and elimination (i = el).*

Distribution of the aptamer between tissues and organs was studied at 3 min after intravenous injection. At this time, the percentage of RA-36 in the blood is approximately 15% of the initial value. **Figure 4B** illustrates that the highest relative content of the aptamer was in the aorta, trachea (with thyroid gland), kidneys, esophagus, and adrenal glands.

### Repeated Bolus Injections of Aptamer RA-36 and Bivalirudin

The half-life times of aptamer RA-36 and bivalirudin differ substantially; therefore, different protocols were used for repeated bolus intravenous injections. To provide the anticoagulant effect during 1 h, aptamer RA-36 was bolus injected five times: at 0, 15, 30, 45, and 60 min; and bivalirudin was administered twice: at 0 and 30 min. These intervals of anticoagulant administration correspond to the times of disappearance of anticoagulant effect in the single bolus experiments (**Figures 2C,D**).

**FIGURE 5:**
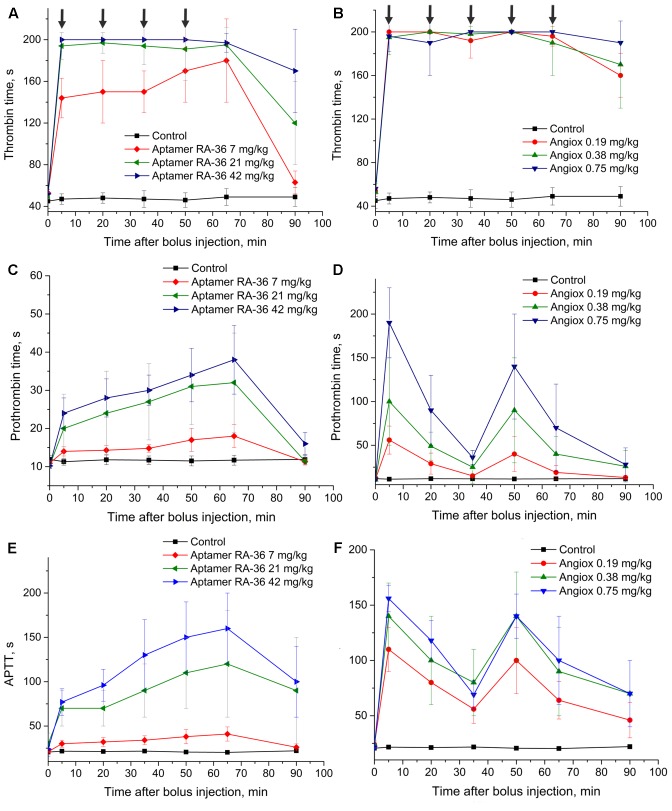
Anticoagulant activity in plasma samples taken from rats with repeated bolus injection of aptamer RA-36 (**A,C,E**; 7, 21, and 42 mg/kg doses every 15 min during 1 h; 10 animals per dose) as well as bivalirudin (**B,D,F**; 0.19, 0.38, and 0.75 mg/kg doses at 0 and 30 min of the experiment; 10 animals per dose). **(A,B)** TT test; **(C,D)** PT test; **(E,F)** APTT test. Data points with clotting time ≥ 200 s are marked with arrows; the points corresponds non-clotting during the measurement limit of coagulometer.

This protocol of administration provided a continuous anticoagulant effect (**Figure 5**). In the case of aptamer RA-36, the anticoagulant effect increased with each injection at all concentrations. Thus, the protocol could be optimized further to provide steady-state conditions. Similar to the single bolus administration, repeated injections of aptamer RA-36 in 42 mg/kg dose had the same anticoagulant effect as repeated injections of bivalirudin in 0.75 mg/kg dose.

### Antithrombotic Efficiency of Aptamer RA-36 and Bivalirudin

**FIGURE 6:**
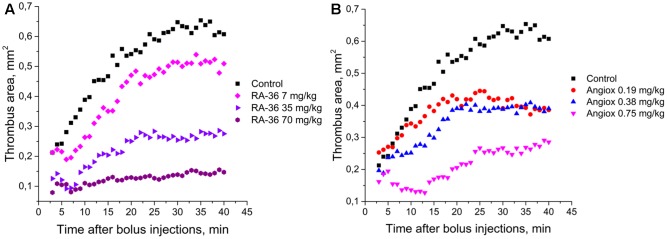
Antithrombotic activity in the alive mice model of induced thrombosis: **(A)** aptamer RA-36 (7, 35, and 70 mg/kg doses; 10 animals per dose), **(B)** bivalirudin (0.19, 0.38, and 0.75 mg/kg doses; 6, 10, and 9 animals per dose, respectively).

The mouse model of induced thrombosis was used to assess the antithrombotic efficiency of aptamer RA-36 and bivalirudin. Anticoagulants were bolus administered intravenously in the following doses: aptamer RA-36 – 7.0, 35, and 70 mg/kg that corresponds to 0.70, 3.5, and 7.0 μmole/kg doses; bivalirudin – 0.75, 0.38, and 0.19 mg/kg that corresponds to 0.34, 0.17, and 0.09 μmole/kg doses. Both anticoagulants decrease thrombus area in a dose-dependent manner (**Figure 6**). The standard deviations are rather large; the average absolute value of standard deviation is 0.14 mm^2^ (all the data are listed in the Supplementary Material). Statistical treatment using non-parametric statistics confirmed the data distributions for different doses of each anticoagulant to be significantly different.

Two parameters were used to compare the efficiencies of aptamer RA-36 and bivalirudin: initial and final sizes of thrombus (mean areas of thrombus during the first and the last 5 min of the experiments) (**Table 3**). The effect of the dose of 0.75 mg/kg of bivalirudin was approximately the same with the effect of the dose of 35 mg/kg of aptamer RA-36.

**Table 3:** Antithrombotic efficiencies of aptamer RA-36 and bivalirudin: mean area of thrombus at the beginning and at the end of the observation.

Group	Mean area of thrombus, mm^2^	
	The first 5 min	The last 5 min
Control	0.26 ± 0.09	0.62 ± 0.18
Bivalirudin 0.19 mg/kg	0.27 ± 0.09	0.38 ± 0.17
Bivalirudin 0.38 mg/kg	0.22 ± 0.10	0.39 ± 0.15
Bivalirudin 0.75 mg/kg	0.17 ± 0.07	0.28 ± 0.18
Aptamer RA-36 7.0 mg/kg	0.21 ± 0.07	0.5 ± 0.2
Aptamer RA-36 35 mg/kg	0.12 ± 0.12	0.3 ± 0.3
Aptamer RA-36 70 mg/kg	0.10 ± 0.09	0.15 ± 0.16

## Discussion

Aptamers are molecular recognition elements with high applicability in both fundamental science and medicine. According to the extended definition, two main classes of aptamers are peptides and nucleic acids – oligomers that are able to bind a target with high specificity and affinity. Inherently, peptides have a higher diversity due to a larger quantity of side chain variants providing good target binding. On the contrary, nucleic acid aptamers are generally more structured molecules that compensate low side chain diversity due to a binding entropy. Both directions are rapidly progressed providing valuable tools for diagnostics and treatment of diseases. Here, we have studied the efficiencies of two aptamers of a different nature *in vivo*, the oligopeptide and oligonucleotide, trying to solve the following problem. Is it possible to create a novel aptameric anticoagulant with both reduced bleeding and low immune side-effects?

Bivalirudin is a peptide that binds thrombin in a bivalent manner, targeting both the active site and the distal fibrinogen binding site (exosite I). RA-36 is DNA oligonucleotide that binds the thrombin distal substrate binding site only. Both compounds have inhibition constants in the nanomolar range: 2 nM for bivalirudin and 7 nM for aptamer RA-36. The compounds inhibits coagulation cascade in the same concentration range, although bivalirudin is more efficient inhibitor than RA-36 (Zavyalova et al., 2016b). The difference in a type of thrombin binding is seen in the sophisticated thrombin generation test. In blood plasma, bivalirudin prevents temporally any thrombin activity; then, the peptide is slowly hydrolyzed, fully restoring thrombin activity. On the contrary, RA-36 provides permanent inhibitory effect inhibiting those thrombin activities that are mediated with thrombin exosite I (Ustinov et al., 2016; Zavyalova et al., 2016b).

During the translation of the study from *in vitro* to *in vivo*, several additional factors play a vital role. The most prominent role belongs to pharmacokinetic profiles of the peptide aptamer and DNA one. Both inhibitors can be intravenously administered and their target compartment is the bloodstream. Non-modified DNA aptamers are known to be degraded and eliminated rapidly from the bloodstream. The elimination of the related aptamer HD1 *in vivo* from the bloodstream of cynomolgus monkeys and rats was described (Lee et al., 1995; Reyderman and Stavchansky, 1998). Pharmacokinetics of bivalirudin, peptide aptamer, was studied in humans (Chai et al., 2013), providing a solid background for a consideration of different substances in the class. Mean residence time in rats was only 2–3 min for aptamer HD1 (2.3–4.6 μmole/kg dose) versus 38 min for aptamer RA-36 (0.7 μmole/kg dose) and 27 min for bivalirudin in humans (0.34 μmole/kg dose). The time of distribution of the substance from the blood to tissues, parameter T_α_, was similar for both aptamers – 1.4 and 1.1 min for HD1 and RA-36, respectively, whereas bivalirudin gave a much higher value – 16 min (Zhang et al., 2012). The time of clearance from the tissues, parameter T_β_, differed drastically for two DNA aptamers – 5.5 and 23 min for HD1 and RA-36, respectively; the value for bivalirudin is similar to that of aptamer RA-36 – 35 min (Zhang et al., 2012). Thus, the nature of the DNA aptamer is determined by the elimination rate from the body, whereas the distribution from the bloodstream proceeded irrespective of the aptamer sequence. Peptide inhibitors have prolonged residence in the blood, providing a solid ground for dose diminishing.

Aptamer RA-36 has a distinct distribution between organs and tissues compared to other oligonucleotides. Here, 1–5 min after the intravenous administration aptamer HD1 is located mainly in the blood, kidneys, and liver (Reyderman and Stavchansky, 1998); a similar distribution is observed for 2′F- and 2′OMe-modified RNA aptamers (Healy et al., 2004). On the contrary, aptamer RA-36 was distributed in the blood, kidneys, vessels, and multiple tissues (**Figure 4**). Aptamer content in the liver was unexpectedly low. Thus, aptamer RA-36, having additional G-quadruplex compared to aptamer HD1, has an altered distribution profile. Bivalirudin is distributed from the blood to kidneys, skeletal muscle, skin, and bone with a smaller amount in the liver and spleen (European Medicines Agency library, 2005). Therefore, each aptamer has a unique distribution profile depending on the chemical structure of oligomer.

Several mechanisms of elimination from the blood are known for aptamer HD1: digestion with nucleases, elimination with kidneys, and distribution into tissues and organs. Aptamer half-life in the blood serum is 7 min due to nuclease digestion of the oligonucleotide (Shaw et al., 1995); this is significant but not critical. Aptamer RA-36 is twice larger and has a more sophisticated structure than HD1; then, its half-life in blood serum is expected to be equal or even larger than 7 min. The following reasons could explain the difference in estimated and experimental values.

The pharmacokinetic profile exerts a great effect on the anticoagulant activity of the substance. As it is shown in **Figures 2**, **3**, bivalirudin has apparently higher activity compared to aptamer RA-36. The anticoagulant effect of 0.35 μmole/kg dose of bivalirudin in the first experimental point (2 min after bolus intravenous administration) corresponds to 4.2 and 2.8 μmole/kg dose of aptamer RA-36 in rats and monkeys, correspondingly. The same tendency was observed in the mouse model of the induced thrombosis. These data are in accordance with the rate of drug excretion: bivalirudin clearance is 10-fold slower (3.2 ml/min/kg in humans; Robson et al., 2002) compared to aptamer RA-36 (33 ml/min/kg in rats, **Table 1**). Hence, the deceleration of aptamer excretion is an obvious way to decrease the effective doses.

To provide permanent anticoagulation, aptamer RA-36 was studied in multiple intravenous bolus dosing regimens. The aptamer was administered in rats every 15 min. The experiment design provides a rough model of the continuous administration of the aptamer. **Figure 5** shows that this regimen provides a plateau of the anticoagulant effect for 7 mg/kg aptamer dose; repeated higher doses provide positive increases in the curve, so a balance between elimination and bolus administration was not achieved. The more sophisticated selection of dosing could compensate for the excess of the aptamer in blood plasma.

## Conclusion

Two aptameric thrombin inhibitors of different nature were studied in terms of the combination of anticoagulant and antithrombotic activity *in vivo*, and pharmacokinetics. Both inhibitors provided high efficiency, highlighting the reach of the maximal activity for this class of drugs. On the contrary, the pharmacokinetic profiles differ drastically. At the current stage of aptameric drug development, it is the pharmacokinetics which mainly defines the therapeutic doses of the aptamers. A manipulation of the clearance rate of inhibitors is a straightforward way to improve existing anticoagulant aptamers. To reduce the renal clearance the molecular weight of aptamer RA-36 is to be increased for example using conjugation with high-molecular carrier; this modification is currently under studying.

## Author Contributions

EZ performed coagulation experiments with plasma samples and drafted the work. NS performed experiments with rats. AR performed experiments with mice. AT prepared aptamer samples for the experiments. IG performed experiments with monkeys. AG performed pharmacokinetic study. AK and GP designed the experiments, analyzed, and interpreted the results.

## Conflict of Interest Statement

The authors declare that the research was conducted in the absence of any commercial or financial relationships that could be construed as a potential conflict of interest.
