# Supplementary material for: The Evaluation of Pharmacodynamics and Pharmacokinetics of Anti-thrombin DNA Aptamer RA-36
Source: Front Pharmacol. 2017 Dec 14;8:922. doi: 10.3389/fphar.2017.00922 (PMC5735248; doi:10.3389/fphar.2017.00922)
Supplement: Supplementary file 1 [file Data_Sheet_1.docx]

Supplementary Material

The Evaluation of Pharmacodynamics and Pharmacokinetics

of Anti-thrombin DNA Aptamer RA-36

Elena Zavyalova^*^, Nadezhda Samoylenkova, Alexander Revishchin, Askar Turashev, Ilya Gordeychuk, Andrey Golovin, Alexey Kopylov, Galina Pavlova

*** Correspondence:**Elena Zavyalova
zlenka2006@gmail.com

Keywords: anticoagulant, blood coagulation, DNA aptamer, bivalirudin, inhibitor, preclinical trials, thrombin, pharmacokinetics

# Supplementary Tables

**Table S1.** Thrombin time of blood plasma samples drawn during 1 hour after the single injection of saline solution in rats.

| **Group #** | **Injected dose** | **Animal code** | **The time after drug injection, min** | | | | | | |
| --- | --- | --- | --- | --- | --- | --- | --- | --- | --- |
|  |  |  | **0** | **2** | **5** | **10** | **15** | **30** | **60** |
| 1 | 200 µl | 001 | 63.3 | 49.3 | 51.7 | 48.1 | 46.2 | 48.5 | 47.2 |
|  |  | 002 | 57.5 | 56.1 | 56.3 | 52.5 | 56.7 | 49.8 | 47.1 |
|  |  | 003 | 51.3 | 45.1 | 44.0 | 43.6 | 38.7 | 43.2 | 36.5 |
|  |  | 004 | 55.7 | 57.7 | 54.7 | 59.9 | 49.2 | 60.7 | 54.1 |
|  |  | 005 | 50.1 | 52.1 | 50.4 | 48.5 | 47.6 | 46.8 | 43.0 |
|  |  | 006 | 49.3 | 49.5 | 46.5 | 48.8 | 44.3 | 47.5 | 48.3 |
|  |  | 007 | 50.9 | 50.0 | 47.9 | 48.8 | 48.1 | 49.8 | 45.8 |
|  |  | 008 | 43.9 | 48.5 | 44.9 | 49.5 | 46.4 | 48.1 | 44.7 |
|  |  | 009 | 44.9 | 50.3 | 51.9 | 46.1 | 49.7 | 63.5 | 45.1 |
|  |  | 010 | 53.3 | 47.8 | 48.3 | 47.9 | 41.3 | 52.5 | 48.2 |
| ***Mean value*** | | | ***52*** | ***51*** | ***50*** | ***49*** | ***47*** | ***51*** | ***46*** |
| ***Geometrical mean value*** | | | ***52*** | ***51*** | ***50*** | ***49*** | ***47*** | ***51*** | ***46*** |
| ***Standard deviation*** | | | ***6*** | ***4*** | ***4*** | ***4*** | ***5*** | ***6*** | ***5*** |
| ***Coefficient of variation*** | | | ***11*** | ***8*** | ***8*** | ***9*** | ***11*** | ***12*** | ***10*** |
| ***Median*** | | | ***51*** | ***50*** | ***49*** | ***49*** | ***47*** | ***49*** | ***47*** |
| ***Lower 90% CI of Mean*** | | | ***49*** | ***49*** | ***48*** | ***47*** | ***44*** | ***48*** | ***44*** |
| ***Upper 90% CI of Mean*** | | | ***55*** | ***53*** | ***52*** | ***52*** | ***49*** | ***54*** | ***48*** |

**Table S2.** Thrombin time of blood plasma samples drawn during 1 hour after the single injection of Angiox® in rats.

| **Group #** | **Injected dose** | | | **Animal code** | | **The time after drug injection, min** | | | | | | | | | | | | |
| --- | --- | --- | --- | --- | --- | --- | --- | --- | --- | --- | --- | --- | --- | --- | --- | --- | --- | --- |
|  |  |  |  |  |  | **0** | | **2** | | **5** | | **10** | | **15** | | **30** | | **60** |
| 2 | 0.19 mg/kg | | | 011 | | 42.6 | | 200 | | 200 | | 114.1 | | 144.1 | | 121.7 | | 50.4 |
|  |  |  |  | 012 | | 47.3 | | 200 | | 200 | | 200 | | 200 | | 146.1 | | 93.7 |
|  |  |  |  | 013 | | 43.4 | | 200 | | 200 | | 200 | | 200 | | 134.9 | | 82.3 |
|  |  |  |  | 014 | | 51.5 | | 200 | | 200 | | 200 | | 200 | | 146.9 | | 59.7 |
|  |  |  |  | 015 | | 50.1 | | 200 | | 200 | | 200 | | 176.7 | | 121.3 | | 81.7 |
|  |  |  |  | 016 | | 52.7 | | 200 | | 200 | | 200 | | 200 | | 121.7 | | 70.9 |
|  |  |  |  | 017 | | 59.1 | | 200 | | 200 | | 200 | | 136.9 | | 53.6 | | 55.5 |
|  |  |  |  | 018 | | 63.5 | | 200 | | 200 | | 200 | | 152.9 | | 134.3 | | 60.3 |
|  |  |  |  | 019 | | 38.2 | | 200 | | 200 | | 200 | | 200 | | 77.3 | | 84.1 |
|  |  |  |  | 020 | | 44.1 | | 200 | | 200 | | 156.7 | | 128.9 | | 60.4 | | 50.4 |
| ***Mean value*** | | | | | | ***49*** | | ***200*** | | ***200*** | | ***190*** | | ***170*** | | ***110*** | | ***69*** |
| ***Geometrical mean value*** | | | | | | ***49*** | | ***200*** | | ***200*** | | ***180*** | | ***170*** | | ***110*** | | ***67*** |
| ***Standard deviation*** | | | | | | ***8*** | | ***-*** | | ***-*** | | ***30*** | | ***30*** | | ***30*** | | ***16*** |
| ***Coefficient of variation*** | | | | | | ***16*** | | ***-*** | | ***-*** | | ***16*** | | ***17*** | | ***30*** | | ***20*** |
| ***Median*** | | | | | | ***49*** | | ***200*** | | ***200*** | | ***200*** | | ***190*** | | ***120*** | | ***66*** |
| ***Lower 90% CI of Mean*** | | | | | | ***45*** | | ***-*** | | ***-*** | | ***170*** | | ***160*** | | ***94*** | | ***61*** |
| ***Upper 90% CI of Mean*** | | | | | | ***53*** | | ***-*** | | ***-*** | | ***200*** | | ***190*** | | ***130*** | | ***77*** |
| 3 | | 0.38 mg/kg | 021 | | 53.9 | | 200 | | 200 | | 200 | | 200 | | 127.3 | | 102.5 | |
|  |  |  | 022 | | 44.8 | | 200 | | 200 | | 200 | | 168.7 | | 200 | | 88.1 | |
|  |  |  | 023 | | 43.9 | | 200 | | 200 | | 200 | | 149.9 | | 135.6 | | 117.3 | |
|  |  |  | 024 | | 39.0 | | 200 | | 200 | | 200 | | 181.5 | | 159.0 | | 66.8 | |
|  |  |  | 025 | | 51.7 | | 200 | | 200 | | 200 | | 200 | | 200 | | 150.9 | |
|  |  |  | 026 | | 51.6 | | 200 | | 200 | | 200 | | 143.3 | | 94.9 | | 68.1 | |
|  |  |  | 027 | | 50.0 | | 200 | | 200 | | 200 | | 200 | | 200 | | 142.1 | |
|  |  |  | 028 | | 45.8 | | 200 | | 200 | | 200 | | 200 | | 152.5 | | 80.1 | |
|  |  |  | 029 | | 48.1 | | 200 | | 200 | | 200 | | 200 | | 200 | | 130.5 | |
|  |  |  | 030 | | 44.3 | | 200 | | 200 | | 200 | | 200 | | 200 | | 96.7 | |
| ***Mean value*** | | | | | ***47*** | | ***200*** | | ***200*** | | ***200*** | | ***180*** | | ***170*** | | ***100*** | |
| ***Geometrical mean value*** | | | | | ***47*** | | ***200*** | | ***200*** | | ***200*** | | ***180*** | | ***160*** | | ***100*** | |
| ***Standard deviation*** | | | | | ***5*** | | ***-*** | | ***-*** | | ***-*** | | ***20*** | | ***40*** | | ***30*** | |
| ***Coefficient of variation*** | | | | | ***10*** | | ***-*** | | ***-*** | | ***-*** | | ***12*** | | ***20*** | | ***30*** | |
| ***Median*** | | | | | ***47*** | | ***200*** | | ***200*** | | ***200*** | | ***200*** | | ***180*** | | ***100*** | |
| ***Lower 90% CI of Mean*** | | | | | ***45*** | | ***-*** | | ***-*** | | ***-*** | | ***170*** | | ***150*** | | ***90*** | |
| ***Upper 90% CI of Mean*** | | | | | ***50*** | | ***-*** | | ***-*** | | ***-*** | | ***200*** | | ***190*** | | ***120*** | |
| 4 | | 0.75 mg/kg | 031 | | 51.1 | | 200 | | 200 | | 200 | | 200 | | 200 | | 52.0 | |
|  |  |  | 032 | | 48.4 | | 200 | | 200 | | 200 | | 200 | | 200 | | 46.3 | |
|  |  |  | 033 | | 40.5 | | 200 | | 200 | | 200 | | 200 | | 111.3 | | 40.0 | |
|  |  |  | 034 | | 39.4 | | 200 | | 200 | | 200 | | 200 | | 200 | | 55.1 | |
|  |  |  | 035 | | 52.1 | | 200 | | 200 | | 200 | | 200 | | 200 | | 48.7 | |
|  |  |  | 036 | | 42.6 | | 200 | | 200 | | 200 | | 200 | | 200 | | 53.0 | |
|  |  |  | 037 | | 47.9 | | 200 | | 200 | | 200 | | 175.3 | | 160.7 | | 48.5 | |
|  |  |  | 038 | | 42.7 | | 200 | | 200 | | 200 | | 200 | | 200 | | 52.3 | |
|  |  |  | 039 | | 40.7 | | 200 | | 200 | | 200 | | 174.5 | | 68.3 | | 47.1 | |
|  |  |  | 040 | | 42.2 | | 200 | | 200 | | 200 | | 200 | | 116.3 | | 118.7 | |
| ***Mean value*** | | | | | ***45*** | | ***200*** | | ***200*** | | ***200*** | | ***195*** | | ***170*** | | ***60*** | |
| ***Geometrical mean value*** | | | | | ***45*** | | ***200*** | | ***200*** | | ***200*** | | ***195*** | | ***160*** | | ***50*** | |
| ***Standard deviation*** | | | | | ***5*** | | ***-*** | | ***-*** | | ***-*** | | ***11*** | | ***50*** | | ***20*** | |
| ***Coefficient of variation*** | | | | | ***10*** | | ***-*** | | ***-*** | | ***-*** | | ***5*** | | ***30*** | | ***40*** | |
| ***Median*** | | | | | ***43*** | | ***200*** | | ***200*** | | ***200*** | | ***200*** | | ***200*** | | ***50*** | |
| ***Lower 90% CI of Mean*** | | | | | ***42*** | | ***-*** | | ***-*** | | ***-*** | | ***190*** | | ***140*** | | ***45*** | |
| ***Upper 90% CI of Mean*** | | | | | ***47*** | | ***-*** | | ***-*** | | ***-*** | | ***200*** | | ***190*** | | ***68*** | |

**Table S3.** Thrombin time of blood plasma samples drawn during 1 hour after the single injection of Thrombiveb® in rats.

| **Group #** | **Injected dose** | **Animal code** | **The time after drug injection, min** | | | | | | | | | | | |
| --- | --- | --- | --- | --- | --- | --- | --- | --- | --- | --- | --- | --- | --- | --- |
|  |  |  | **0** | | **2** | | **5** | | **10** | | **15** | | **30** | **60** |
| 5 | 7.0 mg/kg | 041 | 45.5 | | 200 | | 83.9 | | 77.7 | | 55.5 | | 43.1 | 41.1 |
|  |  | 042 | 49.5 | | 200 | | 147.5 | | 72.9 | | 62.2 | | 49.2 | 45.0 |
|  |  | 043 | 55.9 | | 200 | | 200 | | 90.3 | | 63.7 | | 62.9 | 57.9 |
|  |  | 044 | 55.1 | | 200 | | 164.5 | | 97.3 | | 75.1 | | 57.7 | 50.3 |
|  |  | 045 | 64.9 | | 200 | | 180.1 | | 108.3 | | 92.7 | | 71.1 | 57.1 |
|  |  | 046 | 50.8 | | 200 | | 141.3 | | 87.1 | | 98.7 | | 65.9 | 58.7 |
|  |  | 047 | 48.8 | | 200 | | 145.5 | | 62.9 | | 64.1 | | 56.7 | 52.6 |
|  |  | 048 | 48.9 | | 200 | | 161.3 | | 67.3 | | 73.3 | | 51.5 | 45.6 |
|  |  | 049 | 56.7 | | 200 | | 125.1 | | 101.7 | | 78.1 | | 61.1 | 50.7 |
|  |  | 050 | 50.9 | | 200 | | 134.1 | | 94.5 | | 72.3 | | 54.9 | 48.3 |
| ***Mean value*** | | | ***53*** | | ***200*** | | ***150*** | | ***86*** | | ***74*** | | ***57*** | ***51*** |
| ***Geometrical mean value*** | | | ***52*** | | ***200*** | | ***140*** | | ***85*** | | ***73*** | | ***57*** | ***50*** |
| ***Standard deviation*** | | | ***6*** | | ***-*** | | ***30*** | | ***15*** | | ***14*** | | ***8*** | ***6*** |
| ***Coefficient of variation*** | | | ***11*** | | ***-*** | | ***20*** | | ***18*** | | ***19*** | | ***14*** | ***12*** |
| ***Median*** | | | ***51*** | | ***200*** | | ***150*** | | ***89*** | | ***73*** | | ***57*** | ***51*** |
| ***Lower 90% CI of Mean*** | | | ***50*** | | ***-*** | | ***130*** | | ***78*** | | ***67*** | | ***53*** | ***48*** |
| ***Upper 90% CI of Mean*** | | | ***56*** | | ***-*** | | ***160*** | | ***94*** | | ***81*** | | ***62*** | ***54*** |
| 6 | 21 mg/kg | 051 | 50.6 | | 200 | | 174.7 | | 101.3 | | 73.3 | | 82.5 | 59.7 |
|  |  | 052 | 51.1 | | 200 | | 200 | | 200 | | 116.9 | | 87.1 | 89.3 |
|  |  | 053 | 45.8 | | 200 | | 169.3 | | 153.7 | | 121.5 | | 86.7 | 92.5 |
|  |  | 054 | 43.6 | | 200 | | 144.6 | | 80.5 | | 63.3 | | 47.4 | 40.6 |
|  |  | 055 | 44.9 | | 200 | | 200 | | 135.9 | | 120.7 | | 108.5 | 122.9 |
|  |  | 056 | 45.9 | | 200 | | 200 | | 154.7 | | 140.5 | | 126.1 | 133.9 |
|  |  | 057 | 48.7 | | 200 | | 200 | | 143.9 | | 139.1 | | 118.1 | 93.7 |
|  |  | 058 | 46.3 | | 200 | | 160.1 | | 157.5 | | 145.1 | | 84.5 | 168.5 |
|  |  | 059 | 54.5 | | 200 | | 200 | | 200 | | 138.1 | | 135.3 | 105.1 |
|  |  | 060 | 47.9 | | 200 | | 200 | | 159.5 | | 132.9 | | 121.9 | 77.5 |
| ***Mean value*** | | | ***48*** | | ***200*** | | ***190*** | | ***150*** | | ***120*** | | ***100*** | ***100*** |
| ***Geometrical mean value*** | | | ***48*** | | ***200*** | | ***190*** | | ***140*** | | ***120*** | | ***100*** | ***100*** |
| ***Standard deviation*** | | | ***3*** | | ***-*** | | ***20*** | | ***40*** | | ***30*** | | ***30*** | ***40*** |
| ***Coefficient of variation*** | | | ***7*** | | ***-*** | | ***11*** | | ***30*** | | ***20*** | | ***30*** | ***40*** |
| ***Median*** | | | ***47*** | | ***200*** | | ***200*** | | ***150*** | | ***130*** | | ***100*** | ***90*** |
| ***Lower 90% CI of Mean*** | | | ***46*** | | ***-*** | | ***180*** | | ***130*** | | ***100*** | | ***90*** | ***80*** |
| ***Upper 90% CI of Mean*** | | | ***50*** | | ***-*** | | ***200*** | | ***170*** | | ***130*** | | ***110*** | ***120*** |
| 7 | 42 mg/kg | 061 | 44.2 | | 200 | | 200 | | 99.9 | | 132.5 | | 95.7 | 82.5 |
|  |  | 062 | 50.4 | | 200 | | 200 | | 134.1 | | 121.3 | | 90.5 | 87.9 |
|  |  | 063 | 45.8 | | 200 | | 200 | | 142.3 | | 140.5 | | 114.1 | 117.7 |
|  |  | 064 | 47.5 | | 200 | | 200 | | 164.5 | | 147.7 | | 152.9 | 116.9 |
|  |  | 065 | 44.4 | | 200 | | 200 | | 200 | | 145.1 | | 118.5 | 99.7 |
|  |  | 066 | 48.7 | | 200 | | 200 | | 136.1 | | 89.3 | | 109.7 | 93.3 |
|  |  | 067 | 44.4 | | 200 | | 200 | | 200 | | 142.1 | | 148.1 | 88.9 |
|  |  | 068 | 45.9 | | 200 | | 200 | | 144.7 | | 116.5 | | 104.9 | 84.1 |
|  |  | 069 | 45.4 | | 200 | | 200 | | 139.1 | | 144.1 | | 116.5 | 143.9 |
|  |  | 070 | 44.3 | | 200 | | 200 | | 135.1 | | 106.1 | | 86.1 | 200 |
| ***Mean value*** | | | ***46*** | ***200*** | | ***200*** | | ***150*** | | ***130*** | | ***110*** | | ***110*** |
| ***Geometrical mean value*** | | | ***46*** | ***200*** | | ***200*** | | ***150*** | | ***130*** | | ***110*** | | ***100*** |
| ***Standard deviation*** | | | ***2*** | ***-*** | | ***-*** | | ***30*** | | ***20*** | | ***20*** | | ***40*** |
| ***Coefficient of variation*** | | | ***5*** | ***-*** | | ***-*** | | ***20*** | | ***15*** | | ***20*** | | ***30*** |
| ***Median*** | | | ***46*** | ***200*** | | ***200*** | | ***140*** | | ***140*** | | ***110*** | | ***100*** |
| ***Lower 90% CI of Mean*** | | | ***45*** | ***-*** | | ***-*** | | ***130*** | | ***120*** | | ***100*** | | ***90*** |
| ***Upper 90% CI of Mean*** | | | ***47*** | ***-*** | | ***-*** | | ***170*** | | ***140*** | | ***130*** | | ***130*** |

**Table S4.** Prothrombin time of blood plasma samples drawn during 1 hour after the single injection of saline solution in rats.

| **Group #** | **Injected dose** | **Animal code** | **The time after drug injection, min** | | | | | | |
| --- | --- | --- | --- | --- | --- | --- | --- | --- | --- |
|  |  |  | **0** | **2** | **5** | **10** | **15** | **30** | **60** |
| 1 | 200 µl | 001 | 11.4 | 10.7 | 11.2 | 11.2 | 11.2 | 11.3 | 11.1 |
|  |  | 002 | 11.4 | 11.3 | 12.0 | 12.2 | 10.8 | 11.3 | 10.4 |
|  |  | 003 | 11.5 | 10.6 | 10.7 | 10.9 | 9.4 | 10.7 | 9.5 |
|  |  | 004 | 11.8 | 12.3 | 12.2 | 11.5 | 9.9 | 11.5 | 11.5 |
|  |  | 005 | 10.8 | 10.6 | 10.7 | 10.2 | 8.7 | 10.3 | 10.1 |
|  |  | 006 | 11.6 | 11.4 | 11.3 | 11.4 | 11.2 | 11.2 | 11.8 |
|  |  | 007 | 9.8 | 9.9 | 9.9 | 10.1 | 9.3 | 9.9 | 10.2 |
|  |  | 008 | 11.2 | 10.3 | 11.4 | 11.8 | 10.4 | 11.3 | 9.8 |
|  |  | 009 | 10.1 | 11.3 | 10.4 | 10.3 | 10.8 | 10.6 | 10.8 |
|  |  | 010 | 9.6 | 10.3 | 10.4 | 10 | 9.4 | 10.0 | 10.1 |
| ***Mean value*** | | | ***10.9*** | ***10.9*** | ***11.0*** | ***11.0*** | ***10.1*** | ***10.8*** | ***10.5*** |
| ***Geometrical mean value*** | | | ***10.9*** | ***10.8*** | ***11.0*** | ***10.9*** | ***10.1*** | ***10.8*** | ***10.5*** |
| ***Standard deviation*** | | | ***0.8*** | ***0.7*** | ***0.7*** | ***0.8*** | ***0.9*** | ***0.6*** | ***0.7*** |
| ***Coefficient of variation*** | | | ***7.0*** | ***6.5*** | ***6.7*** | ***7.1*** | ***8.8*** | ***5.5*** | ***7.1*** |
| ***Median*** | | | ***11.3*** | ***10.7*** | ***11.0*** | ***11.1*** | ***10.2*** | ***11.0*** | ***10.3*** |
| ***Lower 90% CI of Mean*** | | | ***10.5*** | ***10.5*** | ***10.6*** | ***10.6*** | ***9.6*** | ***10.5*** | ***10.1*** |
| ***Upper 90% CI of Mean*** | | | ***11.3*** | ***11.2*** | ***11.4*** | ***11.4*** | ***10.6*** | ***11.1*** | ***10.9*** |

**Table S5.** Prothrombin time of blood plasma samples drawn during 1 hour after the single injection of Angiox® in rats.

| **Group #** | **Injected dose** | **Animal code** | **The time after drug injection, min** | | | | | | |
| --- | --- | --- | --- | --- | --- | --- | --- | --- | --- |
|  |  |  | **0** | **2** | **5** | **10** | **15** | **30** | **60** |
| 2 | 0.19 mg/kg | 011 | 10.5 | 74.3 | 27.0 | 31.9 | 15.9 | 13.0 | 11.8 |
|  |  | 012 | 9.7 | 67.0 | 37.5 | 21.1 | 14.9 | 11.7 | 10.9 |
|  |  | 013 | 11.0 | 73.3 | 37.7 | 22.9 | 15.4 | 12.6 | 11.4 |
|  |  | 014 | 11.0 | 81.8 | 59.7 | 36.8 | 18.3 | 11.4 | 11.8 |
|  |  | 015 | 10.3 | 58.4 | 29.2 | 19.3 | 13.1 | 11.3 | 9.1 |
|  |  | 016 | 10.1 | 72.4 | 43.3 | 25.8 | 20.7 | 14.1 | 10.3 |
|  |  | 017 | 10.2 | 75.6 | 45.5 | 28.6 | 15.2 | 9.5 | 10.6 |
|  |  | 018 | 9.7 | 71.0 | 43.6 | 27.5 | 15.5 | 12.4 | 11.8 |
|  |  | 019 | 9.8 | 75.7 | 35.2 | 19.5 | 13.7 | 11.7 | 10.6 |
|  |  | 020 | 10.3 | 76.9 | 45.8 | 25.6 | 13.5 | 11.9 | 11.1 |
| ***Mean value*** | | | ***10.3*** | ***73*** | ***41*** | ***26*** | ***16*** | ***12.0*** | ***10.9*** |
| ***Geometrical mean value*** | | | ***10.3*** | ***72*** | ***40*** | ***25*** | ***16*** | ***11.9*** | ***10.9*** |
| ***Standard deviation*** | | | ***0.5*** | ***6*** | ***9*** | ***6*** | ***2*** | ***1.2*** | ***0.8*** |
| ***Coefficient of variation*** | | | ***4.6*** | ***9*** | ***20*** | ***22*** | ***15*** | ***10.1*** | ***7.8*** |
| ***Median*** | | | ***10.3*** | ***74*** | ***41*** | ***26*** | ***15*** | ***11.8*** | ***11.0*** |
| ***Lower 90% CI of Mean*** | | | ***10.0*** | ***69*** | ***36*** | ***23*** | ***14*** | ***11.3*** | ***10.5*** |
| ***Upper 90% CI of Mean*** | | | ***10.5*** | ***76*** | ***45*** | ***29*** | ***17*** | ***12.6*** | ***11.4*** |
| 3 | 0.38 mg/kg | 021 | 12.4 | 200 | 83.4 | 76.0 | 34.3 | 17.0 | 14.0 |
|  |  | 022 | 12.3 | 200 | 200 | 71.7 | 24.6 | 16.6 | 12.3 |
|  |  | 023 | 10.2 | 200 | 79.7 | 59.1 | 25.6 | 21.7 | 14.0 |
|  |  | 024 | 12.3 | 200 | 79.9 | 70.1 | 23.2 | 13.9 | 13.9 |
|  |  | 025 | 11.4 | 200 | 76.4 | 70.8 | 38.8 | 15.7 | 13.2 |
|  |  | 026 | 10.6 | 84.9 | 63.4 | 38.5 | 17.1 | 13.4 | 11.3 |
|  |  | 027 | 10.6 | 200 | 73.7 | 58.4 | 21.6 | 14.1 | 11.4 |
|  |  | 028 | 10.3 | 200 | 67.8 | 47.0 | 18.6 | 13.7 | 11.4 |
|  |  | 029 | 11.5 | 200 | 75.2 | 55.4 | 19.5 | 14.2 | 13.2 |
|  |  | 030 | 11.1 | 200 | 200 | 78.7 | 47.8 | 20.0 | 11.8 |
| ***Mean value*** | | | ***11.3*** | ***190*** | ***100*** | ***63*** | ***27*** | ***16*** | ***12.7*** |
| ***Geometrical mean value*** | | | ***11.2*** | ***180*** | ***90*** | ***61*** | ***26*** | ***16*** | ***12.6*** |
| ***Standard deviation*** | | | ***0.8*** | ***40*** | ***50*** | ***13*** | ***10*** | ***3*** | ***1.1*** |
| ***Coefficient of variation*** | | | ***7.5*** | ***20*** | ***50*** | ***21*** | ***37*** | ***18*** | ***9.0*** |
| ***Median*** | | | ***11.3*** | ***200*** | ***80*** | ***65*** | ***24*** | ***15*** | ***12.8*** |
| ***Lower 90% CI of Mean*** | | | ***10.8*** | ***170*** | ***70*** | ***56*** | ***22*** | ***15*** | ***12.1*** |
| ***Upper 90% CI of Mean*** | | | ***11.7*** | ***210*** | ***130*** | ***69*** | ***32*** | ***18*** | ***13.2*** |
| 4 | 0.75 mg/kg | 031 | 9.9 | 200 | 200 | 200 | 74.3 | 28.0 | 10.0 |
|  |  | 032 | 10.7 | 200 | 86.1 | 73.8 | 34.1 | 14.4 | 10.6 |
|  |  | 033 | 10.7 | 200 | 200 | 200 | 200 | 200 | 10.9 |
|  |  | 034 | 11.0 | 200 | 200 | 86.3 | 53.9 | 24.6 | 13.4 |
|  |  | 035 | 10.4 | 200 | 81.8 | 70.2 | 20.6 | 13.0 | 12.9 |
|  |  | 036 | 10.9 | 200 | 200 | 200 | 200 | 200 | 13.5 |
|  |  | 037 | 10.3 | 200 | 200 | 86.2 | 45.9 | 14.6 | 13.3 |
|  |  | 038 | 11.1 | 200 | 200 | 200 | 200 | 32.8 | 12.7 |
|  |  | 039 | 10.2 | 200 | 200 | 86.3 | 46.7 | 15.8 | 12.9 |
|  |  | 040 | 10.9 | 200 | 200 | 79.2 | 200 | 17.0 | 14.3 |
| ***Mean value*** | | | ***10.6*** | ***200*** | ***180*** | ***130*** | ***110*** | ***60*** | ***12.5*** |
| ***Geometrical mean value*** | | | ***10.6*** | ***200*** | ***170*** | ***120*** | ***80*** | ***30*** | ***12.4*** |
| ***Standard deviation*** | | | ***0.4*** | ***-*** | ***50*** | ***60*** | ***80*** | ***80*** | ***1.4*** |
| ***Coefficient of variation*** | | | ***3.7*** | ***-*** | ***30*** | ***50*** | ***80*** | ***140*** | ***11.5*** |
| ***Median*** | | | ***10.7*** | ***200*** | ***200*** | ***90*** | ***60*** | ***20*** | ***12.9*** |
| ***Lower 90% CI of Mean*** | | | ***10.4*** | ***-*** | ***150*** | ***100*** | ***70*** | ***20*** | ***11.7*** |
| ***Upper 90% CI of Mean*** | | | ***10.8*** | ***-*** | ***200*** | ***160*** | ***150*** | ***100*** | ***13.2*** |

**Table S6.** Prothrombin time of blood plasma samples drawn during 1 hour after the single injection of Thrombiveb® in rats.

| **Group #** | **Injected dose** | **Animal code** | **The time after drug injection, min** | | | | | | |
| --- | --- | --- | --- | --- | --- | --- | --- | --- | --- |
|  |  |  | **0** | **2** | **5** | **10** | **15** | **30** | **60** |
| 5 | 7.0 mg/kg | 041 | 9.8 | 20.0 | 13.8 | 10.2 | 10.0 | 9.8 | 10.1 |
|  |  | 042 | 11.4 | 21.2 | 12.3 | 11.4 | 12.1 | 12.5 | 13.1 |
|  |  | 043 | 10.0 | 23.7 | 14.7 | 12.9 | 11.6 | 11.5 | 11.8 |
|  |  | 044 | 11.4 | 23.7 | 14.4 | 11.7 | 11.7 | 11.7 | 11.3 |
|  |  | 045 | 10.6 | 21.7 | 14.9 | 11.4 | 10.4 | 10.2 | 10.2 |
|  |  | 046 | 10.3 | 21.9 | 13.1 | 11.8 | 11.2 | 12.6 | 12.4 |
|  |  | 047 | 10.2 | 24.8 | 12.6 | 10.9 | 11.4 | 11.0 | 11.0 |
|  |  | 048 | 8.9 | 18.4 | 14.4 | 9.7 | 9.5 | 9.8 | 10.2 |
|  |  | 049 | 11.6 | 26.2 | 11.4 | 12.5 | 12.5 | 11.2 | 10.4 |
|  |  | 050 | 11.3 | 21.9 | 13.3 | 10.5 | 10.8 | 10.2 | 10.1 |
| ***Mean value*** | | | ***10.6*** | ***22*** | ***13.5*** | ***11.3*** | ***11.1*** | ***11.1*** | ***11.1*** |
| ***Geometrical mean value*** | | | ***10.5*** | ***22*** | ***13.4*** | ***11.3*** | ***11.1*** | ***11.0*** | ***11.0*** |
| ***Standard deviation*** | | | ***0.9*** | ***2*** | ***1.2*** | ***1.0*** | ***0.9*** | ***1.0*** | ***1.1*** |
| ***Coefficient of variation*** | | | ***8.3*** | ***10*** | ***8.5*** | ***8.8*** | ***8.5*** | ***9.4*** | ***9.7*** |
| ***Median*** | | | ***10.5*** | ***22*** | ***13.6*** | ***11.4*** | ***11.3*** | ***11.1*** | ***10.7*** |
| ***Lower 90% CI of Mean*** | | | ***10.1*** | ***21*** | ***12.9*** | ***10.8*** | ***10.6*** | ***10.5*** | ***10.5*** |
| ***Upper 90% CI of Mean*** | | | ***11.0*** | ***24*** | ***14.1*** | ***11.8*** | ***11.6*** | ***11.6*** | ***11.6*** |
| 6 | 21 mg/kg | 051 | 9.3 | 30.3 | 18.7 | 9.4 | 10.3 | 9.9 | 10.2 |
|  |  | 052 | 10.4 | 34.5 | 21.9 | 14.2 | 10.6 | 11.1 | 10.9 |
|  |  | 053 | 9.9 | 31.3 | 16.8 | 11.3 | 11.0 | 10.5 | 10.4 |
|  |  | 054 | 12.4 | 37.9 | 23.1 | 11.3 | 10.9 | 10.9 | 11.2 |
|  |  | 055 | 11.3 | 45.0 | 31.3 | 12.1 | 10.9 | 10.8 | 10.8 |
|  |  | 056 | 10.3 | 34.6 | 18.2 | 11.5 | 11.3 | 10.8 | 10.3 |
|  |  | 057 | 10.2 | 39.0 | 25.6 | 11.3 | 11.4 | 10.3 | 9.0 |
|  |  | 058 | 11.1 | 31.6 | 13.8 | 11.8 | 11.6 | 11.3 | 11.4 |
|  |  | 059 | 10.9 | 35.0 | 22.6 | 12.0 | 10.7 | 11.1 | 10.6 |
|  |  | 060 | 10.3 | 32.5 | 21.8 | 11.4 | 11.3 | 10.4 | 10.8 |
| ***Mean value*** | | | ***10.6*** | ***35*** | ***21*** | ***11.6*** | ***11.0*** | ***10.7*** | ***10.6*** |
| ***Geometrical mean value*** | | | ***10.6*** | ***35*** | ***21*** | ***11.6*** | ***11.0*** | ***10.7*** | ***10.5*** |
| ***Standard deviation*** | | | ***0.9*** | ***4*** | ***5*** | ***1.2*** | ***0.4*** | ***0.4*** | ***0.7*** |
| ***Coefficient of variation*** | | | ***8.1*** | ***13*** | ***23*** | ***10.1*** | ***3.7*** | ***4.0*** | ***6.3*** |
| ***Median*** | | | ***10.4*** | ***35*** | ***22*** | ***11.5*** | ***11.0*** | ***10.8*** | ***10.7*** |
| ***Lower 90% CI of Mean*** | | | ***10.2*** | ***33*** | ***19*** | ***11.0*** | ***10.8*** | ***10.5*** | ***10.2*** |
| ***Upper 90% CI of Mean*** | | | ***11.1*** | ***38*** | ***24*** | ***12.2*** | ***11.2*** | ***10.9*** | ***10.9*** |
| 7 | 42 mg/kg | 061 | 10.6 | 42.9 | 24.2 | 16.1 | 11.0 | 10.9 | 10.3 |
|  |  | 062 | 9.8 | 34.4 | 22.9 | 11.0 | 10.2 | 10.3 | 10.1 |
|  |  | 063 | 10.1 | 47.0 | 33.2 | 13.4 | 11.5 | 11.5 | 11.4 |
|  |  | 064 | 9.6 | 35.4 | 24.5 | 11.4 | 10.0 | 10.0 | 9.2 |
|  |  | 065 | 10.5 | 44.3 | 40.0 | 21.3 | 13.1 | 11.3 | 11.0 |
|  |  | 066 | 10.4 | 37.9 | 24.1 | 11.2 | 10.8 | 10.3 | 10.0 |
|  |  | 067 | 10.5 | 43.2 | 33.3 | 13.1 | 11.7 | 11.1 | 10.3 |
|  |  | 068 | 10.5 | 42.2 | 28.0 | 11.8 | 11.3 | 10.8 | 10.6 |
|  |  | 069 | 10.3 | 40.0 | 23.1 | 11.6 | 11.2 | 10.8 | 10.7 |
|  |  | 070 | 10.4 | 38.1 | 18.1 | 10.5 | 11.1 | 10.6 | 11.3 |
| ***Mean value*** | | | ***10.3*** | ***41*** | ***27*** | ***13*** | ***11.2*** | ***10.8*** | ***10.5*** |
| ***Geometrical mean value*** | | | ***10.3*** | ***40*** | ***27*** | ***13*** | ***11.2*** | ***10.8*** | ***10.5*** |
| ***Standard deviation*** | | | ***0.3*** | ***4*** | ***7*** | ***3*** | ***0.9*** | ***0.5*** | ***0.7*** |
| ***Coefficient of variation*** | | | ***3.2*** | ***10*** | ***24*** | ***25*** | ***7.7*** | ***4.4*** | ***6.3*** |
| ***Median*** | | | ***10.4*** | ***41*** | ***24*** | ***12*** | ***11.2*** | ***10.8*** | ***10.5*** |
| ***Lower 90% CI of Mean*** | | | ***10.1*** | ***38*** | ***24*** | ***11*** | ***10.7*** | ***10.5*** | ***10.1*** |
| ***Upper 90% CI of Mean*** | | | ***10.4*** | ***43*** | ***31*** | ***15*** | ***11.6*** | ***11.0*** | ***10.8*** |

**Table S7.** APTT of blood plasma samples drawn during 1 hour after the single injection of saline solution in rats.

| **Group #** | **Injected dose** | **Animal code** | **The time after drug injection, min** | | | | | | |
| --- | --- | --- | --- | --- | --- | --- | --- | --- | --- |
|  |  |  | **0** | **2** | **5** | **10** | **15** | **30** | **60** |
| 1 | 200 µl | 001 | 23.4 | 21.9 | 23.0 | 24.3 | 22.8 | 22.3 | 22.1 |
|  |  | 002 | 22.3 | 23.4 | 20.8 | 21.6 | 22.7 | 20.6 | 22.4 |
|  |  | 003 | 21.2 | 20.9 | 21.7 | 19.8 | 25.2 | 20.4 | 18.9 |
|  |  | 004 | 18.2 | 18.2 | 18.4 | 18.0 | 18.7 | 19.5 | 19.9 |
|  |  | 005 | 19.2 | 19.7 | 20.2 | 21.1 | 20.3 | 19.6 | 20.6 |
|  |  | 006 | 19.8 | 20.2 | 20.6 | 22.0 | 21.8 | 21.9 | 23.7 |
|  |  | 007 | 20.8 | 20.8 | 19.2 | 18.9 | 18.8 | 20.7 | 20.0 |
|  |  | 008 | 21.3 | 22.0 | 27.8 | 23.6 | 21.6 | 22.8 | 20.4 |
|  |  | 009 | 21.1 | 21.4 | 22.6 | 21.7 | 22.3 | 20.0 | 22.4 |
|  |  | 010 | 21.3 | 19.6 | 20.5 | 21.6 | 19.0 | 19.6 | 19.2 |
| ***Mean value*** | | | ***20.9*** | ***20.8*** | ***22*** | ***21.3*** | ***21*** | ***20.7*** | ***21.0*** |
| ***Geometrical mean value*** | | | ***20.8*** | ***20.8*** | ***21*** | ***21.2*** | ***21*** | ***20.7*** | ***20.9*** |
| ***Standard deviation*** | | | ***1.5*** | ***1.5*** | ***3*** | ***1.9*** | ***2*** | ***1.2*** | ***1.6*** |
| ***Coefficient of variation*** | | | ***7.2*** | ***7.1*** | ***12*** | ***9.1*** | ***10*** | ***5.8*** | ***7.6*** |
| ***Median*** | | | ***21.2*** | ***20.9*** | ***21*** | ***21.6*** | ***22*** | ***20.5*** | ***20.5*** |
| ***Lower 90% CI of Mean*** | | | ***20.1*** | ***20.0*** | ***20*** | ***20.2*** | ***20*** | ***20.1*** | ***20.1*** |
| ***Upper 90% CI of Mean*** | | | ***21.6*** | ***21.6*** | ***23*** | ***22.3*** | ***22*** | ***21.4*** | ***21.8*** |

**Table S8.** APTT of blood plasma samples drawn during 1 hour after the single injection of Angiox® in rats.

| **Group #** | **Injected dose** | **Animal code** | **The time after drug injection, min** | | | | | | |
| --- | --- | --- | --- | --- | --- | --- | --- | --- | --- |
|  |  |  | **0** | **2** | **5** | **10** | **15** | **30** | **60** |
| 2 | 0.19 mg/kg | 011 | 19.1 | 110.4 | 68.3 | 35.6 | 51.2 | 33.8 | 31.1 |
|  |  | 012 | 19.2 | 104.3 | 65.5 | 51.0 | 38.5 | 27.4 | 21.0 |
|  |  | 013 | 19.0 | 108.2 | 81.8 | 60.4 | 43.7 | 29.0 | 21.9 |
|  |  | 014 | 26.1 | 147.4 | 96.6 | 76.8 | 54.0 | 46.0 | 30.6 |
|  |  | 015 | 18.6 | 89.3 | 59.1 | 48.1 | 37.9 | 26.5 | 21.5 |
|  |  | 016 | 22.8 | 110.0 | 72.5 | 55.2 | 44.4 | 25.4 | 24.4 |
|  |  | 017 | 17.9 | 150.7 | 107.8 | 57.5 | 39.8 | 25.9 | 19.3 |
|  |  | 018 | 22.8 | 111.1 | 88.6 | 61.6 | 39.5 | 30.8 | 23.3 |
|  |  | 019 | 20.2 | 135.5 | 87.3 | 58.2 | 45.0 | 32.1 | 29.6 |
|  |  | 020 | 20.9 | 168.7 | 82.7 | 67.3 | 43.0 | 36.0 | 29.6 |
| ***Mean value*** | | | ***21*** | ***120*** | ***81*** | ***57*** | ***44*** | ***31*** | ***25*** |
| ***Geometrical mean value*** | | | ***21*** | ***120*** | ***80*** | ***56*** | ***43*** | ***31*** | ***25*** |
| ***Standard deviation*** | | | ***3*** | ***30*** | ***15*** | ***11*** | ***5*** | ***6*** | ***5*** |
| ***Coefficient of variation*** | | | ***12*** | ***20*** | ***19*** | ***19*** | ***12*** | ***20*** | ***18*** |
| ***Median*** | | | ***20*** | ***110*** | ***82*** | ***58*** | ***43*** | ***30*** | ***24*** |
| ***Lower 90% CI of Mean*** | | | ***19*** | ***110*** | ***73*** | ***51*** | ***41*** | ***28*** | ***23*** |
| ***Upper 90% CI of Mean*** | | | ***22*** | ***140*** | ***89*** | ***63*** | ***47*** | ***35*** | ***28*** |
| 3 | 0.38 mg/kg | 021 | 16.9 | 170.3 | 124.3 | 104.0 | 68.2 | 46.3 | 34.0 |
|  |  | 022 | 23.5 | 168.1 | 150.5 | 129.6 | 89.5 | 76.2 | 46.1 |
|  |  | 023 | 25.7 | 165.9 | 156.2 | 102.7 | 66.5 | 59.4 | 39.1 |
|  |  | 024 | 21.4 | 156.2 | 124.3 | 96.8 | 49.8 | 200 | 26.7 |
|  |  | 025 | 22.8 | 156.0 | 125.2 | 105.8 | 79.0 | 46.2 | 36.3 |
|  |  | 026 | 35.1 | 153.8 | 143.9 | 99.2 | 79.3 | 49.0 | 40.9 |
|  |  | 027 | 81.6 | 200 | 200 | 200 | 175.5 | 174.7 | 127.4 |
|  |  | 028 | 20.4 | 175.8 | 104.7 | 89.1 | 72.4 | 40.0 | 36.5 |
|  |  | 029 | 45.1 | 200 | 187.2 | 161.5 | 126.9 | 99.9 | 61.7 |
|  |  | 030 | 41.7 | 200 | 168.5 | 190 | 153.1 | 115.3 | 49.8 |
| ***Mean value*** | | | ***33*** | ***175*** | ***150*** | ***130*** | ***100*** | ***90*** | ***50*** |
| ***Geometrical mean value*** | | | ***30*** | ***174*** | ***150*** | ***120*** | ***90*** | ***80*** | ***50*** |
| ***Standard deviation*** | | | ***19*** | ***19*** | ***30*** | ***40*** | ***40*** | ***60*** | ***30*** |
| ***Coefficient of variation*** | | | ***58*** | ***11*** | ***20*** | ***30*** | ***40*** | ***60*** | ***60*** |
| ***Median*** | | | ***25*** | ***170*** | ***150*** | ***100*** | ***80*** | ***70*** | ***40*** |
| ***Lower 90% CI of Mean*** | | | ***23*** | ***165*** | ***130*** | ***110*** | ***70*** | ***60*** | ***30*** |
| ***Upper 90% CI of Mean*** | | | ***44*** | ***184*** | ***160*** | ***150*** | ***120*** | ***120*** | ***60*** |
| 4 | 0.75 mg/kg | 031 | 18.1 | 156.4 | 159.9 | 110.4 | 76.3 | 50.5 | 25.7 |
|  |  | 032 | 25.6 | 169.2 | 140.6 | 104.7 | 73.7 | 41.3 | 26.3 |
|  |  | 033 | 30.3 | 133.5 | 78.1 | 77.6 | 40.2 | 41.7 | 28.6 |
|  |  | 034 | 19.2 | 200 | 160.1 | 130.4 | 71.2 | 43.6 | 30.2 |
|  |  | 035 | 20.6 | 172.5 | 141.0 | 106.9 | 55.9 | 31.9 | 28.6 |
|  |  | 036 | 24.5 | 200 | 200 | 176.2 | 127.6 | 129.1 | 32.6 |
|  |  | 037 | 20.7 | 175.3 | 156.2 | 139.9 | 104.3 | 43.3 | 30.2 |
|  |  | 038 | 22.1 | 200 | 179.3 | 171.1 | 147.8 | 68.4 | 30.0 |
|  |  | 039 | 19.5 | 200 | 157.9 | 136.6 | 81.8 | 41.9 | 28.8 |
|  |  | 040 | 20.7 | 200 | 156.6 | 129.3 | 200 | 41.6 | 32.8 |
| ***Mean value*** | | | ***22*** | ***180*** | ***150*** | ***130*** | ***100*** | ***50*** | ***29*** |
| ***Geometrical mean value*** | | | ***22*** | ***180*** | ***150*** | ***130*** | ***90*** | ***50*** | ***29*** |
| ***Standard deviation*** | | | ***4*** | ***20*** | ***30*** | ***30*** | ***50*** | ***30*** | ***2*** |
| ***Coefficient of variation*** | | | ***17*** | ***10*** | ***20*** | ***20*** | ***50*** | ***50*** | ***8*** |
| ***Median*** | | | ***21*** | ***190*** | ***160*** | ***130*** | ***80*** | ***40*** | ***29*** |
| ***Lower 90% CI of Mean*** | | | ***20*** | ***170*** | ***140*** | ***110*** | ***70*** | ***40*** | ***28*** |
| ***Upper 90% CI of Mean*** | | | ***24*** | ***190*** | ***170*** | ***140*** | ***120*** | ***70*** | ***31*** |

**Table S9.** APTT of blood plasma samples drawn during 1 hour after the single injection of Thrombiveb® in rats.

| **Group #** | **Injected dose** | **Animal code** | **The time after drug injection, min** | | | | | | |
| --- | --- | --- | --- | --- | --- | --- | --- | --- | --- |
|  |  |  | **0** | **2** | **5** | **10** | **15** | **30** | **60** |
| 5 | 7.0 mg/kg | 041 | 20.4 | 50.3 | 33.4 | 28.6 | 25.1 | 21.2 | 22.1 |
|  |  | 042 | 21.3 | 47.4 | 30.0 | 25.8 | 25.6 | 25.0 | 24.7 |
|  |  | 043 | 22.0 | 52.8 | 30.2 | 23.9 | 23.1 | 22.1 | 22.9 |
|  |  | 044 | 21.2 | 53.4 | 31.8 | 24.3 | 22.2 | 20.2 | 20.2 |
|  |  | 045 | 21.4 | 47.7 | 32.0 | 24.1 | 23.0 | 21.1 | 20.2 |
|  |  | 046 | 19.3 | 46.6 | 29.0 | 23.5 | 22.1 | 21.5 | 21.7 |
|  |  | 047 | 20.7 | 47.0 | 24.6 | 20.6 | 21.3 | 20.1 | 19.0 |
|  |  | 048 | 16.3 | 36.9 | 29.9 | 19.2 | 18.9 | 17.6 | 17.1 |
|  |  | 049 | 18.8 | 49.6 | 23.7 | 23.2 | 21.4 | 19.8 | 19.7 |
|  |  | 050 | 18.0 | 42.1 | 26.4 | 21.1 | 20.9 | 18.6 | 17.4 |
| ***Mean value*** | | | ***19.9*** | ***47*** | ***29*** | ***23*** | ***22*** | ***21*** | ***21*** |
| ***Geometrical mean value*** | | | ***19.9*** | ***47*** | ***29*** | ***23*** | ***22*** | ***21*** | ***20*** |
| ***Standard deviation*** | | | ***1.8*** | ***5*** | ***3*** | ***3*** | ***2*** | ***2*** | ***2*** |
| ***Coefficient of variation*** | | | ***9.1*** | ***10*** | ***11*** | ***12*** | ***9*** | ***10*** | ***12*** |
| ***Median*** | | | ***20.6*** | ***48*** | ***30*** | ***24*** | ***22*** | ***21*** | ***20*** |
| ***Lower 90% CI of Mean*** | | | ***19.0*** | ***45*** | ***27*** | ***22*** | ***21*** | ***20*** | ***19*** |
| ***Upper 90% CI of Mean*** | | | ***20.9*** | ***50*** | ***31*** | ***25*** | ***23*** | ***22*** | ***22*** |
| 6 | 21 mg/kg | 051 | 20.0 | 90.0 | 55.0 | 38.0 | 34.3 | 30.7 | 26.9 |
|  |  | 052 | 24.1 | 90.4 | 88.0 | 46.2 | 40.8 | 37.3 | 38.8 |
|  |  | 053 | 25.2 | 83.6 | 58.3 | 50.7 | 42.1 | 39.1 | 36.1 |
|  |  | 054 | 21.8 | 187.4 | 69.9 | 65.9 | 45.2 | 41.1 | 62.6 |
|  |  | 055 | 41.1 | 200 | 110.2 | 135.1 | 58.3 | 97.4 | 80.3 |
|  |  | 056 | 63.2 | 200 | 169.4 | 139.2 | 137.7 | 141.4 | 118.3 |
|  |  | 057 | 35.3 | 200 | 116.8 | 62.9 | 78.2 | 94.1 | 45.5 |
|  |  | 058 | 33.0 | 184.1 | 73.5 | 78.7 | 95.2 | 144.7 | 84.9 |
|  |  | 059 | 41.9 | 168.3 | 140.6 | 93.9 | 93.7 | 73.4 | 61.7 |
|  |  | 060 | 33.0 | 144.7 | 116.1 | 59.8 | 44.9 | 33.1 | 58.0 |
| ***Mean value*** | | | ***34*** | ***150*** | ***100*** | ***80*** | ***70*** | ***70*** | ***60*** |
| ***Geometrical mean value*** | | | ***32*** | ***150*** | ***90*** | ***70*** | ***60*** | ***60*** | ***60*** |
| ***Standard deviation*** | | | ***13*** | ***50*** | ***40*** | ***40*** | ***30*** | ***40*** | ***30*** |
| ***Coefficient of variation*** | | | ***38*** | ***30*** | ***40*** | ***50*** | ***50*** | ***60*** | ***40*** |
| ***Median*** | | | ***33*** | ***180*** | ***100*** | ***60*** | ***50*** | ***60*** | ***60*** |
| ***Lower 90% CI of Mean*** | | | ***27*** | ***130*** | ***80*** | ***60*** | ***50*** | ***50*** | ***50*** |
| ***Upper 90% CI of Mean*** | | | ***41*** | ***180*** | ***120*** | ***100*** | ***80*** | ***100*** | ***80*** |
| 7 | 42 mg/kg | 061 | 43.3 | 200 | 179.9 | 61.4 | 102.1 | 62.4 | 92.2 |
|  |  | 062 | 61.8 | 200 | 160.4 | 127.4 | 109.8 | 97.2 | 93.0 |
|  |  | 063 | 30.0 | 170.9 | 142.1 | 52.7 | 102.9 | 87.5 | 67.1 |
|  |  | 064 | 35.3 | 146.9 | 103.6 | 59.7 | 46.4 | 59.4 | 67.6 |
|  |  | 065 | 43.4 | 200 | 200 | 145.8 | 113.9 | 75.2 | 103.4 |
|  |  | 066 | 44.5 | 200 | 200 | 101.8 | 104.7 | 111.5 | 80.4 |
|  |  | 067 | 47.0 | 200 | 200 | 113.3 | 116.8 | 130.0 | 92.6 |
|  |  | 068 | 43.2 | 200 | 200 | 105.4 | 93.6 | 79.2 | 132.0 |
|  |  | 069 | 45.2 | 200 | 118.3 | 72.3 | 80.7 | 77.4 | 103.8 |
|  |  | 070 | 62.7 | 200 | 158.8 | 155.1 | 119.2 | 104.7 | 97.2 |
| ***Mean value*** | | | ***46*** | ***192*** | ***170*** | ***100*** | ***100*** | ***90*** | ***93*** |
| ***Geometrical mean value*** | | | ***45*** | ***191*** | ***160*** | ***90*** | ***100*** | ***90*** | ***91*** |
| ***Standard deviation*** | | | ***10*** | ***18*** | ***40*** | ***40*** | ***20*** | ***20*** | ***19*** |
| ***Coefficient of variation*** | | | ***22*** | ***10*** | ***20*** | ***40*** | ***20*** | ***30*** | ***20*** |
| ***Median*** | | | ***44*** | ***200*** | ***170*** | ***100*** | ***100*** | ***80*** | ***93*** |
| ***Lower 90% CI of Mean*** | | | ***40*** | ***180*** | ***150*** | ***80*** | ***90*** | ***80*** | ***83*** |
| ***Upper 90% CI of Mean*** | | | ***51*** | ***200*** | ***190*** | ***120*** | ***110*** | ***100*** | ***103*** |

**Table S10.** Thrombin time of blood plasma samples drawn during 1 hour after the single injection of saline solution in monkeys.

| **Group #** | **Injected dose** | **Animal code** | **The time after drug injection, min** | | | | | | |
| --- | --- | --- | --- | --- | --- | --- | --- | --- | --- |
|  |  |  | **0** | **2** | **5** | **10** | **15** | **30** | **60** |
| 8 | 200 µl | 71 | 22.6 | 25.1 | 24.7 | 19.5 | 20.9 | 21.9 | 21.8 |
|  |  | 72 | 24.8 | 23.8 | 23.7 | 27.5 | 26.1 | 23.5 | 22.5 |
|  |  | 73 | 19.2 | 24.6 | 20.9 | 22.6 | 21.8 | 24.7 | 28.2 |
|  |  | 74 | 23.9 | 22.3 | 22.7 | 23.0 | 22.0 | 23.3 | 22.5 |
|  |  | 75 | 23.0 | 27.0 | 27.0 | 25.6 | 25.6 | 27.4 | 28.0 |
|  |  | 76 | 23.6 | 23.7 | 23.0 | 23.4 | 24.7 | 26.3 | 24.1 |
|  |  | 77 | 25.2 | 24.8 | 20.7 | 24.7 | 25.2 | 24.3 | 26.7 |
|  |  | 78 | 28.2 | 22.1 | 26.1 | 23.4 | 25.5 | 24.2 | 26.7 |
| ***Mean value*** | | | ***24*** | ***24.2*** | ***24*** | ***24*** | ***24*** | ***24.5*** | ***25*** |
| ***Geometrical mean value*** | | | ***24*** | ***24.1*** | ***24*** | ***24*** | ***24*** | ***24.4*** | ***25*** |
| ***Standard deviation*** | | | ***3*** | ***1.6*** | ***2*** | ***2*** | ***2*** | ***1.7*** | ***3*** |
| ***Coefficient of variation*** | | | ***11*** | ***6.6*** | ***10*** | ***10*** | ***9*** | ***7.1*** | ***11*** |
| ***Median*** | | | ***24*** | ***24.2*** | ***23*** | ***23*** | ***25*** | ***24.3*** | ***25*** |
| ***Lower 90% CI of Mean*** | | | ***22*** | ***23.3*** | ***22*** | ***22*** | ***23*** | ***23.4*** | ***24*** |
| ***Upper 90% CI of Mean*** | | | ***25*** | ***25.1*** | ***25*** | ***25*** | ***25*** | ***25.5*** | ***27*** |

**Table S11.** Thrombin time of blood plasma samples drawn during 1 hour after the single injection of Angiox® in monkeys.

| **Group #** | **Injected dose** | **Animal code** | **The time after drug injection, min** | | | | | | | |
| --- | --- | --- | --- | --- | --- | --- | --- | --- | --- | --- |
|  |  |  | **0** | **2** | **5** | **10** | **15** | **30** | **60** | |
| 9 | 0.19 mg/kg | 79 | 25.9 | 200 | 200 | 200 | 135.5 | 69.5 | 32.4 | |
|  |  | 80 | 23.7 | 200 | 200 | 146.7 | 109.4 | 74.5 | 30.6 | |
|  |  | 81 | 18.8 | 200 | 140.3 | 153.9 | 115.7 | 68.3 | 25.8 | |
|  |  | 82 | 23.6 | 200 | 177.7 | 159.9 | 105.9 | 56.8 | 30.3 | |
|  |  | 83 | 27.2 | 200 | 172.7 | 78.1 | 63.9 | 53.4 | 72.7 | |
|  |  | 84 | 26.3 | 200 | 200 | 171.7 | 160.1 | 122.7 | 65.1 | |
|  |  | 85 | 24.5 | 170.9 | 171.7 | 157.3 | 149.3 | 108.7 | 49.1 | |
|  |  | 86 | 23.9 | 200 | 200 | 161.2 | 100.9 | 60.9 | 37.6 | |
| ***Mean value*** | | | ***24*** | ***200*** | ***180*** | ***150*** | ***120*** | ***80*** | ***43*** | |
| ***Geometrical mean value*** | | | ***24*** | ***200*** | ***180*** | ***150*** | ***110*** | ***70*** | ***40*** | |
| ***Standard deviation*** | | | ***3*** | ***10*** | ***20*** | ***30*** | ***30*** | ***30*** | ***18*** | |
| ***Coefficient of variation*** | | | ***11*** | ***5*** | ***10*** | ***20*** | ***30*** | ***30*** | ***41*** | |
| ***Median*** | | | ***24*** | ***200*** | ***190*** | ***160*** | ***110*** | ***70*** | ***35*** | |
| ***Lower 90% CI of Mean*** | | | ***23*** | ***190*** | ***170*** | ***130*** | ***100*** | ***60*** | ***33*** | |
| ***Upper 90% CI of Mean*** | | | ***26*** | ***200*** | ***200*** | ***170*** | ***140*** | ***90*** | ***53*** | |
| 10 | 0.38 mg/kg | 87 | 28.2 | 200 | 200 | 200 | 200 | 200 | 162.3 | |
|  |  | 88 | 29.1 | 200 | 200 | 200 | 200 | 200 | 92.7 | |
|  |  | 89 | 26.9 | 200 | 200 | 200 | 200 | 200 | 148.6 | |
|  |  | 90 | 30.4 | 200 | 200 | 200 | 200 | 156.5 | 138.9 | |
|  |  | 91 | 32.4 | 200 | 200 | 200 | 200 | 200 | 141.9 | |
|  |  | 92 | 26.2 | 200 | 200 | 160.5 | 154.3 | 200 | 159.1 | |
|  |  | 93 | 28.8 | 200 | 200 | 200 | 200 | 200 | 156.9 | |
|  |  | 94 | 27.4 | 200 | 200 | 200 | 200 | 200 | 126.5 | |
| ***Mean value*** | | | ***29*** | ***200*** | ***200*** | ***200*** | ***194*** | ***195*** | ***140*** | |
| ***Geometrical mean value*** | | | ***29*** | ***200*** | ***200*** | ***200*** | ***194*** | ***194*** | ***140*** | |
| ***Standard deviation*** | | | ***2*** | ***-*** | ***-*** | ***-*** | ***16*** | ***15*** | ***20*** | |
| ***Coefficient of variation*** | | | ***7*** | ***-*** | ***-*** | ***-*** | ***8*** | ***8*** | ***20*** | |
| ***Median*** | | | ***29*** | ***200*** | ***200*** | ***200*** | ***200*** | ***200*** | ***150*** | |
| ***Lower 90% CI of Mean*** | | | ***28*** | ***-*** | ***-*** | ***-*** | ***185*** | ***186*** | ***130*** | |
| ***Upper 90% CI of Mean*** | | | ***30*** | ***-*** | ***-*** | ***-*** | ***204*** | ***204*** | ***150*** | |
| 11 | 0.75 mg/kg | 95 | 22.2 | 200 | 200 | 200 | 200 | 139.3 | 106.9 | |
|  |  | 96 | 24.5 | 200 | 200 | 200 | 200 | 200 | 175.3 | |
|  |  | 97 | 23.0 | 200 | 200 | 200 | 200 | 200 | 200 | |
|  |  | 98 | 21.4 | 200 | 200 | 200 | 200 | 200 | 200 | |
|  |  | 99 | 21.8 | 200 | 200 | 200 | 200 | 200 | 200 | |
|  |  | 100 | 23.9 | 200 | 200 | 200 | 200 | 200 | 51.6 | |
|  |  | 101 | 26.0 | 200 | 200 | 200 | 200 | 146.5 | 26.0 |  |
|  |  | 102 | 27.5 | 200 | 200 | 200 | 200 | 200 | 82.5 |  |
| ***Mean value*** | | | ***24*** | ***200*** | ***200*** | ***200*** | ***200*** | ***190*** | ***130*** | |
| ***Geometrical mean value*** | | | ***24*** | ***200*** | ***200*** | ***200*** | ***200*** | ***180*** | ***110*** | |
| ***Standard deviation*** | | | ***2*** | ***-*** | ***-*** | ***-*** | ***-*** | ***30*** | ***70*** | |
| ***Coefficient of variation*** | | | ***9*** | ***-*** | ***-*** | ***-*** | ***-*** | ***10*** | ***60*** | |
| ***Median*** | | | ***24*** | ***200*** | ***200*** | ***200*** | ***200*** | ***200*** | ***140*** | |
| ***Lower 90% CI of Mean*** | | | ***23*** | ***-*** | ***-*** | ***-*** | ***-*** | ***170*** | ***90*** | |
| ***Upper 90% CI of Mean*** | | | ***25*** | ***-*** | ***-*** | ***-*** | ***-*** | ***200*** | ***170*** | |

**Table S12.** Thrombin time of blood plasma samples drawn during 1 hour after the single injection of Thrombiveb® in monkeys.

| **Group #** | **Injected dose** | **Animal code** | **The time after drug injection, min** | | | | | | | |
| --- | --- | --- | --- | --- | --- | --- | --- | --- | --- | --- |
|  |  |  | **0** | **2** | **5** | | **10** | **15** | **30** | **60** |
| 12 | 7.0 mg/kg | 103 | 24.7 | 88.9 | 41.8 | | 22.4 | 33.5 | 30.0 | 32.5 |
|  |  | 104 | 22.9 | 67.7 | 28.9 | | 25.3 | 23.3 | 24.1 | 22.9 |
|  |  | 105 | 23.8 | 200 | 53.4 | | 26.7 | 26.3 | 25.5 | 12.5 |
|  |  | 106 | 28.9 | 145.9 | 45.7 | | 29.9 | 29.0 | 26.4 | 27.3 |
|  |  | 107 | 26.1 | 153.1 | 82.7 | | 50.9 | 26.4 | 23.3 | 24.5 |
|  |  | 108 | 21.1 | 108.5 | 45.7 | | 38.2 | 25.0 | 24.7 | 26.7 |
|  |  | 109 | 25.0 | 200 | 103.2 | | 53.8 | 27.7 | 26.3 | 24.5 |
|  |  | 110 | 25.8 | 43.6 | 30.6 | | 24.9 | 22.6 | 22.3 | 24.6 |
| ***Mean value*** | | | ***25*** | ***130*** | ***50*** | | ***34*** | ***27*** | ***25*** | ***24*** |
| ***Geometrical mean value*** | | | ***25*** | ***110*** | ***50*** | | ***32*** | ***27*** | ***25*** | ***24*** |
| ***Standard deviation*** | | | ***2*** | ***60*** | ***30*** | | ***12*** | ***4*** | ***2*** | ***6*** |
| ***Coefficient of variation*** | | | ***9*** | ***50*** | ***50*** | | ***36*** | ***13*** | ***9*** | ***15*** |
| ***Median*** | | | ***25*** | ***130*** | ***50*** | | ***28*** | ***26*** | ***25*** | ***25*** |
| ***Lower 90% CI of Mean*** | | | ***23*** | ***90*** | ***40*** | | ***27*** | ***25*** | ***24*** | ***23*** |
| ***Upper 90% CI of Mean*** | | | ***26*** | ***160*** | ***70*** | | ***41*** | ***29*** | ***27*** | ***25*** |
| 13 | 14 mg/kg | 111 | 24.2 | 200 | 98.7 | | 41.7 | 37.0 | 27.7 | 21.9 |
|  |  | 112 | 30.8 | 200 | 94.1 | | 44.2 | 42.8 | 33.8 | 29.6 |
|  |  | 113 | 23.8 | 200 | 75.7 | | 47.6 | 35.7 | 29.7 | 24.5 |
|  |  | 114 | 24.5 | 145.7 | 79.1 | | 50.6 | 35.1 | 28.9 | 24.6 |
|  |  | 115 | 21.7 | 91.1 | 75.3 | | 41.9 | 35.7 | 26.1 | 22.8 |
|  |  | 116 | 23.7 | 160.1 | 66.5 | | 47.6 | 46.7 | 23.9 | 28.9 |
|  |  | 117 | 25.5 | 142.9 | 65.7 | | 47.9 | 43.1 | 36.3 | 26.4 |
|  |  | 118 | 25.3 | 160.3 | 63.9 | | 41.2 | 42.2 | 25.9 | 25.4 |
| ***Mean value*** | | | ***25*** | ***160*** | ***77*** | | ***45*** | ***40*** | ***29*** | ***26*** |
| ***Geometrical mean value*** | | | ***25*** | ***160*** | ***77*** | | ***45*** | ***40*** | ***29*** | ***25*** |
| ***Standard deviation*** | | | ***3*** | ***40*** | ***13*** | | ***4*** | ***4*** | ***4*** | ***3*** |
| ***Coefficient of variation*** | | | ***11*** | ***20*** | ***17*** | | ***8*** | ***11*** | ***14*** | ***11*** |
| ***Median*** | | | ***24*** | ***160*** | ***76*** | | ***46*** | ***40*** | ***28*** | ***25*** |
| ***Lower 90% CI of Mean*** | | | ***23*** | ***140*** | ***70*** | | ***43*** | ***37*** | ***27*** | ***24*** |
| ***Upper 90% CI of Mean*** | | | ***27*** | ***180*** | ***85*** | | ***47*** | ***42*** | ***32*** | ***27*** |
| 14 | 28 mg/kg | 119 | 23.9 | 140.5 | 95.9 | | 61.1 | 43.4 | 35.1 | 39.8 |
|  |  | 120 | 23.5 | 107.7 | 116.9 | | 60.3 | 48.5 | 84.7 | 49.5 |
|  |  | 121 | 24.0 | 131.7 | 123.7 | | 48.5 | 61.9 | 72.5 | 43.7 |
|  |  | 122 | 26.6 | 144.1 | 110.5 | | 84.1 | 60.1 | 43.6 | 37.5 |
|  |  | 123 | 23.5 | 107.1 | 137.1 | | 113.3 | 97.5 | 90.7 | 57.7 |
|  |  | 124 | 28.3 | 135.1 | 145.9 | | 108.3 | 101.3 | 92.3 | 53.7 |
|  |  | 125 | 28.9 | 121.3 | 124.1 | | 58.5 | 91.7 | 72.1 | 40.9 |
|  |  | 126 | 21.3 | 134.1 | 108.7 | | 92.9 | 88.1 | 58.9 | 32.4 |
| ***Mean value*** | | | ***25*** | ***128*** | ***120*** | | ***80*** | ***70*** | ***70*** | ***44*** |
| ***Geometrical mean value*** | | | ***25*** | ***127*** | ***119*** | | ***80*** | ***70*** | ***70*** | ***44*** |
| ***Standard deviation*** | | | ***3*** | ***14*** | ***16*** | | ***20*** | ***20*** | ***20*** | ***9*** |
| ***Coefficient of variation*** | | | ***11*** | ***11*** | ***13*** | | ***30*** | ***30*** | ***30*** | ***19*** |
| ***Median*** | | | ***24*** | ***133*** | ***120*** | | ***70*** | ***80*** | ***70*** | ***42*** |
| ***Lower 90% CI of Mean*** | | | ***24*** | ***119*** | ***111*** | | ***60*** | ***60*** | ***60*** | ***39*** |
| ***Upper 90% CI of Mean*** | | | ***27*** | ***136*** | ***130*** | ***90*** | | ***90*** | ***80*** | ***49*** |

**Table S13.** Prothrombin time of blood plasma samples drawn during 1 hour after the single injection of saline solution in monkeys.

| **Group #** | **Injected dose** | **Animal code** | **The time after drug injection, min** | | | | | | |
| --- | --- | --- | --- | --- | --- | --- | --- | --- | --- |
|  |  |  | **0** | **2** | **5** | **10** | **15** | **30** | **60** |
| 8 | 200 µl | 71 | 5.8 | 6.4 | 6.3 | 6.7 | 7.2 | 7.5 | 7.0 |
|  |  | 72 | 5.9 | 6.2 | 5.7 | 5.5 | 5.5 | 6.1 | 5.5 |
|  |  | 73 | 6.8 | 7.2 | 6.3 | 6.9 | 7.3 | 7.8 | 7.1 |
|  |  | 74 | 5.4 | 5.8 | 5.7 | 6.1 | 6.3 | 5.3 | 6.2 |
|  |  | 75 | 5.9 | 7.5 | 8.0 | 6.1 | 7.3 | 7.7 | 7.0 |
|  |  | 76 | 6.8 | 6.5 | 5.7 | 6.3 | 6.1 | 6.7 | 6.9 |
|  |  | 77 | 6.3 | 7.0 | 6.6 | 6.7 | 6.6 | 6.7 | 6.8 |
|  |  | 78 | 5.0 | 5.1 | 5.3 | 4.9 | 5.6 | 5.9 | 5.5 |
| ***Mean value*** | | | ***6.0*** | ***6.5*** | ***6.2*** | ***6.2*** | ***6.5*** | ***6.7*** | ***6.5*** |
| ***Geometrical mean value*** | | | ***6.0*** | ***6.4*** | ***6.2*** | ***6.1*** | ***6.5*** | ***6.7*** | ***6.5*** |
| ***Standard deviation*** | | | ***0.6*** | ***0.8*** | ***0.8*** | ***0.7*** | ***0.7*** | ***0.9*** | ***0.7*** |
| ***Coefficient of variation*** | | | ***10.5*** | ***12.1*** | ***13.6*** | ***11.0*** | ***11.3*** | ***13.6*** | ***10.4*** |
| ***Median*** | | | ***5.9*** | ***6.5*** | ***6.0*** | ***6.2*** | ***6.5*** | ***6.7*** | ***6.9*** |
| ***Lower 90% CI of Mean*** | | | ***5.6*** | ***6.0*** | ***5.7*** | ***5.8*** | ***6.1*** | ***6.2*** | ***6.1*** |
| ***Upper 90% CI of Mean*** | | | ***6.4*** | ***6.9*** | ***6.7*** | ***6.5*** | ***6.9*** | ***7.2*** | ***6.9*** |

**Table S14.** Prothrombin time of blood plasma samples drawn during 1 hour after the single injection of Angiox® in monkeys.

| **Group #** | **Injected dose** | | **Animal code** | **The time after drug injection, min** | | | | | | |
| --- | --- | --- | --- | --- | --- | --- | --- | --- | --- | --- |
|  |  |  |  | **0** | **2** | **5** | **10** | **15** | **30** | **60** |
| 9 | 0.19 mg/kg | | 79 | 6.5 | 27.5 | 13.2 | 10.5 | 8.1 | 7.1 | 6.8 |
|  |  |  | 80 | 6.3 | 38.2 | 20.9 | 12.2 | 9.1 | 7.7 | 7.7 |
|  |  |  | 81 | 6.5 | 29.7 | 15.7 | 11.5 | 11.3 | 9.3 | 6.9 |
|  |  |  | 82 | 6.3 | 25.3 | 9.4 | 8.3 | 7.5 | 7.3 | 6.5 |
|  |  |  | 83 | 5.1 | 49.8 | 21.3 | 11.0 | 11.3 | 6.6 | 6.3 |
|  |  |  | 84 | 6.1 | 27.4 | 16.4 | 11.7 | 9.3 | 7.9 | 7.0 |
|  |  |  | 85 | 6.9 | 18.6 | 12.7 | 11.3 | 8.0 | 7.5 | 6.8 |
|  |  |  | 86 | 5.8 | 12.8 | 10.7 | 7.9 | 7.4 | 6.4 | 6.1 |
| ***Mean value*** | | | | ***6.2*** | ***29*** | ***15*** | ***10.6*** | ***9.0*** | ***7.5*** | ***6.8*** |
| ***Geometrical mean value*** | | | | ***6.2*** | ***27*** | ***15*** | ***10.4*** | ***8.9*** | ***7.4*** | ***6.7*** |
| ***Standard deviation*** | | | | ***0.5*** | ***11*** | ***4*** | ***1.6*** | ***1.6*** | ***0.9*** | ***0.5*** |
| ***Coefficient of variation*** | | | | ***8.8*** | ***40*** | ***29*** | ***15.1*** | ***17.5*** | ***12.0*** | ***7.2*** |
| ***Median*** | | | | ***6.3*** | ***28*** | ***15*** | ***11.2*** | ***8.6*** | ***7.4*** | ***6.8*** |
| ***Lower 90% CI of Mean*** | | | | ***5.9*** | ***22*** | ***13*** | ***9.6*** | ***8.1*** | ***7.0*** | ***6.5*** |
| ***Upper 90% CI of Mean*** | | | | ***6.5*** | ***35*** | ***18*** | ***11.5*** | ***9.9*** | ***8.0*** | ***7.0*** |
| 10 | 0.38 mg/kg | | 87 | 6.4 | 23.8 | 14.7 | 11.0 | 10.6 | 9.5 | 7.9 |
|  |  |  | 88 | 7.2 | 34.3 | 26.1 | 22.3 | 17.6 | 13.6 | 9.2 |
|  |  |  | 89 | 6.4 | 40.9 | 34.5 | 23.4 | 17.4 | 10.8 | 8.3 |
|  |  |  | 90 | 7.4 | 49.5 | 39.2 | 26.7 | 42.9 | 26.3 | 8.0 |
|  |  |  | 91 | 6.8 | 63.1 | 38.4 | 31.3 | 25.9 | 14.0 | 9.2 |
|  |  |  | 92 | 5.4 | 62.1 | 35.7 | 27.5 | 18.9 | 13.4 | 7.0 |
|  |  |  | 93 | 5.3 | 38.3 | 24.1 | 12.6 | 12.2 | 7.9 | 6.5 |
|  |  |  | 94 | 7.5 | 11.9 | 11.0 | 11.6 | 17.9 | 10.9 | 9.0 |
| ***Mean value*** | | | | ***6.6*** | ***41*** | ***28*** | ***21*** | ***20*** | ***13*** | ***8.1*** |
| ***Geometrical mean value*** | | | | ***6.5*** | ***36*** | ***26*** | ***19*** | ***19*** | ***13*** | ***8.1*** |
| ***Standard deviation*** | | | | ***0.8*** | ***18*** | ***11*** | ***8*** | ***10*** | ***6*** | ***1.0*** |
| ***Coefficient of variation*** | | | | ***13.0*** | ***44*** | ***39*** | ***38*** | ***50*** | ***43*** | ***12.4*** |
| ***Median*** | | | | ***6.6*** | ***40*** | ***30*** | ***23*** | ***18*** | ***12*** | ***8.2*** |
| ***Lower 90% CI of Mean*** | | | | ***6.1*** | ***30*** | ***22*** | ***16*** | ***15*** | ***10*** | ***7.6*** |
| ***Upper 90% CI of Mean*** | | | | ***7.0*** | ***51*** | ***34*** | ***25*** | ***26*** | ***17*** | ***8.7*** |
| 11 | | 0.75 mg/kg | 95 | 8.0 | 77.4 | 61.1 | 42.1 | 31.2 | 16.0 | 8.1 |
|  |  |  | 96 | 7.1 | 96.1 | 40.7 | 48.8 | 38.4 | 20.8 | 11.2 |
|  |  |  | 97 | 7.2 | 39.7 | 28.0 | 34.1 | 19.9 | 11.6 | 9.2 |
|  |  |  | 98 | 8.5 | 86.6 | 71.6 | 57.6 | 52.6 | 26.5 | 41.7 |
|  |  |  | 99 | 6.4 | 82.3 | 54.5 | 38.6 | 31.7 | 16.3 | 8.3 |
|  |  |  | 100 | 6.2 | 58.6 | 36.9 | 22.4 | 15.0 | 8.8 | 5.9 |
|  |  |  | 101 | 7.2 | 52.3 | 40.2 | 30.4 | 17.6 | 8.4 | 7.7 |
|  |  |  | 102 | 7.4 | 72.7 | 56.7 | 43.0 | 33.9 | 17.2 | 11.3 |
| ***Mean value*** | | | | ***7.3*** | ***71*** | ***49*** | ***40*** | ***30*** | ***16*** | ***13*** |
| ***Geometrical mean value*** | | | | ***7.2*** | ***68*** | ***47*** | ***38*** | ***28*** | ***15*** | ***11*** |
| ***Standard deviation*** | | | | ***0.8*** | ***19*** | ***15*** | ***11*** | ***12*** | ***6*** | ***12*** |
| ***Coefficient of variation*** | | | | ***10.4*** | ***27*** | ***30*** | ***28*** | ***41*** | ***39*** | ***92*** |
| ***Median*** | | | | ***7.2*** | ***75*** | ***48*** | ***40*** | ***32*** | ***16*** | ***9*** |
| ***Lower 90% CI of Mean*** | | | | ***6.8*** | ***60*** | ***40*** | ***33*** | ***23*** | ***12*** | ***6*** |
| ***Upper 90% CI of Mean*** | | | | ***7.7*** | ***82*** | ***57*** | ***46*** | ***37*** | ***19*** | ***20*** |

**Table S15.** Prothrombin time of blood plasma samples drawn during 1 hour after the single injection of Thrombiveb® in monkeys.

| **Group #** | | **Injected dose** | **Animal code** | **The time after drug injection, min** | | | | | | |
| --- | --- | --- | --- | --- | --- | --- | --- | --- | --- | --- |
|  |  |  |  | **0** | **2** | **5** | **10** | **15** | **30** | **60** |
| 12 | | 7.0 mg/kg | 103 | 6.9 | 19.0 | 9.2 | 6.8 | 7.9 | 7.3 | 7.7 |
|  |  |  | 104 | 5.7 | 9.9 | 6.7 | 7.7 | 7.5 | 5.7 | 5.7 |
|  |  |  | 105 | 7.5 | 19.3 | 5.7 | 6.7 | 6.9 | 6.9 | 7.3 |
|  |  |  | 106 | 5.9 | 15.7 | 6.6 | 6.0 | 6.6 | 5.7 | 6.7 |
|  |  |  | 107 | 7.0 | 15.0 | 8.6 | 6.5 | 6.9 | 6.1 | 6.6 |
|  |  |  | 108 | 5.2 | 17.7 | 5.8 | 6.6 | 6.3 | 6.5 | 5.4 |
|  |  |  | 109 | 6.5 | 15.6 | 7.8 | 7.2 | 7.0 | 7.1 | 5.3 |
|  |  |  | 110 | 7.8 | 7.4 | 7.5 | 6.6 | 5.6 | 6.7 | 7.8 |
| ***Mean value*** | | | | ***6.6*** | ***15*** | ***7.2*** | ***6.8*** | ***6.8*** | ***6.5*** | ***6.6*** |
| ***Geometrical mean value*** | | | | ***6.5*** | ***14*** | ***7.1*** | ***6.7*** | ***6.8*** | ***6.5*** | ***6.5*** |
| ***Standard deviation*** | | | | ***0.9*** | ***4*** | ***1.3*** | ***0.5*** | ***0.7*** | ***0.6*** | ***1.0*** |
| ***Coefficient of variation*** | | | | ***13.8*** | ***28*** | ***17.5*** | ***7.5*** | ***10.3*** | ***9.4*** | ***15.3*** |
| ***Median*** | | | | ***6.7*** | ***16*** | ***7.1*** | ***6.7*** | ***6.9*** | ***6.6*** | ***6.7*** |
| ***Lower 90% CI of Mean*** | | | | ***6.0*** | ***13*** | ***6.5*** | ***6.5*** | ***6.4*** | ***6.1*** | ***6.0*** |
| ***Upper 90% CI of Mean*** | | | | ***7.1*** | ***17*** | ***8.0*** | ***7.1*** | ***7.2*** | ***6.9*** | ***7.1*** |
| 13 | | 14 mg/kg | 111 | 6.7 | 16.6 | 10.8 | 7.3 | 6.7 | 6.4 | 6.0 |
|  |  |  | 112 | 6.0 | 18.4 | 8.2 | 5.7 | 5.8 | 6.0 | 5.5 |
|  |  |  | 113 | 6.9 | 21.6 | 11.1 | 6.0 | 7.1 | 7.0 | 6.0 |
|  |  |  | 114 | 6.0 | 19.3 | 10.5 | 7.8 | 7.0 | 7.3 | 6.9 |
|  |  |  | 115 | 6.5 | 16.9 | 10.0 | 7.4 | 7.0 | 6.9 | 6.0 |
|  |  |  | 116 | 6.7 | 17.3 | 10.1 | 6.8 | 5.6 | 7.0 | 6.8 |
|  |  |  | 117 | 6.2 | 16.8 | 8.9 | 7.7 | 6.4 | 6.4 | 6.2 |
|  |  |  | 118 | 5.5 | 17.1 | 9.6 | 8.3 | 6.3 | 6.0 | 6.0 |
| ***Mean value*** | | | | ***6.3*** | ***18.0*** | ***9.9*** | ***7.1*** | ***6.5*** | ***6.6*** | ***6.2*** |
| ***Geometrical mean value*** | | | | ***6.3*** | ***17.9*** | ***9.9*** | ***7.1*** | ***6.5*** | ***6.6*** | ***6.2*** |
| ***Standard deviation*** | | | | ***0.5*** | ***1.7*** | ***1.0*** | ***0.9*** | ***0.6*** | ***0.5*** | ***0.5*** |
| ***Coefficient of variation*** | | | | ***7.5*** | ***9.6*** | ***9.8*** | ***12.6*** | ***8.7*** | ***7.4*** | ***7.5*** |
| ***Median*** | | | | ***6.4*** | ***17.2*** | ***10.1*** | ***7.4*** | ***6.6*** | ***6.7*** | ***6.0*** |
| ***Lower 90% CI of Mean*** | | | | ***6.0*** | ***17.0*** | ***9.3*** | ***6.6*** | ***6.2*** | ***6.3*** | ***5.9*** |
| ***Upper 90% CI of Mean*** | | | | ***6.6*** | ***19.0*** | ***10.5*** | ***7.6*** | ***6.8*** | ***6.9*** | ***6.4*** |
| 14 | 28 mg/kg | | 119 | 6.8 | 22.0 | 14.2 | 8.0 | 7.5 | 7.5 | 7.3 |
|  |  |  | 120 | 5.8 | 21.1 | 10.3 | 8.2 | 7.3 | 7.0 | 6.5 |
|  |  |  | 121 | 5.8 | 22.8 | 18.1 | 8.7 | 7.6 | 8.0 | 6.8 |
|  |  |  | 122 | 6.0 | 21.9 | 8.5 | 8.5 | 7.4 | 6.9 | 6.3 |
|  |  |  | 123 | 7.0 | 26.4 | 19.0 | 11.4 | 10.0 | 8.0 | 5.7 |
|  |  |  | 124 | 5.9 | 18.1 | 15.5 | 6.9 | 6.3 | 5.6 | 7.0 |
|  |  |  | 125 | 5.7 | 17.6 | 16.0 | 6.3 | 11 | 7.6 | 6.6 |
|  |  |  | 126 | 6.0 | 19.1 | 14.6 | 9.4 | 8.0 | 7.6 | 7.0 |
| ***Mean value*** | | | | ***6.1*** | ***21*** | ***15*** | ***8.4*** | ***8.1*** | ***7.3*** | ***6.7*** |
| ***Geometrical mean value*** | | | | ***6.1*** | ***21*** | ***14*** | ***8.3*** | ***8.0*** | ***7.2*** | ***6.6*** |
| ***Standard deviation*** | | | | ***0.5*** | ***3*** | ***4*** | ***1.6*** | ***1.6*** | ***0.8*** | ***0.5*** |
| ***Coefficient of variation*** | | | | ***8.0*** | ***14*** | ***25*** | ***18.5*** | ***19.1*** | ***10.8*** | ***7.5*** |
| ***Median*** | | | | ***6.0*** | ***22*** | ***15*** | ***8.4*** | ***7.6*** | ***7.6*** | ***6.7*** |
| ***Lower 90% CI of Mean*** | | | | ***5.8*** | ***20*** | ***12*** | ***7.5*** | ***7.2*** | ***6.8*** | ***6.4*** |
| ***Upper 90% CI of Mean*** | | | | ***6.4*** | ***23*** | ***17*** | ***9.3*** | ***9.0*** | ***7.7*** | ***6.9*** |

**Table S16.** APTT of blood plasma samples drawn during 1 hour after the single injection of saline solution in monkeys.

| **Group #** | **Injected dose** | **Animal code** | **The time after drug injection, min** | | | | | | | |
| --- | --- | --- | --- | --- | --- | --- | --- | --- | --- | --- |
|  |  |  | **0** | **2** | **5** | **10** | **15** | **30** | | **60** |
| 8 | 200 µl | 71 | 19.7 | 21.7 | 24.3 | 32.7 | 23.6 | 35.6 | | 32.3 |
|  |  | 72 | 31.1 | 32.3 | 32.4 | 25.0 | 24.5 | 32.0 | | 23.6 |
|  |  | 73 | 35.7 | 37.6 | 23.6 | 35.5 | 35.7 | 35.0 | | 31.7 |
|  |  | 74 | 31.6 | 31.8 | 32.9 | 35.7 | 32.7 | 26.3 | | 26.3 |
|  |  | 75 | 32.3 | 32.0 | 33.3 | 35.4 | 21.4 | 31.1 | | 32.3 |
|  |  | 76 | 32.4 | 25.0 | 24.5 | 32.0 | 23.6 | 35.7 | | 30.9 |
|  |  | 77 | 26.2 | 25.7 | 37.6 | 23.6 | 35.5 | 29.5 | 35.0 | |
|  |  | 78 | 35.5 | 35.7 | 35.0 | 31.7 | 23.6 | 24.1 | 21.8 | |
| ***Mean value*** | | | ***31*** | ***30*** | ***31*** | ***32*** | ***28*** | ***31*** | ***29*** | |
| ***Geometrical mean value*** | | | ***30*** | ***30*** | ***30*** | ***31*** | ***27*** | ***31*** | ***29*** | |
| ***Standard deviation*** | | | ***5*** | ***6*** | ***6*** | ***5*** | ***6*** | ***4*** | ***5*** | |
| ***Coefficient of variation*** | | | ***17*** | ***18*** | ***18*** | ***15*** | ***22*** | ***14*** | ***16*** | |
| ***Median*** | | | ***32*** | ***32*** | ***33*** | ***32*** | ***24*** | ***32*** | ***31*** | |
| ***Lower 90% CI of Mean*** | | | ***28*** | ***27*** | ***27*** | ***29*** | ***24*** | ***29*** | ***27*** | |
| ***Upper 90% CI of Mean*** | | | ***34*** | ***33*** | ***34*** | ***34*** | ***31*** | ***34*** | ***32*** | |

**Table S17.** APTT of blood plasma samples drawn during 1 hour after the single injection of Angiox® in monkeys.

| **Group #** | **Injected dose** | | **Animal code** | **The time after drug injection, min** | | | | | | |
| --- | --- | --- | --- | --- | --- | --- | --- | --- | --- | --- |
|  |  |  |  | **0** | **2** | **5** | **10** | **15** | **30** | **60** |
| 9 | 0.19 mg/kg | | 79 | 25.6 | 80.9 | 64.8 | 51.0 | 44.8 | 28.0 | 26.7 |
|  |  |  | 80 | 28.5 | 91.3 | 71.9 | 54.5 | 43.1 | 38.0 | 30.2 |
|  |  |  | 81 | 26.3 | 79.7 | 55.8 | 60.0 | 51.8 | 44.5 | 32.8 |
|  |  |  | 82 | 20.6 | 78.9 | 51.5 | 44.4 | 31.0 | 25.6 | 21.3 |
|  |  |  | 83 | 26.7 | 86.4 | 61.4 | 42.9 | 59.3 | 33.8 | 30.2 |
|  |  |  | 84 | 23.3 | 59.1 | 63.7 | 57.3 | 45.7 | 37.5 | 27.6 |
|  |  |  | 85 | 25.4 | 58.4 | 61.3 | 53.2 | 40.9 | 35.8 | 27.0 |
|  |  |  | 86 | 20.1 | 65.4 | 51.7 | 39.5 | 35.3 | 25.4 | 22.0 |
| ***Mean value*** | | | | ***25*** | ***75*** | ***60*** | ***50*** | ***44*** | ***34*** | ***27*** |
| ***Geometrical mean value*** | | | | ***24*** | ***74*** | ***60*** | ***50*** | ***43*** | ***33*** | ***27*** |
| ***Standard deviation*** | | | | ***3*** | ***13*** | ***7*** | ***7*** | ***9*** | ***7*** | ***4*** |
| ***Coefficient of variation*** | | | | ***12*** | ***17*** | ***12*** | ***15*** | ***20*** | ***20*** | ***15*** |
| ***Median*** | | | | ***26*** | ***79*** | ***61*** | ***52*** | ***44*** | ***35*** | ***27*** |
| ***Lower 90% CI of Mean*** | | | | ***23*** | ***68*** | ***56*** | ***46*** | ***39*** | ***30*** | ***25*** |
| ***Upper 90% CI of Mean*** | | | | ***26*** | ***82*** | ***64*** | ***55*** | ***49*** | ***38*** | ***30*** |
| 10 | 0.38 mg/kg | | 87 | 22.9 | 78.3 | 63.3 | 52.7 | 43.8 | 38.9 | 32.2 |
|  |  |  | 88 | 25.4 | 81.4 | 76.4 | 48.6 | 62.8 | 53.7 | 36.7 |
|  |  |  | 89 | 24.3 | 94.1 | 86.7 | 76.5 | 58.3 | 43.8 | 38.5 |
|  |  |  | 90 | 34.5 | 100.1 | 59.7 | 44.5 | 57.8 | 43.5 | 27.3 |
|  |  |  | 91 | 21.9 | 107.3 | 79.0 | 69.2 | 59.4 | 42.6 | 23.8 |
|  |  |  | 92 | 21.5 | 109.1 | 70.8 | 67.0 | 39.9 | 47.2 | 28.5 |
|  |  |  | 93 | 16.9 | 76.8 | 59.7 | 45.7 | 43.3 | 32.4 | 21.8 |
|  |  |  | 94 | 23.5 | 46.8 | 45.4 | 44.8 | 56.0 | 39.6 | 39.6 |
| ***Mean value*** | | | | ***24*** | ***87*** | ***68*** | ***56*** | ***53*** | ***43*** | ***31*** |
| ***Geometrical mean value*** | | | | ***24*** | ***84*** | ***66*** | ***55*** | ***52*** | ***42*** | ***30*** |
| ***Standard deviation*** | | | | ***5*** | ***20*** | ***13*** | ***13*** | ***9*** | ***6*** | ***7*** |
| ***Coefficient of variation*** | | | | ***21*** | ***24*** | ***20*** | ***22*** | ***17*** | ***15*** | ***22*** |
| ***Median*** | | | | ***23*** | ***88*** | ***67*** | ***51*** | ***57*** | ***43*** | ***30*** |
| ***Lower 90% CI of Mean*** | | | | ***21*** | ***75*** | ***60*** | ***49*** | ***48*** | ***39*** | ***27*** |
| ***Upper 90% CI of Mean*** | | | | ***27*** | ***99*** | ***75*** | ***64*** | ***58*** | ***46*** | ***35*** |
| 11 | | 0.75 mg/kg | 95 | 26.2 | 186.1 | 155.5 | 110.6 | 91.1 | 66.2 | 46.5 |
|  |  |  | 96 | 26.9 | 168.1 | 200 | 118.6 | 85.6 | 65.1 | 61.8 |
|  |  |  | 97 | 27.8 | 112.8 | 96.1 | 93.3 | 82.4 | 63.1 | 50.6 |
|  |  |  | 98 | 45.0 | 200 | 200 | 190.0 | 181.9 | 160.1 | 143.0 |
|  |  |  | 99 | 29.2 | 159.0 | 123.4 | 114.8 | 106.7 | 80.0 | 65.9 |
|  |  |  | 100 | 24.2 | 129.8 | 85.1 | 82.9 | 53.1 | 43.2 | 26.8 |
|  |  |  | 101 | 36.8 | 167.4 | 139.0 | 125.6 | 96.3 | 53.9 | 36.8 |
|  |  |  | 102 | 32.7 | 161.5 | 140.1 | 122.3 | 85.1 | 66.2 | 43.7 |
| ***Mean value*** | | | | ***31*** | ***160*** | ***140*** | ***120*** | ***100*** | ***70*** | ***60*** |
| ***Geometrical mean value*** | | | | ***31*** | ***160*** | ***140*** | ***120*** | ***90*** | ***70*** | ***50*** |
| ***Standard deviation*** | | | | ***7*** | ***30*** | ***40*** | ***30*** | ***40*** | ***40*** | ***40*** |
| ***Coefficient of variation*** | | | | ***22*** | ***20*** | ***30*** | ***30*** | ***40*** | ***50*** | ***60*** |
| ***Median*** | | | | ***29*** | ***160*** | ***140*** | ***120*** | ***90*** | ***70*** | ***50*** |
| ***Lower 90% CI of Mean*** | | | | ***27*** | ***140*** | ***120*** | ***100*** | ***80*** | ***50*** | ***40*** |
| ***Upper 90% CI of Mean*** | | | | ***35*** | ***180*** | ***170*** | ***140*** | ***120*** | ***100*** | ***80*** |

**Table S18.** APTT of blood plasma samples drawn during 1 hour after the single injection of Thrombiveb® in monkeys.

| **Group #** | **Injected dose** | **Animal code** | **The time after drug injection, min** | | | | | | |
| --- | --- | --- | --- | --- | --- | --- | --- | --- | --- |
|  |  |  | **0** | **2** | **5** | **10** | **15** | **30** | **60** |
| 12 | 7.0 mg/kg | 103 | 23.6 | 74.6 | 39.0 | 32.9 | 37.8 | 25.8 | 33.6 |
|  |  | 104 | 38.0 | 40.6 | 45.9 | 37.1 | 28.8 | 30.9 | 38.0 |
|  |  | 105 | 27.5 | 116.1 | 27.2 | 28.3 | 27.6 | 26.6 | 27.8 |
|  |  | 106 | 33.2 | 107.3 | 47.2 | 33.3 | 37.7 | 31.8 | 29.1 |
|  |  | 107 | 23.1 | 100.1 | 32.8 | 26.2 | 28.6 | 21.8 | 24.1 |
|  |  | 108 | 27.0 | 98.1 | 36.3 | 27.0 | 30.0 | 27.3 | 25.7 |
|  |  | 109 | 20.7 | 83.1 | 106.0 | 30.9 | 29.2 | 27.5 | 28.1 |
|  |  | 110 | 34.2 | 120.0 | 44.0 | 33.6 | 32.8 | 33.8 | 30.0 |
| ***Mean value*** | | | ***28*** | ***100*** | ***50*** | ***31*** | ***32*** | ***28*** | ***30*** |
| ***Geometrical mean value*** | | | ***28*** | ***90*** | ***40*** | ***31*** | ***31*** | ***28*** | ***29*** |
| ***Standard deviation*** | | | ***6*** | ***50*** | ***20*** | ***4*** | ***4*** | ***4*** | ***4*** |
| ***Coefficient of variation*** | | | ***22*** | ***50*** | ***50*** | ***12*** | ***13*** | ***14*** | ***15*** |
| ***Median*** | | | ***27*** | ***100*** | ***40*** | ***32*** | ***30*** | ***27*** | ***29*** |
| ***Lower 90% CI of Mean*** | | | ***23*** | ***60*** | ***30*** | ***28*** | ***29*** | ***26*** | ***26*** |
| ***Upper 90% CI of Mean*** | | | ***34*** | ***140*** | ***70*** | ***33*** | ***34*** | ***30*** | ***33*** |
| 13 | 14 mg/kg | 111 | 24.2 | 115.0 | 49.5 | 49.5 | 26.5 | 26.1 | 23.7 |
|  |  | 112 | 28.7 | 121.0 | 49.9 | 49.9 | 31.8 | 29.2 | 25.3 |
|  |  | 113 | 27.2 | 147.2 | 77.6 | 77.6 | 44.6 | 30.8 | 27.0 |
|  |  | 114 | 28.7 | 129.8 | 78.6 | 78.6 | 35.4 | 28.4 | 28.8 |
|  |  | 115 | 19.3 | 81.2 | 58.8 | 58.8 | 30.0 | 25.0 | 22.8 |
|  |  | 116 | 19.5 | 84.6 | 46.5 | 46.5 | 25.5 | 20.4 | 21.9 |
|  |  | 117 | 32.0 | 117.9 | 72.0 | 72.0 | 39.6 | 34.0 | 30.6 |
|  |  | 118 | 27.4 | 120.3 | 71.3 | 71.3 | 41.3 | 36.1 | 35.1 |
| ***Mean value*** | | | ***26*** | ***110*** | ***63*** | ***63*** | ***34*** | ***29*** | ***27*** |
| ***Geometrical mean value*** | | | ***26*** | ***110*** | ***62*** | ***62*** | ***34*** | ***28*** | ***27*** |
| ***Standard deviation*** | | | ***5*** | ***20*** | ***13*** | ***13*** | ***7*** | ***5*** | ***5*** |
| ***Coefficient of variation*** | | | ***18*** | ***20*** | ***21*** | ***21*** | ***21*** | ***18*** | ***17*** |
| ***Median*** | | | ***27*** | ***120*** | ***65*** | ***65*** | ***34*** | ***29*** | ***26*** |
| ***Lower 90% CI of Mean*** | | | ***23*** | ***100*** | ***55*** | ***55*** | ***30*** | ***26*** | ***24*** |
| ***Upper 90% CI of Mean*** | | | ***29*** | ***130*** | ***71*** | ***71*** | ***38*** | ***32*** | ***30*** |
| 14 | 28 mg/kg | 119 | 22.8 | 154.3 | 82.1 | 47.6 | 37.2 | 38.2 | 31.1 |
|  |  | 120 | 28.3 | 200 | 174.4 | 123.0 | 95.2 | 63.0 | 43.0 |
|  |  | 121 | 33.5 | 190.0 | 147.6 | 108.7 | 75.1 | 59.7 | 44.2 |
|  |  | 122 | 30.6 | 200 | 200 | 97.2 | 88.2 | 67.0 | 43.0 |
|  |  | 123 | 29.8 | 200 | 145.6 | 111.7 | 85.5 | 79.4 | 34.9 |
|  |  | 124 | 31.9 | 168.5 | 131.1 | 82.3 | 68.4 | 47.8 | 55.0 |
|  |  | 125 | 19.8 | 150.0 | 99.0 | 65.0 | 64.3 | 30.7 | 25.0 |
|  |  | 126 | 25.9 | 157.7 | 105.6 | 76.7 | 46.5 | 52.6 | 34.2 |
| ***Mean value*** | | | ***28*** | ***180*** | ***140*** | ***90*** | ***70*** | ***55*** | ***39*** |
| ***Geometrical mean value*** | | | ***28*** | ***180*** | ***130*** | ***90*** | ***70*** | ***53*** | ***38*** |
| ***Standard deviation*** | | | ***5*** | ***20*** | ***40*** | ***30*** | ***20*** | ***16*** | ***9*** |
| ***Coefficient of variation*** | | | ***17*** | ***10*** | ***30*** | ***30*** | ***30*** | ***29*** | ***24*** |
| ***Median*** | | | ***29*** | ***180*** | ***140*** | ***90*** | ***70*** | ***56*** | ***39*** |
| ***Lower 90% CI of Mean*** | | | ***25*** | ***160*** | ***110*** | ***70*** | ***60*** | ***46*** | ***33*** |
| ***Upper 90% CI of Mean*** | | | ***31*** | ***190*** | ***160*** | ***100*** | ***80*** | ***64*** | ***44*** |

**Table S19.** Thrombin time of blood plasma samples drawn during multiple injections of saline solution in rats.

| **Group #** | **Injected dose** | **Animal code** | **The time after drug injection, min** | | | | | | |
| --- | --- | --- | --- | --- | --- | --- | --- | --- | --- |
|  |  |  | **0** | **5** | **20** | **35** | **50** | **65** | **90** |
| 15 | 200 µl  ×  5 times | 127 | 41.3 | 38.9 | 53.1 | 42.7 | 44.7 | 47.2 | 35.9 |
|  |  | 128 | 48.1 | 56.2 | 52.6 | 41.3 | 37.2 | 44.7 | 59.6 |
|  |  | 129 | 52.7 | 48.5 | 53.6 | 54.1 | 36.7 | 59.1 | 53.7 |
|  |  | 130 | 45.2 | 49.4 | 42.0 | 57.4 | 46.3 | 37.8 | 58.4 |
|  |  | 131 | 36.4 | 42.7 | 49.9 | 44.1 | 57.3 | 43.5 | 37.8 |
|  |  | 132 | 56.4 | 47.7 | 49.3 | 47.9 | 55.4 | 41.9 | 52.8 |
|  |  | 133 | 38.5 | 47.9 | 48.2 | 39.8 | 43.6 | 59.8 | 41.9 |
|  |  | 134 | 36.9 | 46.9 | 39.1 | 48.6 | 39.0 | 57.8 | 51.4 |
|  |  | 135 | 49.6 | 47.3 | 46.2 | 37.2 | 49.6 | 54.8 | 59.9 |
|  |  | 136 | 46.1 | 39.2 | 45.2 | 59.3 | 48.9 | 41.6 | 39.4 |
| ***Mean value*** | | | ***45*** | ***47*** | ***48*** | ***47*** | ***46*** | ***49*** | ***49*** |
| ***Geometrical mean value*** | | | ***45*** | ***46*** | ***48*** | ***47*** | ***45*** | ***48*** | ***48*** |
| ***Standard deviation*** | | | ***7*** | ***5*** | ***5*** | ***8*** | ***7*** | ***8*** | ***9*** |
| ***Coefficient of variation*** | | | ***15*** | ***11*** | ***10*** | ***16*** | ***16*** | ***17*** | ***19*** |
| ***Median*** | | | ***46*** | ***48*** | ***49*** | ***46*** | ***46*** | ***46*** | ***52*** |
| ***Lower 90% CI of Mean*** | | | ***42*** | ***44*** | ***45*** | ***43*** | ***42*** | ***45*** | ***44*** |
| ***Upper 90% CI of Mean*** | | | ***49*** | ***49*** | ***50*** | ***51*** | ***50*** | ***53*** | ***54*** |

**Table S20.** Thrombin time of blood plasma samples drawn during multiple injections of Angiox® in rats.

| **Group #** | **Injected dose** | **Animal code** | **The time after drug injection, min** | | | | | | |
| --- | --- | --- | --- | --- | --- | --- | --- | --- | --- |
|  |  |  | **0** | **7** | **15** | **30** | **45** | **65** | **90** |
| 16 | 0.19 mg/kg  ×  2 times | 137 | 50.3 | 200 | 200 | 200 | 200 | 200 | 126.3 |
|  |  | 138 | 54.1 | 200 | 200 | 200 | 200 | 200 | 200 |
|  |  | 139 | 53.5 | 200 | 200 | 200 | 200 | 200 | 159.7 |
|  |  | 140 | 58.9 | 200 | 200 | 200 | 200 | 200 | 173.1 |
|  |  | 141 | 51.8 | 200 | 200 | 200 | 200 | 200 | 162.7 |
|  |  | 142 | 58.9 | 200 | 200 | 158.5 | 200 | 175.1 | 122.9 |
|  |  | 143 | 54.7 | 200 | 200 | 200 | 200 | 200 | 144.1 |
|  |  | 144 | 54.1 | 200 | 200 | 200 | 200 | 200 | 142.3 |
|  |  | 145 | 53.5 | 200 | 200 | 164.9 | 200 | 200 | 165.1 |
|  |  | 146 | 54.1 | 200 | 200 | 200 | 200 | 182.1 | 160.3 |
| ***Mean value*** | | | ***54*** | ***200*** | ***200*** | ***192*** | ***200*** | ***196*** | ***160*** |
| ***Geometrical mean value*** | | | ***54*** | ***200*** | ***200*** | ***192*** | ***200*** | ***196*** | ***150*** |
| ***Standard deviation*** | | | ***3*** | ***-*** | ***-*** | ***16*** | ***-*** | ***9*** | ***20*** |
| ***Coefficient of variation*** | | | ***5*** | ***-*** | ***-*** | ***8*** | ***-*** | ***5*** | ***10*** |
| ***Median*** | | | ***54*** | ***200*** | ***200*** | ***200*** | ***200*** | ***200*** | ***160*** |
| ***Lower 90% CI of Mean*** | | | ***53*** | ***-*** | ***-*** | ***184*** | ***-*** | ***191*** | ***140*** |
| ***Upper 90% CI of Mean*** | | | ***56*** | ***-*** | ***-*** | ***200*** | ***-*** | ***200*** | ***170*** |
| 17 | 0.38 mg/kg  ×  2 times | 147 | 64.3 | 200 | 200 | 200 | 200 | 200 | 200 |
|  |  | 148 | 55.3 | 200 | 200 | 200 | 200 | 200 | 200 |
|  |  | 149 | 59.2 | 200 | 200 | 200 | 200 | 200 | 200 |
|  |  | 150 | 52.4 | 200 | 200 | 200 | 200 | 200 | 163.5 |
|  |  | 151 | 54.2 | 200 | 200 | 200 | 200 | 200 | 155.5 |
|  |  | 152 | 53.1 | 150.7 | 200 | 200 | 200 | 153.5 | 173.9 |
|  |  | 153 | 57.1 | 200 | 200 | 200 | 200 | 200 | 200 |
|  |  | 154 | 40.9 | 200 | 200 | 200 | 200 | 117.5 | 87.5 |
|  |  | 155 | 39.0 | 200 | 200 | 200 | 200 | 200 | 200 |
|  |  | 156 | 56.3 | 200 | 200 | 177.7 | 200 | 200 | 136.0 |
| ***Mean value*** | | | ***53*** | ***195*** | ***200*** | ***198*** | ***200*** | ***190*** | ***170*** |
| ***Geometrical mean value*** | | | ***53*** | ***194*** | ***200*** | ***198*** | ***200*** | ***180*** | ***170*** |
| ***Standard deviation*** | | | ***8*** | ***16*** | ***-*** | ***7*** | ***-*** | ***30*** | ***40*** |
| ***Coefficient of variation*** | | | ***15*** | ***8*** | ***-*** | ***4*** | ***-*** | ***15*** | ***20*** |
| ***Median*** | | | ***55*** | ***200*** | ***200*** | ***200*** | ***200*** | ***200*** | ***190*** |
| ***Lower 90% CI of Mean*** | | | ***49*** | ***187*** | ***-*** | ***194*** | ***-*** | ***170*** | ***150*** |
| ***Upper 90% CI of Mean*** | | | ***57*** | ***200*** | ***-*** | ***200*** | ***-*** | ***200*** | ***190*** |
| 18 | 0.75 mg/kg  ×  2 times | 157 | 52.9 | 200 | 200 | 200 | 200 | 200 | 200 |
|  |  | 158 | 61.5 | 200 | 200 | 200 | 200 | 200 | 137.0 |
|  |  | 159 | 41.8 | 200 | 200 | 200 | 200 | 200 | 200 |
|  |  | 160 | 52.3 | 200 | 200 | 200 | 200 | 200 | 200 |
|  |  | 161 | 60.9 | 200 | 200 | 200 | 200 | 200 | 200 |
|  |  | 162 | 59.7 | 200 | 200 | 200 | 200 | 200 | 200 |
|  |  | 163 | 52.1 | 155.5 | 107.5 | 200 | 200 | 200 | 178.1 |
|  |  | 164 | 58.1 | 200 | 200 | 200 | 200 | 200 | 200 |
|  |  | 165 | 54.7 | 200 | 200 | 200 | 200 | 200 | 179.2 |
|  |  | 166 | 67.5 | 200 | 200 | 200 | 200 | 200 | 200 |
| ***Mean value*** | | | ***56*** | ***196*** | ***190*** | ***200*** | ***200*** | ***200*** | ***190*** |
| ***Geometrical mean value*** | | | ***56*** | ***195*** | ***190*** | ***200*** | ***200*** | ***200*** | ***190*** |
| ***Standard deviation*** | | | ***7*** | ***14*** | ***30*** | ***-*** | ***-*** | ***-*** | ***20*** |
| ***Coefficient of variation*** | | | ***13*** | ***7*** | ***15*** | ***-*** | ***-*** | ***-*** | ***10*** |
| ***Median*** | | | ***56*** | ***200*** | ***200.0*** | ***200*** | ***200*** | ***200*** | ***200*** |
| ***Lower 90% CI of Mean*** | | | ***53*** | ***188*** | ***180*** | ***-*** | ***-*** | ***-*** | ***180*** |
| ***Upper 90% CI of Mean*** | | | ***60*** | ***203*** | ***200*** | ***-*** | ***-*** | ***-*** | ***200*** |

**Table S21.** Thrombin time of blood plasma samples drawn during multiple injections of Thrombiveb® in rats.

| **Group #** | **Injected dose** | **Animal code** | **The time after drug injection, min** | | | | | | |
| --- | --- | --- | --- | --- | --- | --- | --- | --- | --- |
|  |  |  | **0** | **5** | **20** | **35** | **50** | **65** | **90** |
| 19 | 7.0 mg/kg  ×  5 times | 167 | 47.4 | 125.7 | 150.7 | 145.1 | 156.3 | 200 | 60.3 |
|  |  | 168 | 52.5 | 156.9 | 200 | 175.1 | 200 | 200 | 73.9 |
|  |  | 169 | 52.1 | 152.7 | 179.5 | 160.1 | 200 | 145.9 | 55.4 |
|  |  | 170 | 53.7 | 102.5 | 96.3 | 121.7 | 164.5 | 89.5 | 52.5 |
|  |  | 171 | 50.5 | 168.7 | 200 | 149.1 | 200 | 200 | 78.9 |
|  |  | 172 | 57.3 | 141.3 | 133.1 | 159.5 | 138.3 | 154.9 | 74.1 |
|  |  | 173 | 57.3 | 153.7 | 113.9 | 152.9 | 140.9 | 200 | 55.0 |
|  |  | 174 | 54.5 | 148.3 | 155.1 | 150.9 | 200 | 200 | 62.9 |
|  |  | 175 | 48.9 | 152.1 | 163.1 | 200 | 200 | 200 | 67.3 |
|  |  | 176 | 50.9 | 138.7 | 139.3 | 122.9 | 117.9 | 172.9 | 47.2 |
| ***Mean value*** | | | ***53*** | ***144*** | ***150*** | ***150*** | ***170*** | ***180*** | ***63*** |
| ***Geometrical mean value*** | | | ***52*** | ***143*** | ***150*** | ***150*** | ***170*** | ***170*** | ***62*** |
| ***Standard deviation*** | | | ***3*** | ***19*** | ***30*** | ***20*** | ***30*** | ***40*** | ***11*** |
| ***Coefficient of variation*** | | | ***6*** | ***13*** | ***20*** | ***10*** | ***20*** | ***20*** | ***17*** |
| ***Median*** | | | ***52*** | ***150*** | ***150*** | ***150*** | ***180*** | ***200*** | ***62*** |
| ***Lower 90% CI of Mean*** | | | ***51*** | ***134*** | ***140*** | ***140*** | ***160*** | ***1601*** | ***57*** |
| ***Upper 90% CI of Mean*** | | | ***54*** | ***151*** | ***170*** | ***170*** | ***190*** | ***200*** | ***68*** |
| 20 | 21 mg/kg ×  5 times | 177 | 45.1 | 200 | 200 | 200 | 200 | 200 | 59.7 |
|  |  | 178 | 50.3 | 200 | 200 | 200 | 200 | 200 | 116.7 |
|  |  | 179 | 50.9 | 200 | 200 | 200 | 200 | 200 | 112.1 |
|  |  | 180 | 49.3 | 200 | 200 | 200 | 200 | 200 | 123.6 |
|  |  | 181 | 54.9 | 200 | 200 | 200 | 200 | 200 | 114.3 |
|  |  | 182 | 52.3 | 200 | 169.3 | 200 | 200 | 200 | 131.3 |
|  |  | 183 | 50.1 | 200 | 200 | 200 | 200 | 200 | 135.2 |
|  |  | 184 | 52.7 | 200 | 200 | 200 | 200 | 200 | 200 |
|  |  | 185 | 48.1 | 173.5 | 200 | 200 | 200 | 200 | 170.3 |
|  |  | 186 | 45.1 | 163.9 | 200 | 143.7 | 113.7 | 146.7 | 75.7 |
| ***Mean value*** | | | ***50*** | ***194*** | ***197*** | ***194*** | ***191*** | ***195*** | ***120*** |
| ***Geometrical mean value*** | | | ***50*** | ***193*** | ***197*** | ***194*** | ***189*** | ***194*** | ***120*** |
| ***Standard deviation*** | | | ***3*** | ***13*** | ***10*** | ***18*** | ***30*** | ***17*** | ***40*** |
| ***Coefficient of variation*** | | | ***6*** | ***7*** | ***5*** | ***9*** | ***14*** | ***9*** | ***30*** |
| ***Median*** | | | ***50*** | ***200*** | ***200*** | ***200*** | ***200*** | ***200*** | ***120*** |
| ***Lower 90% CI of Mean*** | | | ***48*** | ***187*** | ***192*** | ***185*** | ***177*** | ***186*** | ***100*** |
| ***Upper 90% CI of Mean*** | | | ***52*** | ***200*** | ***200*** | ***200*** | ***200*** | ***200*** | ***150*** |
| 21 | 42 mg/kg ×  5 times | 187 | 52.1 | 200 | 200 | 200 | 200 | 200 | 88.3 |
|  |  | 188 | 59.9 | 200 | 200 | 200 | 200 | 200 | 200 |
|  |  | 189 | 50.1 | 200 | 200 | 200 | 200 | 200 | 200 |
|  |  | 190 | 50.7 | 200 | 200 | 200 | 200 | 200 | 153.1 |
|  |  | 191 | 52.1 | 200 | 200 | 200 | 200 | 200 | 200 |
|  |  | 192 | 51.3 | 200 | 200 | 200 | 200 | 200 | 200 |
|  |  | 193 | 48.8 | 200 | 200 | 200 | 200 | 200 | 128.9 |
|  |  | 194 | 46.0 | 200 | 200 | 200 | 200 | 171.1 | 162.1 |
|  |  | 195 | 54.7 | 200 | 200 | 200 | 200 | 200 | 200 |
|  |  | 196 | 54.9 | 200 | 200 | 200 | 200 | 200 | 200 |
| ***Mean value*** | | | ***52*** | ***200*** | ***200*** | ***200*** | ***200*** | ***197*** | ***170*** |
| ***Geometrical mean value*** | | | ***52*** | ***200*** | ***200*** | ***200*** | ***200*** | ***197*** | ***170*** |
| ***Standard deviation*** | | | ***4*** | ***-*** | ***-*** | ***-*** | ***-*** | ***9*** | ***40*** |
| ***Coefficient of variation*** | | | ***7*** | ***-*** | ***-*** | ***-*** | ***-*** | ***5*** | ***20*** |
| ***Median*** | | | ***52*** | ***200*** | ***200*** | ***200*** | ***200*** | ***200*** | ***200*** |
| ***Lower 90% CI of Mean*** | | | ***50*** | ***-*** | ***-*** | ***-*** | ***-*** | ***192*** | ***150*** |
| ***Upper 90% CI of Mean*** | | | ***54*** | ***-*** | ***-*** | ***-*** | ***-*** | ***200*** | ***190*** |

**Table S22.** Prothrombin time of blood plasma samples drawn during multiple injections of saline solution in rats.

| **Group #** | **Injected dose** | **Animal code** | **The time after drug injection, min** | | | | | | |
| --- | --- | --- | --- | --- | --- | --- | --- | --- | --- |
|  |  |  | **0** | **5** | **20** | **35** | **50** | **65** | **90** |
| 15 | 200 µl  ×  5 times | 127 | 12.1 | 11.8 | 13.3 | 11.4 | 11.5 | 10.7 | 10.4 |
|  |  | 128 | 13.1 | 11.6 | 12.3 | 10.6 | 9.8 | 11.4 | 13.6 |
|  |  | 129 | 10.7 | 12.6 | 13.1 | 13.6 | 11.5 | 12.9 | 10.1 |
|  |  | 130 | 12.4 | 11.5 | 11.9 | 10.8 | 13.5 | 11.4 | 12.9 |
|  |  | 131 | 10.9 | 9.7 | 11.4 | 13.1 | 10.1 | 11.9 | 13.0 |
|  |  | 132 | 12.4 | 11.6 | 13.0 | 11.6 | 11.9 | 9.1 | 12.8 |
|  |  | 133 | 12.0 | 11.3 | 10.4 | 10.7 | 9.7 | 11.8 | 10.2 |
|  |  | 134 | 13.8 | 10.6 | 10.1 | 11.8 | 12.4 | 12.9 | 11.2 |
|  |  | 135 | 10.8 | 12.4 | 9.6 | 12.3 | 12.1 | 13.6 | 12.4 |
|  |  | 136 | 11.1 | 10.2 | 12.7 | 10.6 | 12.9 | 11.6 | 12.1 |
| ***Mean value*** | | | ***11.9*** | ***11.3*** | ***11.8*** | ***11.7*** | ***11.5*** | ***11.7*** | ***11.9*** |
| ***Geometrical mean value*** | | | ***11.9*** | ***11.3*** | ***11.7*** | ***11.6*** | ***11.5*** | ***11.7*** | ***11.8*** |
| ***Standard deviation*** | | | ***1.0*** | ***0.9*** | ***1.3*** | ***1.1*** | ***1.3*** | ***1.3*** | ***1.3*** |
| ***Coefficient of variation*** | | | ***8.8*** | ***8.1*** | ***11.4*** | ***9.2*** | ***11.3*** | ***10.8*** | ***10.9*** |
| ***Median*** | | | ***12.1*** | ***11.6*** | ***12.1*** | ***11.5*** | ***11.7*** | ***11.7*** | ***12.3*** |
| ***Lower 90% CI of Mean*** | | | ***11.4*** | ***10.9*** | ***11.1*** | ***11.1*** | ***10.9*** | ***11.1*** | ***11.2*** |
| ***Upper 90% CI of Mean*** | | | ***12.5*** | ***11.8*** | ***12.5*** | ***12.2*** | ***12.2*** | ***12.4*** | ***12.5*** |

**Table S23.** Prothrombin time of blood plasma samples drawn during multiple injections of Angiox® in rats.

| **Group #** | **Injected dose** | **Animal code** | **The time after drug injection, min** | | | | | | |
| --- | --- | --- | --- | --- | --- | --- | --- | --- | --- |
|  |  |  | **0** | **7** | **15** | **30** | **45** | **65** | **90** |
| 16 | 0.19 mg/kg  ×  2 times | 137 | 11.3 | 73.2 | 38.7 | 16.9 | 77.8 | 37.3 | 15.2 |
|  |  | 138 | 12.6 | 66.6 | 35.5 | 18.0 | 73.7 | 28.3 | 17.4 |
|  |  | 139 | 10.8 | 80.3 | 54.7 | 18.3 | 44.3 | 17.7 | 11.7 |
|  |  | 140 | 10.8 | 57.9 | 30.4 | 14.0 | 58.8 | 16.9 | 12.7 |
|  |  | 141 | 10.4 | 66.8 | 32.3 | 16.0 | 51.5 | 15.5 | 12.5 |
|  |  | 142 | 9.8 | 30.3 | 13.6 | 11.3 | 16.3 | 11.9 | 11.1 |
|  |  | 143 | 10.0 | 41.5 | 21.1 | 13.3 | 25.4 | 13.6 | 11.6 |
|  |  | 144 | 10.7 | 52.3 | 22.9 | 13.7 | 23.5 | 14.8 | 12.5 |
|  |  | 145 | 10.2 | 41.8 | 17.5 | 14.2 | 39.8 | 16.7 | 13.4 |
|  |  | 146 | 11.9 | 50.6 | 21.4 | 14.7 | 32.3 | 16.4 | 13.5 |
| ***Mean value*** | | | ***10.9*** | ***56*** | ***29*** | ***15*** | ***40*** | ***19*** | ***13.2*** |
| ***Geometrical mean value*** | | | ***10.8*** | ***54*** | ***27*** | ***15*** | ***40*** | ***18*** | ***13.0*** |
| ***Standard deviation*** | | | ***0.9*** | ***16*** | ***12*** | ***2*** | ***20*** | ***8*** | ***1.9*** |
| ***Coefficient of variation*** | | | ***8.0*** | ***28*** | ***42*** | ***15*** | ***50*** | ***41*** | ***14.4*** |
| ***Median*** | | | ***10.8*** | ***55*** | ***27*** | ***15*** | ***40*** | ***17*** | ***12.6*** |
| ***Lower 90% CI of Mean*** | | | ***10.4*** | ***48*** | ***23*** | ***14*** | ***30*** | ***15*** | ***12.2*** |
| ***Upper 90% CI of Mean*** | | | ***11.3*** | ***64*** | ***35*** | ***16*** | ***60*** | ***23*** | ***14.1*** |
| 17 | 0.38 mg/kg  ×  2 times | 147 | 10.3 | 74.7 | 41.4 | 14.8 | 71.2 | 36.4 | 18.8 |
|  |  | 148 | 9.5 | 200 | 76.3 | 75.7 | 200 | 80.1 | 65.9 |
|  |  | 149 | 11.6 | 200 | 72.5 | 35.5 | 200 | 65.7 | 42.1 |
|  |  | 150 | 10.0 | 71.9 | 42.8 | 16.8 | 65.5 | 20.6 | 13.1 |
|  |  | 151 | 11.9 | 78.7 | 38.7 | 18.2 | 44.0 | 19.9 | 15.5 |
|  |  | 152 | 10.5 | 84.3 | 55.1 | 18.3 | 73.5 | 16.6 | 14.5 |
|  |  | 153 | 10.3 | 67.9 | 30.7 | 17.2 | 68.3 | 21.4 | 15.3 |
|  |  | 154 | 11.3 | 77.7 | 57.4 | 24.8 | 74.2 | 31.8 | 18.2 |
|  |  | 155 | 10.9 | 75.9 | 43.6 | 17.3 | 71.6 | 29.1 | 17.2 |
|  |  | 156 | 10.5 | 74.1 | 33.9 | 15.0 | 76.2 | 34.7 | 42.5 |
| ***Mean value*** | | | ***10.7*** | ***100*** | ***49*** | ***25*** | ***90*** | ***40*** | ***26*** |
| ***Geometrical mean value*** | | | ***10.7*** | ***90*** | ***47*** | ***21*** | ***80*** | ***30*** | ***22*** |
| ***Standard deviation*** | | | ***0.7*** | ***50*** | ***16*** | ***19*** | ***60*** | ***20*** | ***18*** |
| ***Coefficient of variation*** | | | ***7.0*** | ***50*** | ***32*** | ***74*** | ***60*** | ***60*** | ***68*** |
| ***Median*** | | | ***10.5*** | ***80*** | ***43*** | ***18*** | ***70*** | ***30*** | ***18*** |
| ***Lower 90% CI of Mean*** | | | ***10.3*** | ***70*** | ***41*** | ***16*** | ***70*** | ***20*** | ***17*** |
| ***Upper 90% CI of Mean*** | | | ***11.1*** | ***130*** | ***57*** | ***35*** | ***120*** | ***50*** | ***36*** |
| 18 | 0.75 mg/kg  ×  2 times | 157 | 12.6 | 200 | 78.1 | 38.4 | 200 | 81.5 | 49.5 |
|  |  | 158 | 11.4 | 200 | 73.6 | 29.0 | 80.3 | 47.4 | 17.5 |
|  |  | 159 | 9.4 | 200 | 68.7 | 26.8 | 200 | 59.4 | 20.6 |
|  |  | 160 | 10.3 | 200 | 77.8 | 39.3 | 77.2 | 42.7 | 16.3 |
|  |  | 161 | 11.9 | 200 | 72.9 | 27.3 | 83.0 | 32.2 | 17.0 |
|  |  | 162 | 11.4 | 200 | 200 | 47.6 | 200 | 200 | 71.1 |
|  |  | 163 | 11.9 | 200 | 77.6 | 48.3 | 86.1 | 44.8 | 17.6 |
|  |  | 164 | 10.8 | 83.7 | 73.4 | 41.3 | 200 | 69.8 | 37.5 |
|  |  | 165 | 10.2 | 200 | 78.5 | 33.9 | 200 | 50.2 | 19.5 |
|  |  | 166 | 10.5 | 200 | 70.7 | 28.7 | 77.1 | 50.1 | 17.5 |
| ***Mean value*** | | | ***11.0*** | ***190*** | ***90*** | ***36.1*** | ***140*** | ***70*** | ***28*** |
| ***Geometrical mean value*** | | | ***11.0*** | ***180*** | ***80*** | ***35.2*** | ***130*** | ***60*** | ***25*** |
| ***Standard deviation*** | | | ***1.0*** | ***40*** | ***40*** | ***8.1*** | ***60*** | ***50*** | ***19*** |
| ***Coefficient of variation*** | | | ***8.8*** | ***20*** | ***50*** | ***22.6*** | ***40*** | ***70*** | ***66*** |
| ***Median*** | | | ***11.1*** | ***200*** | ***80*** | ***36.2*** | ***140*** | ***50*** | ***19*** |
| ***Lower 90% CI of Mean*** | | | ***10.5*** | ***160*** | ***60*** | ***31.8*** | ***100*** | ***30*** | ***19*** |
| ***Upper 90% CI of Mean*** | | | ***11.5*** | ***210*** | ***120*** | ***40.3*** | ***190*** | ***100*** | ***38*** |

**Table S24.** Prothrombin time of blood plasma samples drawn during multiple injections of Thrombiveb® in rats.

| **Group #** | **Injected dose** | **Animal code** | **The time after drug injection, min** | | | | | | |
| --- | --- | --- | --- | --- | --- | --- | --- | --- | --- |
|  |  |  | **0** | **5** | **20** | **35** | **50** | **65** | **90** |
| 19 | 7.0 mg/kg  ×  5 times | 167 | 10.9 | 13.7 | 15.7 | 15.0 | 17.1 | 18.6 | 12.0 |
|  |  | 168 | 10.5 | 14.0 | 14.7 | 14.7 | 22.7 | 19.5 | 10.8 |
|  |  | 169 | 10.4 | 14.9 | 14.9 | 15.1 | 16.1 | 16.1 | 10.5 |
|  |  | 170 | 11.2 | 13.4 | 13.4 | 14.4 | 14.7 | 14.4 | 11.8 |
|  |  | 171 | 11.4 | 15.0 | 17.1 | 16.7 | 20.9 | 23.1 | 12.4 |
|  |  | 172 | 11.2 | 16.3 | 13.7 | 15.0 | 15.3 | 19.2 | 11.8 |
|  |  | 173 | 10.9 | 14.4 | 13.0 | 15.3 | 15.7 | 18.9 | 11.2 |
|  |  | 174 | 11.0 | 13.8 | 14.4 | 15.6 | 17.6 | 17.2 | 11.4 |
|  |  | 175 | 10.8 | 12.4 | 12.8 | 13.2 | 13.5 | 14.8 | 10.6 |
|  |  | 176 | 10.9 | 12.5 | 13.7 | 13.4 | 12.5 | 14.2 | 10.4 |
| ***Mean value*** | | | ***10.9*** | ***14.0*** | ***14.3*** | ***14.8*** | ***17*** | ***18*** | ***11.3*** |
| ***Geometrical mean value*** | | | ***10.9*** | ***14.0*** | ***14.3*** | ***14.8*** | ***16*** | ***17*** | ***11.3*** |
| ***Standard deviation*** | | | ***0.3*** | ***1.2*** | ***1.3*** | ***1.0*** | ***3*** | ***3*** | ***0.7*** |
| ***Coefficient of variation*** | | | ***2.8*** | ***8.4*** | ***9.2*** | ***6.9*** | ***19*** | ***16*** | ***6.2*** |
| ***Median*** | | | ***10.9*** | ***13.9*** | ***14.1*** | ***15.0*** | ***16*** | ***18*** | ***11.3*** |
| ***Lower 90% CI of Mean*** | | | ***10.8*** | ***13.4*** | ***13.7*** | ***14.3*** | ***15*** | ***16*** | ***10.9*** |
| ***Upper 90% CI of Mean*** | | | ***11.1*** | ***14.7*** | ***15.0*** | ***15.4*** | ***18*** | ***19*** | ***11.7*** |
| 20 | 21 mg/kg ×  5 times | 177 | 10.4 | 30.7 | 39.9 | 27.2 | 32.3 | 29.8 | 10.6 |
|  |  | 178 | 10.6 | 37.5 | 43.4 | 42.3 | 47.2 | 54.0 | 10.5 |
|  |  | 179 | 10.7 | 22.5 | 28.7 | 29.0 | 35.4 | 32.7 | 10.9 |
|  |  | 180 | 10.3 | 24.8 | 20.8 | 33.0 | 42.2 | 49.4 | 10.7 |
|  |  | 181 | 10.3 | 14.7 | 21.8 | 26.4 | 28.7 | 33.1 | 10.9 |
|  |  | 182 | 10.0 | 13.4 | 14.7 | 13.0 | 15.6 | 14.7 | 11.0 |
|  |  | 183 | 10.3 | 14.4 | 26.0 | 36.9 | 33.2 | 30.7 | 12.8 |
|  |  | 184 | 9.9 | 16.4 | 17.3 | 32.9 | 30.2 | 31.5 | 13.5 |
|  |  | 185 | 9.8 | 12.6 | 13.2 | 16.1 | 32.6 | 32.7 | 12.0 |
|  |  | 186 | 10.2 | 13.1 | 12.7 | 12.8 | 13.9 | 14.5 | 10.9 |
| ***Mean value*** | | | ***10.3*** | ***20*** | ***24*** | ***27*** | ***31*** | ***32*** | ***11.4*** |
| ***Geometrical mean value*** | | | ***10.2*** | ***19*** | ***22*** | ***25*** | ***29*** | ***30*** | ***11.3*** |
| ***Standard deviation*** | | | ***0.3*** | ***9*** | ***11*** | ***10*** | ***10*** | ***13*** | ***1.0*** |
| ***Coefficient of variation*** | | | ***2.8*** | ***43*** | ***45*** | ***38*** | ***33*** | ***39*** | ***9.1*** |
| ***Median*** | | | ***10.3*** | ***16*** | ***21*** | ***28*** | ***33*** | ***32*** | ***10.9*** |
| ***Lower 90% CI of Mean*** | | | ***10.1*** | ***16*** | ***18*** | ***22*** | ***26*** | ***26*** | ***10.8*** |
| ***Upper 90% CI of Mean*** | | | ***10.4*** | ***25*** | ***30*** | ***32*** | ***37*** | ***39*** | ***11.9*** |
| 21 | 42 mg/kg ×  5 times | 187 | 9.6 | 23.8 | 23.2 | 27.5 | 34.1 | 35.6 | 15.6 |
|  |  | 188 | 9.8 | 21.6 | 25.8 | 24.8 | 19.5 | 25.6 | 15.5 |
|  |  | 189 | 9.5 | 27.5 | 22.9 | 32.8 | 45.3 | 48.2 | 14.3 |
|  |  | 190 | 10.5 | 27.9 | 32.2 | 31.5 | 39.0 | 40.5 | 17.0 |
|  |  | 191 | 10.2 | 23.2 | 24.1 | 24.8 | 27.0 | 26.9 | 12.8 |
|  |  | 192 | 10.5 | 16.0 | 26.5 | 26.8 | 34.1 | 52.1 | 22.2 |
|  |  | 193 | 10.1 | 28.9 | 24.3 | 28.4 | 30.4 | 40.1 | 14.0 |
|  |  | 194 | 11.5 | 24.9 | 36.4 | 38.0 | 34.2 | 31.2 | 17.6 |
|  |  | 195 | 9.2 | 22.8 | 32.9 | 34.8 | 41.3 | 45.9 | 17.3 |
|  |  | 196 | 10.9 | 22.9 | 34.6 | 30.2 | 32.1 | 33.8 | 16.9 |
| ***Mean value*** | | | ***10.2*** | ***24*** | ***28*** | ***30*** | ***34*** | ***38*** | ***16*** |
| ***Geometrical mean value*** | | | ***10.2*** | ***24*** | ***28*** | ***30*** | ***33*** | ***37*** | ***16*** |
| ***Standard deviation*** | | | ***0.7*** | ***4*** | ***5*** | ***4*** | ***7*** | ***9*** | ***3*** |
| ***Coefficient of variation*** | | | ***6.8*** | ***16*** | ***18*** | ***15*** | ***22*** | ***24*** | ***16*** |
| ***Median*** | | | ***10.2*** | ***24*** | ***26*** | ***29*** | ***34*** | ***38*** | ***16*** |
| ***Lower 90% CI of Mean*** | | | ***9.8*** | ***22*** | ***26*** | ***28*** | ***30*** | ***33*** | ***15*** |
| ***Upper 90% CI of Mean*** | | | ***10.5*** | ***26*** | ***31*** | ***32*** | ***38*** | ***43*** | ***18*** |

**Table S25.** APTT of blood plasma samples drawn during multiple injections of saline solution in rats.

| **Group #** | **Injected dose** | **Animal code** | **The time after drug injection, min** | | | | | | |
| --- | --- | --- | --- | --- | --- | --- | --- | --- | --- |
|  |  |  | **0** | **5** | **20** | **35** | **50** | **65** | **90** |
| 15 | 200 µl  ×  5 times | 127 | 21.6 | 19.1 | 20.4 | 23.5 | 20.4 | 18.1 | 24.0 |
|  |  | 128 | 19.2 | 19.6 | 21.8 | 19.2 | 20.6 | 22.3 | 18.7 |
|  |  | 129 | 19.3 | 19.6 | 23.1 | 18.9 | 19.2 | 19.4 | 19.1 |
|  |  | 130 | 23.6 | 22.1 | 21.4 | 24.1 | 23.1 | 19.5 | 22.4 |
|  |  | 131 | 19.0 | 20.7 | 22.3 | 22.6 | 19.3 | 19.9 | 22.5 |
|  |  | 132 | 18.9 | 23.4 | 19.1 | 22.0 | 18.6 | 19.3 | 24.1 |
|  |  | 133 | 19.7 | 23.6 | 21.7 | 20.0 | 21.3 | 24.0 | 22.9 |
|  |  | 134 | 19.1 | 21.6 | 19.3 | 23.0 | 19.5 | 21.8 | 19.3 |
|  |  | 135 | 22.6 | 24.1 | 18.9 | 22.3 | 20.0 | 19.3 | 24.1 |
|  |  | 136 | 22.7 | 21.9 | 23.5 | 21.4 | 24.2 | 18.9 | 19.2 |
| ***Mean value*** | | | ***20.6*** | ***21.6*** | ***21.2*** | ***21.7*** | ***20.6*** | ***20.3*** | ***22*** |
| ***Geometrical mean value*** | | | ***20.5*** | ***21.5*** | ***21.1*** | ***21.6*** | ***20.6*** | ***20.2*** | ***22*** |
| ***Standard deviation*** | | | ***1.8*** | ***1.8*** | ***1.7*** | ***1.8*** | ***1.8*** | ***1.8*** | ***2*** |
| ***Coefficient of variation*** | | | ***9.0*** | ***8.3*** | ***7.8*** | ***8.3*** | ***8.7*** | ***9.1*** | ***11*** |
| ***Median*** | | | ***19.5*** | ***21.8*** | ***21.6*** | ***22.2*** | ***20.2*** | ***19.5*** | ***23*** |
| ***Lower 90% CI of Mean*** | | | ***19.6*** | ***20.6*** | ***20.3*** | ***20.8*** | ***19.7*** | ***19.3*** | ***20*** |
| ***Upper 90% CI of Mean*** | | | ***21.5*** | ***22.5*** | ***22.0*** | ***22.6*** | ***21.6*** | ***21.2*** | ***23*** |

**Table S26.** APTT of blood plasma samples drawn during multiple injections of Angiox® in rats.

| **Group #** | **Injected dose** | **Animal code** | **The time after drug injection, min** | | | | | | |
| --- | --- | --- | --- | --- | --- | --- | --- | --- | --- |
|  |  |  | **0** | **7** | **15** | **30** | **45** | **65** | **90** |
| 16 | 0.19 mg/kg  ×  2 times | 137 | 27.5 | 124.7 | 78.4 | 57.5 | 131.3 | 80.9 | 40.4 |
|  |  | 138 | 31.0 | 135.1 | 130.9 | 84.5 | 138.1 | 101.2 | 88.9 |
|  |  | 139 | 20.4 | 138.4 | 91.9 | 56.2 | 83.4 | 63.6 | 40.5 |
|  |  | 140 | 21.0 | 97.9 | 82.6 | 49.0 | 103.2 | 60.0 | 45.9 |
|  |  | 141 | 21.9 | 122.3 | 71.0 | 66.4 | 117.9 | 50.0 | 38.2 |
|  |  | 142 | 23.4 | 73.5 | 56.5 | 42.2 | 62.0 | 49.5 | 31.0 |
|  |  | 143 | 20.8 | 91.7 | 60.8 | 57.2 | 65.5 | 51.5 | 44.9 |
|  |  | 144 | 21.0 | 91.7 | 88.1 | 54.9 | 69.9 | 64.9 | 43.2 |
|  |  | 145 | 24.5 | 102.7 | 70.6 | 55.2 | 151.1 | 64.4 | 49.9 |
|  |  | 146 | 23.4 | 114.8 | 58.3 | 40.6 | 74.0 | 48.6 | 35.6 |
| ***Mean value*** | | | ***24*** | ***110*** | ***80*** | ***56*** | ***100*** | ***64*** | ***46*** |
| ***Geometrical mean value*** | | | ***23*** | ***110*** | ***80*** | ***55*** | ***90*** | ***62*** | ***44.*** |
| ***Standard deviation*** | | | ***3*** | ***20*** | ***20*** | ***13*** | ***30*** | ***17*** | ***16*** |
| ***Coefficient of variation*** | | | ***15*** | ***20*** | ***30*** | ***22*** | ***30*** | ***26*** | ***35*** |
| ***Median*** | | | ***23*** | ***110*** | ***70*** | ***56*** | ***90*** | ***62*** | ***42*** |
| ***Lower 90% CI of Mean*** | | | ***22*** | ***100*** | ***70*** | ***50*** | ***80*** | ***55*** | ***38*** |
| ***Upper 90% CI of Mean*** | | | ***25*** | ***120*** | ***90*** | ***63*** | ***120*** | ***72*** | ***54*** |
| 17 | 0.38 mg/kg  ×  2 times | 147 | 23.0 | 138.6 | 136.2 | 81.7 | 141.2 | 108.0 | 70.8 |
|  |  | 148 | 22.5 | 175.8 | 142.1 | 140.3 | 200 | 154.2 | 118.1 |
|  |  | 149 | 34.5 | 200 | 174.4 | 119.2 | 200 | 174.9 | 104.3 |
|  |  | 150 | 25.1 | 136.8 | 103.4 | 70.3 | 117 | 68.4 | 47.8 |
|  |  | 151 | 25.7 | 124.3 | 76.7 | 64.3 | 110.9 | 70.7 | 56.1 |
|  |  | 152 | 19.5 | 152.7 | 79.6 | 52.7 | 115.3 | 45.2 | 50.6 |
|  |  | 153 | 20.6 | 116.1 | 64.9 | 50.7 | 102.3 | 55.8 | 44.9 |
|  |  | 154 | 20.2 | 118.8 | 88.4 | 65.7 | 123.8 | 63.3 | 48.3 |
|  |  | 155 | 24.5 | 145.2 | 87.5 | 79.7 | 135.1 | 79.5 | 67.6 |
|  |  | 156 | 20.1 | 126.9 | 88.2 | 43.8 | 119.8 | 83.8 | 80.6 |
| ***Mean value*** | | | ***24*** | ***140*** | ***100*** | ***80*** | ***140*** | ***90*** | ***70*** |
| ***Geometrical mean value*** | | | ***23*** | ***140*** | ***1000*** | ***70*** | ***130*** | ***80*** | ***70*** |
| ***Standard deviation*** | | | ***4*** | ***30*** | ***40*** | ***30*** | ***40*** | ***40*** | ***30*** |
| ***Coefficient of variation*** | | | ***19*** | ***20*** | ***30*** | ***40*** | ***30*** | ***50*** | ***40*** |
| ***Median*** | | | ***23*** | ***140*** | ***90*** | ***70*** | ***120*** | ***80*** | ***60*** |
| ***Lower 90% CI of Mean*** | | | ***21*** | ***130*** | ***90*** | ***60*** | ***120*** | ***70*** | ***60*** |
| ***Upper 90% CI of Mean*** | | | ***26*** | ***160*** | ***120*** | ***90*** | ***150*** | ***110*** | ***80*** |
| 18 | 0.75 mg/kg  ×  2 times | 157 | 25.4 | 173.3 | 133.1 | 77.9 | 172.0 | 152.9 | 88.2 |
|  |  | 158 | 20.0 | 153.3 | 112.4 | 64.4 | 123.6 | 83.1 | 42.0 |
|  |  | 159 | 23.9 | 161.2 | 108.4 | 75.8 | 161.2 | 92.8 | 62.8 |
|  |  | 160 | 19.5 | 141.4 | 108.4 | 58.0 | 109.8 | 56.2 | 38.9 |
|  |  | 161 | 17.3 | 142.1 | 83.8 | 40.0 | 116.8 | 44.0 | 32.3 |
|  |  | 162 | 20.2 | 169.2 | 147.4 | 81.6 | 163.7 | 152.2 | 122.7 |
|  |  | 163 | 22.2 | 164.8 | 130.4 | 81.2 | 146.9 | 83.4 | 56.9 |
|  |  | 164 | 20.9 | 136.6 | 108.2 | 78.1 | 150.7 | 103.6 | 104.5 |
|  |  | 165 | 22.0 | 157.3 | 126.9 | 66.1 | 155.3 | 95.2 | 52.9 |
|  |  | 166 | 22.4 | 159.3 | 120.5 | 68.0 | 129.8 | 82.7 | 52.5 |
| ***Mean value*** | | | ***21*** | ***156*** | ***118*** | ***69*** | ***140*** | ***100*** | ***70*** |
| ***Geometrical mean value*** | | | ***21*** | ***155*** | ***117*** | ***68*** | ***140*** | ***90*** | ***60*** |
| ***Standard deviation*** | | | ***2*** | ***12*** | ***18*** | ***13*** | ***20*** | ***40*** | ***30*** |
| ***Coefficient of variation*** | | | ***11*** | ***8*** | ***15*** | ***19*** | ***20*** | ***40*** | ***50*** |
| ***Median*** | | | ***22*** | ***158*** | ***117*** | ***72*** | ***150*** | ***90*** | ***50*** |
| ***Lower 90% CI of Mean*** | | | ***20*** | ***149*** | ***109*** | ***62*** | ***130*** | ***80*** | ***50*** |
| ***Upper 90% CI of Mean*** | | | ***23*** | ***162*** | ***127*** | ***76*** | ***150*** | ***110*** | ***80*** |

**Table S27.** APTT of blood plasma samples drawn during multiple injections of Thrombiveb® in rats.

| **Group #** | **Injected dose** | **Animal code** | **The time after drug injection, min** | | | | | | |
| --- | --- | --- | --- | --- | --- | --- | --- | --- | --- |
|  |  |  | **0** | **5** | **20** | **35** | **50** | **65** | **90** |
| 19 | 7.0 mg/kg  ×  5 times | 167 | 16.6 | 24.1 | 26.5 | 29.7 | 32.1 | 34.7 | 20.9 |
|  |  | 168 | 27.3 | 36.8 | 39.7 | 42.0 | 55.5 | 49.3 | 27.4 |
|  |  | 169 | 20.1 | 29.8 | 33.2 | 34.2 | 36.0 | 35.2 | 24.3 |
|  |  | 170 | 21.0 | 30.9 | 28.6 | 35.7 | 31.6 | 32.2 | 22.8 |
|  |  | 171 | 23.1 | 33.9 | 38.5 | 37.5 | 46.1 | 50.7 | 29.1 |
|  |  | 172 | 16.9 | 25.5 | 24.8 | 24.6 | 27.6 | 31.0 | 21.8 |
|  |  | 173 | 19.9 | 28.1 | 27.4 | 32.7 | 34.0 | 42.8 | 22.5 |
|  |  | 174 | 22.4 | 28.7 | 31.3 | 36.8 | 39.6 | 38.5 | 27.7 |
|  |  | 175 | 22.9 | 30.9 | 34.1 | 35.2 | 43.2 | 42.2 | 27.2 |
|  |  | 176 | 22.6 | 30.3 | 33.7 | 32.7 | 33.2 | 53.7 | 31.3 |
| ***Mean value*** | | | ***21*** | ***30*** | ***32*** | ***34*** | ***38*** | ***41*** | ***26*** |
| ***Geometrical mean value*** | | | ***21*** | ***30*** | ***31*** | ***34*** | ***37*** | ***40*** | ***25*** |
| ***Standard deviation*** | | | ***3*** | ***4*** | ***5*** | ***5*** | ***8*** | ***8*** | ***4*** |
| ***Coefficient of variation*** | | | ***15*** | ***12*** | ***16*** | ***14*** | ***22*** | ***20*** | ***14*** |
| ***Median*** | | | ***22*** | ***30*** | ***32*** | ***35*** | ***35*** | ***40*** | ***26*** |
| ***Lower 90% CI of Mean*** | | | ***20*** | ***28*** | ***29*** | ***32*** | ***34*** | ***37*** | ***24*** |
| ***Upper 90% CI of Mean*** | | | ***23*** | ***32*** | ***34*** | ***37*** | ***42*** | ***45*** | ***27*** |
| 20 | 21 mg/kg ×  5 times | 177 | 43.5 | 78.7 | 87.8 | 89.7 | 88.2 | 68.4 | 30.9 |
|  |  | 178 | 29.6 | 107.6 | 114.2 | 131.8 | 183.0 | 200 | 176.2 |
|  |  | 179 | 21.4 | 73.9 | 66.6 | 66.6 | 89.3 | 79.6 | 34.0 |
|  |  | 180 | 22.4 | 49.5 | 57.6 | 83.6 | 109.5 | 200 | 182 |
|  |  | 181 | 30.0 | 68.3 | 52.8 | 74.7 | 64.0 | 70.2 | 33.0 |
|  |  | 182 | 22.8 | 50.1 | 44.4 | 53.4 | 67.0 | 63.7 | 67.4 |
|  |  | 183 | 20.4 | 56.6 | 85.1 | 74.6 | 68.2 | 65.4 | 62.1 |
|  |  | 184 | 58.9 | 86.9 | 88.2 | 137 | 158.8 | 200 | 134.0 |
|  |  | 185 | 26.1 | 39.9 | 56.4 | 70.8 | 130.4 | 117.0 | 121.2 |
|  |  | 186 | 22.1 | 48.7 | 73.2 | 96.1 | 124.1 | 107.6 | 91.7 |
| ***Mean value*** | | | ***30*** | ***70*** | ***70*** | ***90*** | ***110*** | ***120*** | ***90*** |
| ***Geometrical mean value*** | | | ***28*** | ***60*** | ***70*** | ***80*** | ***100*** | ***100*** | ***80*** |
| ***Standard deviation*** | | | ***12*** | ***20*** | ***20*** | ***30*** | ***40*** | ***60*** | ***60*** |
| ***Coefficient of variation*** | | | ***42*** | ***30*** | ***30*** | ***30*** | ***40*** | ***50*** | ***60*** |
| ***Median*** | | | ***25*** | ***60*** | ***70*** | ***80*** | ***100*** | ***90*** | ***80*** |
| ***Lower 90% CI of Mean*** | | | ***23*** | ***60*** | ***60*** | ***70*** | ***90*** | ***90*** | ***60*** |
| ***Upper 90% CI of Mean*** | | | ***36*** | ***80*** | ***80*** | ***100*** | ***130*** | ***150*** | ***120*** |
| 21 | 42 mg/kg ×  5 times | 187 | 16.5 | 63.5 | 69.7 | 105.4 | 152.2 | 148.5 | 77.0 |
|  |  | 188 | 19.5 | 93.9 | 97.7 | 102.9 | 165 | 120.8 | 85.1 |
|  |  | 189 | 29.5 | 89.5 | 114.4 | 200 | 200 | 200 | 181.9 |
|  |  | 190 | 21.1 | 75.0 | 96.6 | 96.6 | 153.1 | 200 | 84.0 |
|  |  | 191 | 15.7 | 49.9 | 80.9 | 69.1 | 82.9 | 88.9 | 57.5 |
|  |  | 192 | 24.7 | 91.5 | 88.2 | 159.9 | 187.4 | 200 | 160.8 |
|  |  | 193 | 23.6 | 85.1 | 82.7 | 134.0 | 108.7 | 200 | 85.1 |
|  |  | 194 | 40.8 | 68.5 | 128.0 | 119.4 | 171.8 | 102.3 | 88.6 |
|  |  | 195 | 19.2 | 64.9 | 88.6 | 114.4 | 156.2 | 200 | 128.0 |
|  |  | 196 | 27.3 | 89.7 | 110.2 | 152.9 | 99.9 | 153.1 | 89.1 |
| ***Mean value*** | | | ***24*** | ***77*** | ***96*** | ***130*** | ***150*** | ***160*** | ***100*** |
| ***Geometrical mean value*** | | | ***23*** | ***76*** | ***94*** | ***120*** | ***140*** | ***160*** | ***100*** |
| ***Standard deviation*** | | | ***8*** | ***15*** | ***18*** | ***40*** | ***40*** | ***40*** | ***40*** |
| ***Coefficient of variation*** | | | ***31*** | ***19*** | ***18*** | ***30*** | ***30*** | ***30*** | ***40*** |
| ***Median*** | | | ***22*** | ***80*** | ***93*** | ***120*** | ***150*** | ***180*** | ***90*** |
| ***Lower 90% CI of Mean*** | | | ***20*** | ***69*** | ***87*** | ***100*** | ***130*** | ***140*** | ***80*** |
| ***Upper 90% CI of Mean*** | | | ***28*** | ***85*** | ***105*** | ***150*** | ***170*** | ***180*** | ***120*** |

**Table S28.** The effect of single bolus injection of saline solution on thrombus growth in the model of induced thrombosis.

| **Time, min** | **Thrombus area, mm^2^** | | | | | | | | | | **Mean value** | **St. dev.** |
| --- | --- | --- | --- | --- | --- | --- | --- | --- | --- | --- | --- | --- |
|  | *197* | *198* | *199* | *200* | *201* | *202* | *203* | *204* | *205* | *206* |  |  |
| 3 | 0.13 | 0.21 | 0.22 | 0.22 | 0.38 | 0.24 | 0.18 | 0.12 | 0.21 | 0.21 | 0.21 | 0.07 |
| 4 | 0.13 | 0.27 | 0.20 | 0.32 | 0.37 | 0.26 | 0.21 | 0.19 | 0.24 | 0.22 | 0.24 | 0.07 |
| 5 | 0.10 | 0.23 | 0.18 | 0.27 | 0.40 | 0.29 | 0.19 | 0.22 | 0.27 | 0.27 | 0.24 | 0.08 |
| 6 | 0.09 | 0.24 | 0.31 | 0.30 | 0.36 | 0.33 | 0.18 | 0.48 | 0.28 | 0.25 | 0.28 | 0.11 |
| 7 | 0.12 | 0.28 | 0.34 | 0.30 | 0.38 | 0.28 | 0.24 | 0.68 | 0.28 | 0.22 | 0.31 | 0.15 |
| 8 | 0.19 | 0.26 | 0.37 | 0.32 | 0.35 | 0.24 | 0.36 | 0.69 | 0.28 | 0.24 | 0.33 | 0.14 |
| 9 | 0.36 | 0.29 | 0.40 | 0.27 | 0.35 | 0.21 | 0.49 | 0.66 | 0.29 | 0.23 | 0.36 | 0.14 |
| 10 | 0.38 | 0.29 | 0.55 | 0.34 | 0.35 | 0.23 | 0.50 | 0.70 | 0.31 | 0.24 | 0.39 | 0.15 |
| 11 | 0.36 | 0.39 | 0.52 | 0.40 | 0.38 | 0.33 | 0.34 | 0.71 | 0.31 | 0.23 | 0.40 | 0.13 |
| 12 | 0.58 | 0.50 | 0.55 | 0.33 | 0.45 | 0.32 | 0.53 | 0.66 | 0.31 | 0.29 | 0.45 | 0.13 |
| 13 | 0.49 | 0.35 | 0.51 | 0.44 | 0.43 | 0.37 | 0.47 | 0.78 | 0.42 | 0.30 | 0.46 | 0.13 |
| 14 | 0.49 | 0.37 | 0.58 | 0.44 | 0.41 | 0.35 | 0.45 | 0.72 | 0.34 | 0.39 | 0.45 | 0.12 |
| 15 | 0.46 | 0.37 | 0.59 | 0.45 | 0.44 | 0.33 | 0.44 | 0.65 | 0.61 | 0.34 | 0.47 | 0.11 |
| 16 | 0.62 | 0.36 | 0.59 | 0.48 | 0.45 | 0.36 | 0.56 | 0.74 | 0.79 | 0.40 | 0.54 | 0.15 |
| 17 | 0.53 | 0.36 | 0.61 | 0.42 | 0.47 | 0.34 | 0.40 | 0.63 | 0.83 | 0.46 | 0.50 | 0.15 |
| 18 | 0.69 | 0.36 | 0.61 | 0.44 | 0.46 | 0.39 | 0.65 | 0.72 | 0.78 | 0.49 | 0.56 | 0.15 |
| 19 | 0.68 | 0.40 | 0.66 | 0.45 | 0.47 | 0.42 | 0.53 | 0.41 | 0.84 | 0.51 | 0.54 | 0.14 |
| 20 | 0.66 | 0.40 | 0.63 | 0.46 | 0.47 | 0.33 | 0.69 | 0.43 | 0.85 | 0.49 | 0.54 | 0.16 |
| 21 | 0.66 | 0.41 | 0.67 | 0.45 | 0.55 | 0.39 | 0.49 | 0.52 | 0.87 | 0.52 | 0.55 | 0.14 |
| 22 | 0.70 | 0.42 | 0.56 | 0.46 | 0.66 | 0.37 | 0.58 | 0.33 | 0.91 | 0.48 | 0.55 | 0.18 |
| 23 | 0.57 | 0.42 | 0.66 | 0.47 | 0.61 | 0.38 | 0.64 | 0.45 | 1.04 | 0.50 | 0.57 | 0.19 |
| 24 | 0.59 | 0.41 | 0.69 | 0.62 | 0.67 | 0.41 | 0.70 | 0.51 | 0.94 | 0.53 | 0.61 | 0.16 |
| 25 | 0.48 | 0.41 | 0.69 | 0.73 | 0.66 | 0.36 | 0.49 | 0.48 | 1.05 | 0.56 | 0.59 | 0.20 |
| 26 | 0.50 | 0.39 | 0.69 | 0.75 | 0.68 | 0.32 | 0.58 | 0.40 | 1.03 | 0.52 | 0.60 | 0.20 |
| 27 | 0.48 | 0.42 | 0.73 | 0.84 | 0.63 | 0.32 | 0.59 | 0.51 | 0.99 | 0.59 | 0.61 | 0.20 |
| 28 | 0.56 | 0.43 | 0.67 | 0.94 | 0.67 | 0.29 | 0.60 | 0.42 | 0.98 | 0.58 | 0.60 | 0.20 |
| 29 | 0.58 | 0.45 | 0.70 | 0.87 | 0.74 | 0.36 | 0.57 | 0.43 | 0.95 | 0.59 | 0.62 | 0.19 |
| 30 | 0.63 | 0.46 | 0.64 | 0.90 | 0.68 | 0.36 | 0.69 | 0.49 | 0.98 | 0.66 | 0.65 | 0.19 |
| 31 | 0.66 | 0.47 | 0.73 | 0.86 | 0.76 | 0.37 | 0.65 | 0.44 | 0.91 | 0.49 | 0.63 | 0.19 |
| 32 | 0.61 | 0.52 | 0.82 | 0.84 | 0.66 | 0.38 | 0.58 | 0.47 | 0.95 | 0.49 | 0.63 | 0.18 |
| 33 | 0.49 | 0.48 | 0.81 | 0.92 | 0.70 | 0.39 | 0.46 | 0.42 | 0.92 | 0.51 | 0.60 | 0.20 |
| 34 | 0.66 | 0.50 | 0.76 | 0.81 | 0.64 | 0.39 | 0.66 | 0.44 | 0.88 | 0.55 | 0.63 | 0.16 |
| 35 | 0.63 | 0.46 | 0.83 | 0.93 | 0.66 | 0.36 | 0.74 | 0.44 | 0.89 | 0.59 | 0.65 | 0.20 |
| 36 | 0.59 | 0.53 | 0.74 | 0.94 | 0.63 | 0.36 | 0.64 | 0.43 | 0.89 | 0.61 | 0.64 | 0.18 |
| 37 | 0.57 | 0.53 | 0.69 | 0.96 | 0.73 | 0.38 | 0.66 | 0.43 | 0.94 | 0.61 | 0.65 | 0.19 |
| 38 | 0.45 | 0.57 | 0.68 | 0.85 | 0.72 | 0.38 | 0.46 | 0.43 | 0.90 | 0.60 | 0.60 | 0.18 |
| 39 | 0.57 | 0.57 | 0.66 | 0.79 | 0.73 | 0.37 | 0.50 | 0.43 | 0.92 | 0.60 | 0.61 | 0.17 |
| 40 | 0.44 | 0.55 | 0.66 | 0.94 | 0.68 | 0.39 | 0.52 | 0.42 | 0.93 | 0.55 | 0.61 | 0.20 |

**Table S29.** The effect of single bolus injection of bivalirudin in 0.19 mg/kg dose on thrombus growth in the model of induced thrombosis.

| **Time, min** | **Thrombus area, mm^2^** | | | | | | **Mean value** | **St. dev.** |
| --- | --- | --- | --- | --- | --- | --- | --- | --- |
|  | *207* | *208* | *209* | *210* | *211* | *212* |  |  |
| 3 | 0.18 | 0.29 | 0.21 | 0.15 | 0.32 | 0.36 | 0.25 | 0.08 |
| 4 | 0.18 | 0.28 | 0.19 | 0.17 | 0.36 | 0.39 | 0.26 | 0.10 |
| 5 | 0.18 | 0.26 | 0.17 | 0.23 | 0.37 | 0.41 | 0.27 | 0.10 |
| 6 | 0.24 | 0.29 | 0.20 | 0.15 | 0.35 | 0.39 | 0.27 | 0.09 |
| 7 | 0.32 | 0.29 | 0.20 | 0.20 | 0.36 | 0.43 | 0.30 | 0.09 |
| 8 | 0.35 | 0.30 | 0.20 | 0.14 | 0.36 | 0.49 | 0.31 | 0.12 |
| 9 | 0.49 | 0.32 | 0.20 | 0.20 | 0.38 | 0.43 | 0.34 | 0.12 |
| 10 | 0.48 | 0.31 | 0.22 | 0.24 | 0.40 | 0.41 | 0.34 | 0.10 |
| 11 | 0.49 | 0.28 | 0.20 | 0.23 | 0.39 | 0.41 | 0.33 | 0.11 |
| 12 | 0.49 | 0.29 | 0.21 | 0.22 | 0.48 | 0.40 | 0.35 | 0.12 |
| 13 | 0.48 | 0.31 | 0.26 | 0.22 | 0.53 | 0.40 | 0.37 | 0.12 |
| 14 | 0.53 | 0.28 | 0.24 | 0.22 | 0.62 | 0.48 | 0.40 | 0.17 |
| 15 | 0.48 | 0.30 | 0.24 | 0.23 | 0.60 | 0.44 | 0.38 | 0.15 |
| 16 | 0.51 | 0.27 | 0.21 | 0.27 | 0.70 | 0.46 | 0.40 | 0.19 |
| 17 | 0.49 | 0.29 | 0.27 | 0.26 | 0.72 | 0.49 | 0.42 | 0.18 |
| 18 | 0.47 | 0.30 | 0.27 | 0.24 | 0.66 | 0.51 | 0.41 | 0.17 |
| 19 | 0.52 | 0.29 | 0.34 | 0.22 | 0.69 | 0.52 | 0.43 | 0.17 |
| 20 | 0.42 | 0.30 | 0.35 | 0.25 | 0.70 | 0.49 | 0.42 | 0.16 |
| 21 | 0.45 | 0.27 | 0.27 | 0.25 | 0.62 | 0.52 | 0.40 | 0.15 |
| 22 | 0.46 | 0.28 | 0.29 | 0.26 | 0.66 | 0.56 | 0.42 | 0.17 |
| 23 | 0.40 | 0.29 | 0.36 | 0.27 | 0.58 | 0.60 | 0.42 | 0.14 |
| 24 | 0.45 | 0.29 | 0.32 | 0.30 | 0.64 | 0.62 | 0.44 | 0.16 |
| 25 | 0.55 | 0.26 | 0.30 | 0.33 | 0.60 | 0.62 | 0.45 | 0.16 |
| 26 | 0.54 | 0.28 | 0.33 | 0.29 | 0.65 | 0.59 | 0.44 | 0.16 |
| 27 | 0.50 | 0.27 | 0.36 | 0.29 | 0.60 | 0.51 | 0.42 | 0.13 |
| 28 | 0.44 | 0.28 | 0.32 | 0.28 | 0.65 | 0.54 | 0.42 | 0.15 |
| 29 | 0.50 | 0.28 | 0.30 | 0.27 | 0.65 | 0.52 | 0.42 | 0.16 |
| 30 | 0.49 | 0.26 | 0.29 | 0.26 | 0.61 | 0.44 | 0.39 | 0.14 |
| 31 | 0.51 | 0.28 | 0.30 | 0.26 | 0.67 | 0.48 | 0.42 | 0.16 |
| 32 | 0.52 | 0.27 | 0.31 | 0.26 | 0.69 | 0.50 | 0.42 | 0.17 |
| 33 | 0.46 | 0.27 | 0.28 | 0.27 | 0.66 | 0.46 | 0.40 | 0.16 |
| 34 | 0.44 | 0.28 | 0.25 | 0.26 | 0.60 | 0.54 | 0.40 | 0.15 |
| 35 | 0.41 | 0.28 | 0.30 | 0.24 | 0.63 | 0.49 | 0.39 | 0.15 |
| 36 | 0.40 | 0.27 | 0.25 | 0.22 | 0.67 | 0.43 | 0.37 | 0.17 |
| 37 | 0.37 | 0.27 | 0.30 | 0.24 | 0.68 | 0.47 | 0.39 | 0.17 |
| 38 | 0.39 | 0.27 | 0.29 | 0.20 | 0.69 | 0.47 | 0.39 | 0.18 |
| 39 | 0.44 | 0.27 | 0.28 | 0.23 | 0.67 | 0.46 | 0.39 | 0.17 |
| 40 | 0.37 | 0.28 | 0.27 | 0.26 | 0.68 | 0.45 | 0.39 | 0.16 |

**Table S30.** The effect of single bolus injection of bivalirudin in 0.38 mg/kg dose on thrombus growth in the model of induced thrombosis.

| **Time, min** | **Thrombus area, mm^2^** | | | | | | | | | | **Mean value** | **St. dev.** |
| --- | --- | --- | --- | --- | --- | --- | --- | --- | --- | --- | --- | --- |
|  | *213* | *214* | *215* | *216* | *217* | *218* | *219* | *220* | *221* | *222* |  |  |
| 3 | 0.13 | 0.10 | 0.20 | 0.30 | 0.06 | 0.07 | 0.30 | 0.19 | 0.39 | 0.24 | 0.20 | 0.11 |
| 4 | 0.12 | 0.19 | 0.22 | 0.03 | 0.16 | 0.07 | 0.22 | 0.23 | 0.42 | 0.23 | 0.19 | 0.11 |
| 5 | 0.13 | 0.13 | 0.24 | 0.23 | 0.21 | 0.22 | 0.30 | 0.26 | 0.44 | 0.21 | 0.24 | 0.09 |
| 6 | 0.16 | 0.14 | 0.29 | 0.22 | 0.17 | 0.27 | 0.30 | 0.23 | 0.39 | 0.22 | 0.24 | 0.08 |
| 7 | 0.11 | 0.23 | 0.33 | 0.24 | 0.14 | 0.35 | 0.30 | 0.29 | 0.41 | 0.17 | 0.26 | 0.10 |
| 8 | 0.14 | 0.15 | 0.33 | 0.25 | 0.14 | 0.35 | 0.35 | 0.26 | 0.40 | 0.07 | 0.24 | 0.11 |
| 9 | 0.14 | 0.12 | 0.27 | 0.23 | 0.15 | 0.31 | 0.32 | 0.34 | 0.37 | 0.24 | 0.25 | 0.09 |
| 10 | 0.16 | 0.15 | 0.27 | 0.25 | 0.18 | 0.32 | 0.41 | 0.31 | 0.39 | 0.08 | 0.25 | 0.11 |
| 11 | 0.19 | 0.15 | 0.28 | 0.27 | 0.18 | 0.32 | 0.40 | 0.35 | 0.44 | 0.16 | 0.27 | 0.10 |
| 12 | 0.15 | 0.18 | 0.26 | 0.25 | 0.22 | 0.34 | 0.45 | 0.40 | 0.40 | 0.14 | 0.28 | 0.11 |
| 13 | 0.15 | 0.16 | 0.27 | 0.24 | 0.20 | 0.33 | 0.42 | 0.39 | 0.41 | 0.17 | 0.27 | 0.11 |
| 14 | 0.16 | 0.20 | 0.37 | 0.25 | 0.22 | 0.41 | 0.43 | 0.43 | 0.43 | 0.17 | 0.31 | 0.12 |
| 15 | 0.14 | 0.21 | 0.35 | 0.25 | 0.17 | 0.36 | 0.47 | 0.42 | 0.46 | 0.15 | 0.30 | 0.13 |
| 16 | 0.20 | 0.18 | 0.36 | 0.24 | 0.24 | 0.41 | 0.45 | 0.41 | 0.43 | 0.34 | 0.32 | 0.10 |
| 17 | 0.18 | 0.25 | 0.44 | 0.27 | 0.31 | 0.40 | 0.42 | 0.44 | 0.54 | 0.32 | 0.36 | 0.11 |
| 18 | 0.27 | 0.22 | 0.43 | 0.20 | 0.39 | 0.41 | 0.47 | 0.42 | 0.52 | 0.24 | 0.36 | 0.12 |
| 19 | 0.29 | 0.33 | 0.48 | 0.23 | 0.41 | 0.42 | 0.44 | 0.44 | 0.57 | 0.23 | 0.38 | 0.11 |
| 20 | 0.25 | 0.33 | 0.52 | 0.24 | 0.49 | 0.43 | 0.41 | 0.44 | 0.54 | 0.26 | 0.39 | 0.11 |
| 21 | 0.17 | 0.28 | 0.50 | 0.28 | 0.49 | 0.42 | 0.50 | 0.45 | 0.55 | 0.27 | 0.39 | 0.13 |
| 22 | 0.17 | 0.39 | 0.52 | 0.34 | 0.49 | 0.41 | 0.45 | 0.44 | 0.53 | 0.29 | 0.40 | 0.11 |
| 23 | 0.15 | 0.35 | 0.54 | 0.30 | 0.37 | 0.37 | 0.45 | 0.49 | 0.55 | 0.28 | 0.38 | 0.13 |
| 24 | 0.17 | 0.37 | 0.56 | 0.28 | 0.45 | 0.40 | 0.45 | 0.41 | 0.58 | 0.33 | 0.40 | 0.12 |
| 25 | 0.18 | 0.32 | 0.54 | 0.28 | 0.29 | 0.40 | 0.42 | 0.44 | 0.57 | 0.37 | 0.38 | 0.12 |
| 26 | 0.17 | 0.33 | 0.54 | 0.28 | 0.37 | 0.38 | 0.44 | 0.45 | 0.68 | 0.26 | 0.39 | 0.15 |
| 27 | 0.16 | 0.30 | 0.53 | 0.29 | 0.32 | 0.39 | 0.42 | 0.57 | 0.58 | 0.26 | 0.38 | 0.14 |
| 28 | 0.33 | 0.24 | 0.65 | 0.31 | 0.29 | 0.37 | 0.38 | 0.52 | 0.58 | 0.24 | 0.39 | 0.14 |
| 29 | 0.32 | 0.32 | 0.55 | 0.29 | 0.31 | 0.41 | 0.39 | 0.44 | 0.63 | 0.27 | 0.39 | 0.12 |
| 30 | 0.29 | 0.27 | 0.58 | 0.24 | 0.26 | 0.46 | 0.38 | 0.44 | 0.65 | 0.32 | 0.39 | 0.14 |
| 31 | 0.28 | 0.30 | 0.54 | 0.24 | 0.29 | 0.42 | 0.46 | 0.41 | 0.67 | 0.33 | 0.39 | 0.14 |
| 32 | 0.33 | 0.29 | 0.52 | 0.34 | 0.30 | 0.43 | 0.38 | 0.41 | 0.59 | 0.36 | 0.40 | 0.10 |
| 33 | 0.30 | 0.28 | 0.49 | 0.35 | 0.19 | 0.43 | 0.45 | 0.38 | 0.57 | 0.29 | 0.37 | 0.11 |
| 34 | 0.28 | 0.33 | 0.48 | 0.36 | 0.24 | 0.43 | 0.49 | 0.38 | 0.62 | 0.32 | 0.39 | 0.11 |
| 35 | 0.27 | 0.30 | 0.48 | 0.33 | 0.23 | 0.47 | 0.49 | 0.37 | 0.74 | 0.34 | 0.40 | 0.15 |
| 36 | 0.27 | 0.33 | 0.58 | 0.38 | 0.26 | 0.43 | 0.42 | 0.36 | 0.69 | 0.35 | 0.41 | 0.14 |
| 37 | 0.26 | 0.34 | 0.52 | 0.39 | 0.25 | 0.40 | 0.41 | 0.38 | 0.63 | 0.36 | 0.40 | 0.11 |
| 38 | 0.27 | 0.33 | 0.53 | 0.31 | 0.15 | 0.44 | 0.48 | 0.36 | 0.67 | 0.37 | 0.39 | 0.15 |
| 39 | 0.15 | 0.30 | 0.53 | 0.33 | 0.15 | 0.49 | 0.46 | 0.36 | 0.68 | 0.36 | 0.38 | 0.16 |
| 40 | 0.15 | 0.31 | 0.53 | 0.37 | 0.10 | 0.53 | 0.44 | 0.34 | 0.75 | 0.38 | 0.39 | 0.19 |

**Table S31.** The effect of single bolus injection of bivalirudin in 0.75 mg/kg dose on thrombus growth in the model of induced thrombosis.

| **Time, min** | **Thrombus area, mm^2^** | | | | | | | | | **Mean value** | **St. dev.** |
| --- | --- | --- | --- | --- | --- | --- | --- | --- | --- | --- | --- |
|  | *223* | *224* | *225* | *226* | *227* | *228* | *229* | *230* | *231* |  |  |
| 3 | 0.07 | 0.10 | 0.22 | 0.06 | 0.25 | 0.25 | 0.17 | 0.30 | 0.03 | 0.16 | 0.10 |
| 4 | 0.07 | 0.13 | 0.23 | 0.13 | 0.26 | 0.26 | 0.19 | 0.22 | 0.17 | 0.19 | 0.06 |
| 5 | 0.08 | 0.15 | 0.18 | 0.16 | 0.26 | 0.28 | 0.26 | 0.22 | 0.16 | 0.19 | 0.07 |
| 6 | 0.03 | 0.16 | 0.07 | 0.12 | 0.21 | 0.28 | 0.17 | 0.12 | 0.23 | 0.15 | 0.08 |
| 7 | 0.05 | 0.17 | 0.05 | 0.15 | 0.18 | 0.26 | 0.19 | 0.15 | 0.19 | 0.15 | 0.07 |
| 8 | 0.06 | 0.15 | 0.10 | 0.15 | 0.22 | 0.26 | 0.07 | 0.16 | 0.06 | 0.14 | 0.07 |
| 9 | 0.05 | 0.17 | 0.05 | 0.11 | 0.23 | 0.24 | 0.13 | 0.21 | 0.07 | 0.14 | 0.08 |
| 10 | 0.08 | 0.17 | 0.05 | 0.15 | 0.19 | 0.23 | 0.13 | 0.17 | 0.05 | 0.14 | 0.06 |
| 11 | 0.08 | 0.14 | 0.01 | 0.11 | 0.17 | 0.28 | 0.14 | 0.20 | 0.05 | 0.13 | 0.08 |
| 12 | 0.06 | 0.13 | 0.01 | 0.15 | 0.15 | 0.27 | 0.14 | 0.21 | 0.06 | 0.13 | 0.08 |
| 13 | 0.08 | 0.11 | 0.01 | 0.15 | 0.13 | 0.24 | 0.16 | 0.21 | 0.04 | 0.13 | 0.08 |
| 14 | 0.05 | 0.18 | 0.03 | 0.16 | 0.22 | 0.27 | 0.22 | 0.25 | 0.07 | 0.16 | 0.09 |
| 15 | 0.08 | 0.15 | 0.03 | 0.16 | 0.14 | 0.30 | 0.29 | 0.27 | 0.09 | 0.17 | 0.10 |
| 16 | 0.07 | 0.14 | 0.07 | 0.17 | 0.20 | 0.37 | 0.24 | 0.26 | 0.06 | 0.18 | 0.11 |
| 17 | 0.07 | 0.14 | 0.06 | 0.19 | 0.15 | 0.34 | 0.28 | 0.27 | 0.09 | 0.18 | 0.10 |
| 18 | 0.08 | 0.20 | 0.02 | 0.17 | 0.12 | 0.32 | 0.30 | 0.28 | 0.08 | 0.17 | 0.11 |
| 19 | 0.11 | 0.23 | 0.05 | 0.17 | 0.19 | 0.33 | 0.36 | 0.27 | 0.10 | 0.20 | 0.10 |
| 20 | 0.10 | 0.31 | 0.06 | 0.15 | 0.23 | 0.30 | 0.33 | 0.26 | 0.11 | 0.21 | 0.10 |
| 21 | 0.10 | 0.27 | 0.09 | 0.22 | 0.21 | 0.33 | 0.33 | 0.27 | 0.13 | 0.22 | 0.09 |
| 22 | 0.09 | 0.32 | 0.10 | 0.20 | 0.11 | 0.39 | 0.33 | 0.31 | 0.08 | 0.22 | 0.12 |
| 23 | 0.09 | 0.27 | 0.09 | 0.20 | 0.20 | 0.39 | 0.34 | 0.31 | 0.11 | 0.22 | 0.11 |
| 24 | 0.08 | 0.33 | 0.17 | 0.21 | 0.21 | 0.43 | 0.49 | 0.29 | 0.11 | 0.26 | 0.14 |
| 25 | 0.09 | 0.35 | 0.10 | 0.24 | 0.18 | 0.45 | 0.53 | 0.35 | 0.11 | 0.27 | 0.16 |
| 26 | 0.08 | 0.34 | 0.10 | 0.24 | 0.19 | 0.47 | 0.44 | 0.28 | 0.14 | 0.25 | 0.14 |
| 27 | 0.09 | 0.29 | 0.09 | 0.22 | 0.23 | 0.47 | 0.44 | 0.35 | 0.14 | 0.26 | 0.14 |
| 28 | 0.10 | 0.34 | 0.10 | 0.24 | 0.22 | 0.35 | 0.49 | 0.33 | 0.10 | 0.25 | 0.14 |
| 29 | 0.08 | 0.37 | 0.15 | 0.24 | 0.23 | 0.40 | 0.46 | 0.33 | 0.11 | 0.26 | 0.14 |
| 30 | 0.08 | 0.37 | 0.15 | 0.24 | 0.23 | 0.41 | 0.50 | 0.34 | 0.08 | 0.27 | 0.15 |
| 31 | 0.07 | 0.40 | 0.16 | 0.21 | 0.24 | 0.41 | 0.42 | 0.31 | 0.07 | 0.25 | 0.14 |
| 32 | 0.08 | 0.33 | 0.13 | 0.20 | 0.25 | 0.41 | 0.43 | 0.28 | 0.11 | 0.25 | 0.13 |
| 33 | 0.08 | 0.36 | 0.15 | 0.28 | 0.25 | 0.40 | 0.51 | 0.26 | 0.12 | 0.27 | 0.14 |
| 34 | 0.10 | 0.29 | 0.13 | 0.22 | 0.23 | 0.35 | 0.56 | 0.26 | 0.11 | 0.25 | 0.14 |
| 35 | 0.07 | 0.30 | 0.13 | 0.25 | 0.24 | 0.37 | 0.64 | 0.29 | 0.13 | 0.27 | 0.17 |
| 36 | 0.10 | 0.33 | 0.11 | 0.24 | 0.22 | 0.36 | 0.61 | 0.29 | 0.11 | 0.26 | 0.16 |
| 37 | 0.10 | 0.35 | 0.13 | 0.24 | 0.22 | 0.40 | 0.69 | 0.25 | 0.12 | 0.28 | 0.19 |
| 38 | 0.09 | 0.34 | 0.17 | 0.25 | 0.19 | 0.46 | 0.60 | 0.23 | 0.11 | 0.27 | 0.17 |
| 39 | 0.09 | 0.45 | 0.15 | 0.25 | 0.18 | 0.46 | 0.68 | 0.26 | 0.10 | 0.29 | 0.20 |
| 40 | 0.13 | 0.45 | 0.16 | 0.25 | 0.22 | 0.48 | 0.53 | 0.26 | 0.10 | 0.29 | 0.16 |

**Table S32.** The effect of single bolus injection of aptamer RA-36 in 7.0 mg/kg dose on thrombus growth in the model of induced thrombosis.

| **Time, min** | **Thrombus area, mm^2^** | | | | | | | | | | **Mean value** | **St. dev.** |
| --- | --- | --- | --- | --- | --- | --- | --- | --- | --- | --- | --- | --- |
|  | *232* | *233* | *234* | *235* | *236* | *237* | *238* | *239* | *240* | *241* |  |  |
| 3 | 0.24 | 0.14 | 0.29 | 0.23 | 0.08 | 0.27 | 0.18 | 0.18 | 0.28 | 0.23 | 0.21 | 0.07 |
| 4 | 0.30 | 0.12 | 0.20 | 0.30 | 0.15 | 0.21 | 0.18 | 0.20 | 0.31 | 0.25 | 0.22 | 0.07 |
| 5 | 0.26 | 0.15 | 0.26 | 0.26 | 0.14 | 0.14 | 0.21 | 0.19 | 0.30 | 0.24 | 0.22 | 0.06 |
| 6 | 0.25 | 0.16 | 0.22 | 0.24 | 0.10 | 0.08 | 0.19 | 0.18 | 0.32 | 0.15 | 0.19 | 0.07 |
| 7 | 0.28 | 0.17 | 0.22 | 0.23 | 0.11 | 0.12 | 0.08 | 0.18 | 0.41 | 0.16 | 0.20 | 0.10 |
| 8 | 0.31 | 0.18 | 0.29 | 0.23 | 0.17 | 0.09 | 0.07 | 0.23 | 0.40 | 0.24 | 0.22 | 0.10 |
| 9 | 0.35 | 0.20 | 0.33 | 0.25 | 0.12 | 0.08 | 0.06 | 0.23 | 0.44 | 0.25 | 0.23 | 0.12 |
| 10 | 0.36 | 0.21 | 0.34 | 0.34 | 0.14 | 0.19 | 0.14 | 0.25 | 0.42 | 0.24 | 0.26 | 0.10 |
| 11 | 0.29 | 0.23 | 0.39 | 0.32 | 0.16 | 0.13 | 0.17 | 0.23 | 0.40 | 0.35 | 0.27 | 0.10 |
| 12 | 0.33 | 0.29 | 0.39 | 0.26 | 0.23 | 0.27 | 0.23 | 0.25 | 0.47 | 0.38 | 0.31 | 0.08 |
| 13 | 0.33 | 0.35 | 0.49 | 0.25 | 0.30 | 0.23 | 0.33 | 0.22 | 0.40 | 0.40 | 0.33 | 0.09 |
| 14 | 0.42 | 0.34 | 0.46 | 0.25 | 0.32 | 0.33 | 0.34 | 0.21 | 0.54 | 0.41 | 0.36 | 0.10 |
| 15 | 0.38 | 0.44 | 0.34 | 0.26 | 0.30 | 0.32 | 0.35 | 0.24 | 0.43 | 0.44 | 0.35 | 0.07 |
| 16 | 0.40 | 0.50 | 0.33 | 0.24 | 0.29 | 0.38 | 0.25 | 0.26 | 0.46 | 0.41 | 0.35 | 0.09 |
| 17 | 0.44 | 0.51 | 0.35 | 0.31 | 0.32 | 0.46 | 0.25 | 0.28 | 0.47 | 0.45 | 0.38 | 0.09 |
| 18 | 0.58 | 0.55 | 0.41 | 0.36 | 0.32 | 0.37 | 0.26 | 0.30 | 0.76 | 0.43 | 0.43 | 0.16 |
| 19 | 0.67 | 0.53 | 0.40 | 0.33 | 0.31 | 0.37 | 0.25 | 0.30 | 0.89 | 0.41 | 0.45 | 0.20 |
| 20 | 0.80 | 0.56 | 0.40 | 0.34 | 0.32 | 0.36 | 0.34 | 0.31 | 0.85 | 0.44 | 0.47 | 0.20 |
| 21 | 0.86 | 0.61 | 0.42 | 0.24 | 0.37 | 0.35 | 0.31 | 0.27 | 0.96 | 0.46 | 0.50 | 0.20 |
| 22 | 0.65 | 0.63 | 0.41 | 0.22 | 0.32 | 0.36 | 0.34 | 0.26 | 0.80 | 0.45 | 0.44 | 0.19 |
| 23 | 0.62 | 0.63 | 0.42 | 0.24 | 0.34 | 0.39 | 0.39 | 0.29 | 0.97 | 0.41 | 0.47 | 0.21 |
| 24 | 0.63 | 0.61 | 0.41 | 0.23 | 0.34 | 0.40 | 0.41 | 0.29 | 0.92 | 0.40 | 0.46 | 0.20 |
| 25 | 0.63 | 0.63 | 0.45 | 0.22 | 0.28 | 0.41 | 0.42 | 0.28 | 1.01 | 0.39 | 0.50 | 0.20 |
| 26 | 0.73 | 0.61 | 0.45 | 0.24 | 0.31 | 0.38 | 0.38 | 0.26 | 1.19 | 0.36 | 0.50 | 0.30 |
| 27 | 0.66 | 0.64 | 0.45 | 0.22 | 0.30 | 0.37 | 0.42 | 0.30 | 1.05 | 0.40 | 0.50 | 0.20 |
| 28 | 0.51 | 0.66 | 0.46 | 0.23 | 0.50 | 0.35 | 0.39 | 0.31 | 1.11 | 0.47 | 0.50 | 0.20 |
| 29 | 0.59 | 0.67 | 0.43 | 0.29 | 0.57 | 0.37 | 0.48 | 0.29 | 1.06 | 0.38 | 0.50 | 0.20 |
| 30 | 0.65 | 0.67 | 0.45 | 0.30 | 0.53 | 0.40 | 0.42 | 0.27 | 1.01 | 0.44 | 0.50 | 0.20 |
| 31 | 0.59 | 0.75 | 0.44 | 0.31 | 0.48 | 0.37 | 0.43 | 0.26 | 0.99 | 0.39 | 0.50 | 0.20 |
| 32 | 0.60 | 0.71 | 0.42 | 0.33 | 0.49 | 0.38 | 0.46 | 0.26 | 1.01 | 0.45 | 0.50 | 0.20 |
| 33 | 0.63 | 0.74 | 0.44 | 0.29 | 0.49 | 0.47 | 0.40 | 0.31 | 0.95 | 0.39 | 0.50 | 0.20 |
| 34 | 0.77 | 0.71 | 0.47 | 0.29 | 0.62 | 0.45 | 0.45 | 0.29 | 0.92 | 0.43 | 0.50 | 0.20 |
| 35 | 0.64 | 0.68 | 0.42 | 0.23 | 0.47 | 0.44 | 0.49 | 0.36 | 0.91 | 0.45 | 0.51 | 0.19 |
| 36 | 0.68 | 0.72 | 0.43 | 0.24 | 0.48 | 0.41 | 0.49 | 0.35 | 0.96 | 0.44 | 0.50 | 0.20 |
| 37 | 0.67 | 0.71 | 0.45 | 0.22 | 0.46 | 0.43 | 0.50 | 0.35 | 0.92 | 0.42 | 0.51 | 0.20 |
| 38 | 0.75 | 0.70 | 0.46 | 0.24 | 0.52 | 0.42 | 0.53 | 0.33 | 0.86 | 0.44 | 0.52 | 0.19 |
| 39 | 0.57 | 0.68 | 0.43 | 0.24 | 0.42 | 0.36 | 0.53 | 0.29 | 0.81 | 0.45 | 0.48 | 0.18 |
| 40 | 0.63 | 0.70 | 0.45 | 0.23 | 0.51 | 0.30 | 0.55 | 0.28 | 1.00 | 0.45 | 0.50 | 0.20 |

**Table S33.** The effect of single bolus injection of aptamer RA-36 in 35 mg/kg dose on thrombus growth in the model of induced thrombosis.

| **Time, min** | **Thrombus area, mm^2^** | | | | | | | | | | **Mean value** | **St. dev.** |
| --- | --- | --- | --- | --- | --- | --- | --- | --- | --- | --- | --- | --- |
|  | *242* | *243* | *244* | *245* | *246* | *247* | *248* | *249* | *250* | *251* |  |  |
| 3 | 0.05 | 0.00 | 0.14 | 0.00 | 0.00 | 0.05 | 0.18 | 0.32 | 0.29 | 0.23 | 0.13 | 0.12 |
| 4 | 0.07 | 0.00 | 0.28 | 0.00 | 0.00 | 0.09 | 0.18 | 0.25 | 0.34 | 0.23 | 0.14 | 0.13 |
| 5 | 0.07 | 0.00 | 0.13 | 0.00 | 0.00 | 0.12 | 0.17 | 0.10 | 0.34 | 0.28 | 0.12 | 0.12 |
| 6 | 0.05 | 0.00 | 0.11 | 0.00 | 0.00 | 0.15 | 0.17 | 0.04 | 0.35 | 0.04 | 0.09 | 0.11 |
| 7 | 0.04 | 0.00 | 0.09 | 0.00 | 0.00 | 0.17 | 0.19 | 0.06 | 0.36 | 0.04 | 0.09 | 0.11 |
| 8 | 0.04 | 0.00 | 0.19 | 0.00 | 0.00 | 0.18 | 0.20 | 0.07 | 0.35 | 0.04 | 0.11 | 0.12 |
| 9 | 0.09 | 0.00 | 0.25 | 0.00 | 0.00 | 0.26 | 0.21 | 0.07 | 0.33 | 0.26 | 0.15 | 0.13 |
| 10 | 0.09 | 0.00 | 0.33 | 0.02 | 0.00 | 0.22 | 0.20 | 0.06 | 0.36 | 0.37 | 0.16 | 0.15 |
| 11 | 0.17 | 0.02 | 0.30 | 0.01 | 0.00 | 0.20 | 0.19 | 0.05 | 0.34 | 0.36 | 0.16 | 0.14 |
| 12 | 0.18 | 0.08 | 0.30 | 0.01 | 0.00 | 0.19 | 0.18 | 0.11 | 0.36 | 0.41 | 0.18 | 0.14 |
| 13 | 0.08 | 0.03 | 0.59 | 0.02 | 0.00 | 0.22 | 0.20 | 0.11 | 0.33 | 0.46 | 0.20 | 0.20 |
| 14 | 0.10 | 0.02 | 0.66 | 0.00 | 0.00 | 0.24 | 0.19 | 0.10 | 0.34 | 0.43 | 0.20 | 0.20 |
| 15 | 0.10 | 0.00 | 0.69 | 0.00 | 0.00 | 0.22 | 0.20 | 0.06 | 0.35 | 0.43 | 0.20 | 0.20 |
| 16 | 0.13 | 0.00 | 0.68 | 0.00 | 0.00 | 0.27 | 0.19 | 0.08 | 0.36 | 0.51 | 0.20 | 0.20 |
| 17 | 0.16 | 0.00 | 0.62 | 0.00 | 0.00 | 0.21 | 0.19 | 0.07 | 0.34 | 0.51 | 0.20 | 0.20 |
| 18 | 0.15 | 0.03 | 0.47 | 0.00 | 0.00 | 0.27 | 0.20 | 0.07 | 0.37 | 0.58 | 0.20 | 0.20 |
| 19 | 0.16 | 0.01 | 0.78 | 0.00 | 0.00 | 0.26 | 0.21 | 0.04 | 0.35 | 0.71 | 0.30 | 0.30 |
| 20 | 0.17 | 0.01 | 0.70 | 0.00 | 0.00 | 0.25 | 0.21 | 0.08 | 0.34 | 0.74 | 0.30 | 0.30 |
| 21 | 0.18 | 0.01 | 0.70 | 0.00 | 0.00 | 0.25 | 0.20 | 0.05 | 0.39 | 0.84 | 0.30 | 0.30 |
| 22 | 0.16 | 0.01 | 0.69 | 0.00 | 0.00 | 0.26 | 0.20 | 0.06 | 0.41 | 1.00 | 0.30 | 0.30 |
| 23 | 0.14 | 0.00 | 0.69 | 0.00 | 0.00 | 0.26 | 0.20 | 0.05 | 0.35 | 1.02 | 0.30 | 0.30 |
| 24 | 0.13 | 0.00 | 0.69 | 0.00 | 0.00 | 0.26 | 0.20 | 0.05 | 0.34 | 0.95 | 0.30 | 0.30 |
| 25 | 0.16 | 0.00 | 0.78 | 0.00 | 0.00 | 0.30 | 0.19 | 0.04 | 0.40 | 0.96 | 0.30 | 0.30 |
| 26 | 0.16 | 0.00 | 0.69 | 0.00 | 0.00 | 0.32 | 0.17 | 0.06 | 0.36 | 0.88 | 0.30 | 0.30 |
| 27 | 0.17 | 0.00 | 0.78 | 0.00 | 0.00 | 0.26 | 0.19 | 0.05 | 0.39 | 0.81 | 0.30 | 0.30 |
| 28 | 0.13 | 0.00 | 0.70 | 0.00 | 0.00 | 0.29 | 0.19 | 0.06 | 0.39 | 0.82 | 0.30 | 0.30 |
| 29 | 0.12 | 0.00 | 0.71 | 0.00 | 0.00 | 0.30 | 0.22 | 0.07 | 0.39 | 0.86 | 0.30 | 0.30 |
| 30 | 0.15 | 0.00 | 0.69 | 0.00 | 0.00 | 0.28 | 0.20 | 0.06 | 0.36 | 0.81 | 0.30 | 0.30 |
| 31 | 0.15 | 0.00 | 0.71 | 0.00 | 0.00 | 0.30 | 0.20 | 0.07 | 0.35 | 0.83 | 0.30 | 0.30 |
| 32 | 0.14 | 0.00 | 0.71 | 0.00 | 0.00 | 0.28 | 0.18 | 0.06 | 0.37 | 0.80 | 0.30 | 0.30 |
| 33 | 0.15 | 0.00 | 0.71 | 0.00 | 0.00 | 0.29 | 0.19 | 0.07 | 0.38 | 0.90 | 0.30 | 0.30 |
| 34 | 0.14 | 0.00 | 0.69 | 0.00 | 0.00 | 0.27 | 0.19 | 0.05 | 0.36 | 0.88 | 0.30 | 0.30 |
| 35 | 0.14 | 0.00 | 0.71 | 0.00 | 0.00 | 0.27 | 0.19 | 0.07 | 0.37 | 1.06 | 0.30 | 0.30 |
| 36 | 0.14 | 0.00 | 0.71 | 0.00 | 0.00 | 0.28 | 0.20 | 0.07 | 0.36 | 0.99 | 0.30 | 0.30 |
| 37 | 0.14 | 0.00 | 0.67 | 0.00 | 0.00 | 0.28 | 0.22 | 0.06 | 0.37 | 1.02 | 0.30 | 0.30 |
| 38 | 0.14 | 0.00 | 0.70 | 0.00 | 0.00 | 0.27 | 0.21 | 0.08 | 0.36 | 0.91 | 0.30 | 0.30 |
| 39 | 0.14 | 0.00 | 0.70 | 0.00 | 0.00 | 0.30 | 0.20 | 0.06 | 0.36 | 1.10 | 0.30 | 0.40 |
| 40 | 0.14 | 0.00 | 0.71 | 0.00 | 0.00 | 0.25 | 0.22 | 0.06 | 0.37 | 1.00 | 0.30 | 0.30 |

**Table S34.** The effect of single bolus injection of aptamer RA-36 in 70 mg/kg dose on thrombus growth in the model of induced thrombosis.

| **Time, min** | **Thrombus area, mm^2^** | | | | | | | | | | **Mean value** | **St. dev.** |
| --- | --- | --- | --- | --- | --- | --- | --- | --- | --- | --- | --- | --- |
|  | *252* | *253* | *254* | *255* | *256* | *257* | *258* | *259* | *260* | *261* |  |  |
| 3 | 0.00 | 0.00 | 0.00 | 0.00 | 0.00 | 0.13 | 0.08 | 0.21 | 0.15 | 0.22 | 0.08 | 0.09 |
| 4 | 0.00 | 0.29 | 0.00 | 0.00 | 0.00 | 0.18 | 0.06 | 0.18 | 0.15 | 0.23 | 0.11 | 0.11 |
| 5 | 0.00 | 0.28 | 0.00 | 0.05 | 0.00 | 0.13 | 0.09 | 0.20 | 0.16 | 0.13 | 0.10 | 0.09 |
| 6 | 0.00 | 0.27 | 0.00 | 0.15 | 0.00 | 0.14 | 0.07 | 0.23 | 0.16 | 0.06 | 0.11 | 0.10 |
| 7 | 0.00 | 0.16 | 0.00 | 0.00 | 0.00 | 0.11 | 0.07 | 0.21 | 0.14 | 0.11 | 0.08 | 0.08 |
| 8 | 0.00 | 0.27 | 0.00 | 0.00 | 0.00 | 0.14 | 0.07 | 0.18 | 0.15 | 0.07 | 0.09 | 0.10 |
| 9 | 0.00 | 0.26 | 0.03 | 0.00 | 0.00 | 0.14 | 0.05 | 0.19 | 0.15 | 0.11 | 0.09 | 0.09 |
| 10 | 0.00 | 0.32 | 0.04 | 0.00 | 0.00 | 0.19 | 0.09 | 0.20 | 0.18 | 0.09 | 0.11 | 0.11 |
| 11 | 0.00 | 0.37 | 0.03 | 0.00 | 0.00 | 0.12 | 0.08 | 0.19 | 0.17 | 0.09 | 0.10 | 0.12 |
| 12 | 0.00 | 0.39 | 0.02 | 0.00 | 0.00 | 0.13 | 0.09 | 0.21 | 0.15 | 0.09 | 0.11 | 0.12 |
| 13 | 0.00 | 0.38 | 0.04 | 0.00 | 0.00 | 0.11 | 0.16 | 0.19 | 0.18 | 0.08 | 0.11 | 0.12 |
| 14 | 0.00 | 0.37 | 0.03 | 0.00 | 0.00 | 0.14 | 0.19 | 0.20 | 0.17 | 0.08 | 0.12 | 0.12 |
| 15 | 0.00 | 0.45 | 0.04 | 0.00 | 0.00 | 0.11 | 0.18 | 0.22 | 0.16 | 0.09 | 0.13 | 0.14 |
| 16 | 0.00 | 0.47 | 0.00 | 0.00 | 0.00 | 0.12 | 0.20 | 0.28 | 0.16 | 0.08 | 0.13 | 0.15 |
| 17 | 0.00 | 0.37 | 0.00 | 0.00 | 0.00 | 0.15 | 0.17 | 0.29 | 0.15 | 0.07 | 0.12 | 0.13 |
| 18 | 0.00 | 0.47 | 0.00 | 0.00 | 0.00 | 0.13 | 0.16 | 0.20 | 0.16 | 0.09 | 0.12 | 0.15 |
| 19 | 0.00 | 0.47 | 0.00 | 0.00 | 0.00 | 0.14 | 0.18 | 0.22 | 0.17 | 0.10 | 0.13 | 0.15 |
| 20 | 0.00 | 0.47 | 0.00 | 0.00 | 0.00 | 0.15 | 0.17 | 0.26 | 0.16 | 0.09 | 0.13 | 0.15 |
| 21 | 0.00 | 0.45 | 0.00 | 0.00 | 0.00 | 0.12 | 0.17 | 0.28 | 0.16 | 0.08 | 0.13 | 0.15 |
| 22 | 0.00 | 0.41 | 0.00 | 0.00 | 0.00 | 0.13 | 0.17 | 0.29 | 0.17 | 0.12 | 0.13 | 0.14 |
| 23 | 0.00 | 0.45 | 0.00 | 0.00 | 0.00 | 0.14 | 0.16 | 0.29 | 0.18 | 0.10 | 0.13 | 0.15 |
| 24 | 0.00 | 0.43 | 0.00 | 0.00 | 0.00 | 0.15 | 0.19 | 0.32 | 0.17 | 0.09 | 0.13 | 0.15 |
| 25 | 0.00 | 0.35 | 0.00 | 0.00 | 0.00 | 0.18 | 0.16 | 0.31 | 0.17 | 0.11 | 0.13 | 0.13 |
| 26 | 0.00 | 0.36 | 0.00 | 0.00 | 0.00 | 0.12 | 0.17 | 0.28 | 0.17 | 0.12 | 0.12 | 0.13 |
| 27 | 0.00 | 0.36 | 0.00 | 0.00 | 0.00 | 0.17 | 0.16 | 0.33 | 0.17 | 0.10 | 0.13 | 0.14 |
| 28 | 0.00 | 0.40 | 0.00 | 0.00 | 0.00 | 0.16 | 0.15 | 0.38 | 0.16 | 0.10 | 0.13 | 0.15 |
| 29 | 0.00 | 0.42 | 0.00 | 0.00 | 0.00 | 0.14 | 0.10 | 0.38 | 0.22 | 0.11 | 0.14 | 0.16 |
| 30 | 0.00 | 0.38 | 0.00 | 0.00 | 0.00 | 0.17 | 0.12 | 0.38 | 0.24 | 0.11 | 0.14 | 0.15 |
| 31 | 0.00 | 0.37 | 0.00 | 0.00 | 0.00 | 0.15 | 0.10 | 0.40 | 0.22 | 0.11 | 0.14 | 0.15 |
| 32 | 0.00 | 0.41 | 0.00 | 0.00 | 0.00 | 0.17 | 0.13 | 0.40 | 0.25 | 0.10 | 0.15 | 0.16 |
| 33 | 0.00 | 0.45 | 0.00 | 0.00 | 0.00 | 0.16 | 0.16 | 0.41 | 0.25 | 0.10 | 0.15 | 0.17 |
| 34 | 0.00 | 0.47 | 0.00 | 0.00 | 0.00 | 0.13 | 0.16 | 0.41 | 0.24 | 0.10 | 0.15 | 0.17 |
| 35 | 0.00 | 0.45 | 0.00 | 0.00 | 0.00 | 0.17 | 0.15 | 0.35 | 0.24 | 0.08 | 0.14 | 0.16 |
| 36 | 0.00 | 0.47 | 0.00 | 0.00 | 0.00 | 0.15 | 0.17 | 0.31 | 0.21 | 0.10 | 0.14 | 0.16 |
| 37 | 0.00 | 0.41 | 0.00 | 0.00 | 0.00 | 0.14 | 0.14 | 0.34 | 0.27 | 0.10 | 0.14 | 0.15 |
| 38 | 0.00 | 0.47 | 0.00 | 0.00 | 0.00 | 0.16 | 0.17 | 0.33 | 0.24 | 0.07 | 0.14 | 0.16 |
| 39 | 0.00 | 0.41 | 0.00 | 0.00 | 0.00 | 0.15 | 0.16 | 0.47 | 0.28 | 0.09 | 0.16 | 0.18 |
| 40 | 0.00 | 0.41 | 0.00 | 0.00 | 0.00 | 0.17 | 0.17 | 0.36 | 0.28 | 0.09 | 0.15 | 0.16 |

**Table S35.** Pharmacokinetics of intravenously bolus injected aptamer RA-36 (7.0 mg/kg dose in rats). Elimination of the aptamer from blood.

| **Time, min** | **Content of aptamer RA-36 in blood, µg/ml** | | | | | | | | **Mean value** | **St. dev.** |
| --- | --- | --- | --- | --- | --- | --- | --- | --- | --- | --- |
|  | *262* | *263* | *264* | *265* | *266* | *267* | *268* | *269* |  |  |
| 1 | 36.75 | 36.24 | 19.86 | 38.00 | 40.59 | 54.87 | 28.59 | 35.26 | 40 | 10 |
| 3 | 18.87 | 16.51 | 8.92 | 9.29 | 15.16 | 18.48 | 8.76 | 11.54 | 13 | 4 |
| 5 | 11.55 | 8.41 | 6.05 | 5.66 | 11.64 | 7.99 | 5.56 | 7.38 | 8 | 2 |
| 7 | 6.83 | 6.60 | 4.87 | 4.66 | 6.94 | 5.91 | 4.65 | 5.90 | 5.8 | 1.0 |
| 10 | 5.09 | 5.85 | 3.60 | 4.38 | 6.03 | 5.64 | 4.07 | 5.91 | 5.1 | 0.9 |
| 15 | 10.14 | 6.81 | 4.93 | 3.44 | 4.67 | 4.24 | 3.19 | 4.66 | 5 | 2 |
| 20 | 2.77 | 3.88 | 3.33 | 2.54 | 2.02 | 3.61 | 2.33 | 3.50 | 3.0 | 0.7 |
| 30 | 2.49 | 3.07 | 4.29 | 2.66 | 3.28 | 2.52 | 2.30 | 2.80 | 2.9 | 0.6 |
| 40 | 1.71 | 1.91 | 2.24 | 2.17 | 2.79 | 2.06 | 1.81 | 2.32 | 2.1 | 0.3 |

**Table S36.** Pharmacokinetics of intravenously bolus injected aptamer RA-36 (7.0 mg/kg dose in rats). Distribution between tissues and organs 3 minute after the aptamer injection.

| **Tissues** | **Content of aptamer RA-36 in tissues, µg/g** | | | | | | | | | | **Mean value** | **St. dev.** |
| --- | --- | --- | --- | --- | --- | --- | --- | --- | --- | --- | --- | --- |
|  | *270* | *271* | *272* | *273* | *274* | *275* | *276* | *277* | *278* | *279* |  |  |
| Aorta | 20.1 | 22.4 | 24.5 | 15.1 | 19.2 | 20.3 | 14.1 | 23.6 | 19.3 | 14.0 | 19 | 4 |
| Stomach | 1.17 | 1.13 | 1.04 | 0.69 | 1.18 | 0.81 | 1.39 | 1.33 | 0.98 | 1.10 | 1.1 | 0.2 |
| Fat | 0.38 | 0.41 | 0.35 | 0.30 | 0.20 | 0.56 | 0.76 | 0.55 | 0.27 | 0.31 | 0.41 | 0.17 |
| Skin | 4.75 | 4.18 | 5.29 | 2.94 | 4.74 | 4.03 | 5.22 | 4.12 | 3.24 | 3.38 | 4.2 | 0.8 |
| Blood | 7.50 | 7.33 | 12.1 | 7.44 | 6.95 | 10.3 | 9.66 | 8.37 | 9.95 | 14.8 | 9 | 2 |
| Lung | 2.03 | 1.49 | 2.15 | 1.15 | 1.81 | 1.21 | 2.43 | 2.92 | 2.14 | 3.93 | 2.1 | 0.8 |
| Brain | 0.28 | 0.12 | 0.10 | 0.07 | 0.15 | 0.11 | 0.16 | 0.12 | 0.20 | 0.11 | 0.14 | 0.06 |
| Bladder | 4.06 | 1.97 | 2.51 | 2.32 | 2.86 | 2.71 | 3.78 | 3.00 | 2.37 | 2.46 | 2.8 | 0.7 |
| Muscles | 1.20 | 1.01 | 1.18 | 0.64 | 0.97 | 1.19 | 1.27 | 1.19 | 1.11 | 2.57 | 1.2 | 0.5 |
| Adrenal glands | 6.93 | 4.95 | 5.64 | 3.46 | 5.73 | 4.16 | 8.22 | 5.77 | 5.62 | 3.62 | 5.4 | 1.5 |
| Liver | 1.33 | 1.57 | 1.01 | 1.11 | 0.89 | 1.14 | 2.24 | 1.38 | 1.15 | 0.95 | 1.3 | 0.4 |
| Pancreas | 2.41 | 1.70 | 1.38 | 1.70 | 1.41 | 2.28 | 2.01 | 1.70 | 1.90 | 1.89 | 1.8 | 0.3 |
| Esophagus | 6.42 | 6.80 | 6.95 | 5.18 | 3.78 | 6.65 | 6.88 | 9.11 | 6.94 | 4.75 | 6.3 | 1.5 |
| Kidney left | 9.57 | 6.95 | 5.49 | 4.84 | 5.61 | 9.05 | 10.7 | 11.6 | 5.39 | 6.72 | 8 | 2 |
| Kidney right | 10.3 | 6.34 | 5.70 | 4.91 | 4.91 | 9.93 | 11.3 | 12.1 | 5.62 | 9.19 | 8 | 3 |
| Epididymis left | 4.07 | 3.71 | 4.06 | 2.95 | 4.13 | 4.32 | 3.92 | 3.21 | 3.15 | 2.25 | 3.6 | 0.7 |
| Epididymis right | 3.56 | 2.88 | 3.22 | 3.44 | 3.49 | 3.52 | n.d. | 4.26 | 3.03 | 2.61 | 3.3 | 0.5 |
| Prostate | 4.04 | 6.51 | 2.66 | 1.37 | 3.04 | n.d. | 5.75 | 4.42 | 3.85 | 3.60 | 3.9 | 1.6 |
| Spleen | 0.58 | 0.73 | 0.43 | 0.44 | 0.36 | 0.52 | 1.14 | 0.66 | 0.60 | 0.37 | 0.6 | 0.2 |
| Testis left | 0.52 | 0.58 | 0.50 | 0.41 | 0.48 | 0.62 | 0.52 | 0.69 | 0.46 | 0.44 | 0.52 | 0.09 |
| Testis right | 0.50 | 0.58 | 0.54 | 0.41 | 0.53 | 0.52 | 0.44 | 0.65 | 0.55 | 0.39 | 0.51 | 0.08 |
| Heart | 2.28 | 1.80 | 1.42 | 1.20 | 1.85 | 2.43 | 2.53 | 2.25 | 2.00 | 1.45 | 1.9 | 0.5 |
| Thymus | 1.69 | 1.40 | 1.24 | 0.88 | 1.31 | 1.54 | 1.61 | 2.34 | 1.21 | 1.31 | 1.5 | 0.4 |
| Large intestine | 1.64 | 1.57 | 1.35 | 1.27 | 1.04 | 1.99 | 1.59 | 2.01 | 1.13 | 1.36 | 1.5 | 0.3 |
| Small intestine | 1.36 | 0.79 | 0.98 | 0.70 | 1.42 | 1.14 | 1.21 | 1.39 | 1.07 | 0.97 | 1.1 | 0.2 |
| Trachea | 27.3 | 8.97 | 7.76 | 6.93 | 8.47 | 7.67 | 10.9 | 11.2 | 8.55 | 8.36 | 11 | 6 |
